# Incidence and mortality from cervical cancer and other malignancies after treatment of cervical intraepithelial neoplasia: a systematic review and meta-analysis of the literature

**DOI:** 10.1016/j.annonc.2019.11.004

**Published:** 2020-02

**Authors:** I. Kalliala, A. Athanasiou, A.A. Veroniki, G. Salanti, O. Efthimiou, N. Raftis, S. Bowden, M. Paraskevaidi, K. Aro, M. Arbyn, P. Bennett, P. Nieminen, E. Paraskevaidis, M. Kyrgiou

**Affiliations:** 1 Institute of Reproductive and Developmental Biology, Department of Surgery & Cancer, Faculty of Medicine, Imperial College London, London, UK; 2 Department of Obstetrics and Gynaecology, University of Helsinki and Helsinki University Hospital, Helsinki, Finland; 3 Queen Charlotte’s and Chelsea – Hammersmith Hospital, Imperial College Healthcare NHS Trust, London, UK; 4 Department of Primary Education, School of Education, University of Ioannina, Ioannina, Greece; 5 Institute of Social and Preventive Medicine, University of Bern, Bern, Switzerland; 6 Department of Obstetrics and Gynaecology, University Hospital of Ioannina, Ioannina, Greece; 7 Unit of Cancer Epidemiology, Scientific Institute of Public Health, Brussels, Belgium

**Keywords:** cancer incidence, cancer mortality, CIN, conisation, HPV-related cancer, LLETZ

## Abstract

**Background:**

Although local treatments for cervical intraepithelial neoplasia (CIN) are highly effective, it has been reported that treated women remain at increased risk of cervical and other cancers. Our aim is to explore the risk of developing or dying from cervical cancer and other human papillomavirus (HPV)- and non-HPV-related malignancies after CIN treatment and infer its magnitude compared with the general population.

**Materials and methods:**

Design: Systematic review and meta-analysis. Eligibility criteria: Studies with registry-based follow-up reporting cancer incidence or mortality after CIN treatment. Data synthesis: Summary effects were estimated using random-effects models.

**Outcomes:**

Incidence rate of cervical cancer among women treated for CIN (per 100 000 woman-years). Relative risk (RR) of cervical cancer, other HPV-related anogenital tract cancer (vagina, vulva, anus), any cancer, and mortality, for women treated for CIN versus the general population.

**Results:**

Twenty-seven studies were eligible. The incidence rate for cervical cancer after CIN treatment was 39 per 100 000 woman-years (95% confidence interval 22–69). The RR of cervical cancer was elevated compared with the general population (3.30, 2.57–4.24; *P* < 0.001). The RR was higher for women more than 50 years old and remained elevated for at least 20 years after treatment. The RR of vaginal (10.84, 5.58–21.10; *P* < 0.001), vulvar (3.34, 2.39–4.67; *P* < 0.001), and anal cancer (5.11, 2.73–9.55; *P* < 0.001) was also higher. Mortality from cervical/vaginal cancer was elevated, but our estimate was more uncertain (RR 5.04, 0.69–36.94; *P* = 0.073).

**Conclusions:**

Women treated for CIN have a considerably higher risk to be later diagnosed with cervical and other HPV-related cancers compared with the general population. The higher risk of cervical cancer lasts for at least 20 years after treatment and is higher for women more than 50 years of age. Prolonged follow-up beyond the last screening round may be warranted for previously treated women.

## Highlights

• Women previously treated for CIN are at elevated risk of cervical and other HPV-related neoplasms.
• Risk of cervical cancer remains elevated for at least 20 years after treatment.
• Prolonged cervical screening beyond current last screening round at 60 or 65 should be considered.
• Increased awareness for prevention or early cancer detection of other HPV-related malignancies is needed.

## Introduction

The introduction of systematic call and recall screening programmes has resulted in a profound decrease in the incidence and mortality from cervical cancer.^1^ This is because preinvasive precursors [cervical intraepithelial neoplasia (CIN)] can be detected and treated.^2^ Although local cervical treatment of CIN is highly efficacious, treated women continue to represent a high-risk group, as the recurrence rate for high-grade preinvasive disease can be as high as 5%–10%.^3^ Furthermore, and despite increased surveillance, these women have been reported to have a higher risk of invasive cervical cancer than the general population for several years after treatment.^4^, ^5^, ^6^, ^7^, ^8^ The impact of different treatment methods (excisional or ablative) on the risk of future invasion remains largely unclear.

This increase in risk may be caused by persistent or recurrent human papillomavirus (HPV) infections or residual preinvasive disease that can be more difficult to detect and prevent.^9^^,^^10^ It has also been suggested that women who develop CIN constitute a subgroup of infected women who are particularly sensitive to the infection and as a result rapidly acquire reinfections after local treatment. This places them at possibly higher risk of not only cervical, but also other HPV-related neoplasms.

Estimating the relative risk (RR) of cervical cancer in treated women compared with those who were not treated is important for determining the age of the last screening and in formulating follow-up strategies that would allow risk stratification for this high-risk population. In most Western societies, screening for treated women is similar to that of the general population and this is not more intensive or different in length. The age of the last screening, at the age of 60 or 65 years in most countries, has been previously debated,^11^ particularly in the context of a previous local treatment.^8^^,^^12^^,^^13^ In the USA, previously treated women are advised to attend screening for 20 years after treatment, even if this extends beyond the age of 65 years,^13^ although this is not practised in most European settings. High-quality reviews that summarise effect estimates may inform policy makers and allow more tailored screening strategies for this population. Furthermore, awareness of the risk for other HPV-related malignancies may also increase awareness and early detection for these neoplasms.

A systematic review and meta-analysis published 13 years ago reported a 56 per 100 000 woman-years incidence rate (IR) of cervical cancer after CIN treatment, which was thought to be three times greater than the expected rate in the UK.^14^ This meta-analysis included predominantly small studies without centralised follow-up, did not compare to an untreated reference population, and did not explore the risk of non-cervical neoplasms. Since then, there have been several new large population-based studies with nationwide or regionwide follow-up on all cancer-related incidences and mortality. Pooled effect estimates from these studies have not been summarised and reported.

The aim of this review was to the estimate the absolute risk of developing or dying from cervical cancer, and HPV- and non-HPV-related malignancies after CIN treatment, and to further explore how this compares with the risk reported in the general population.

## Materials and methods

We registered our protocol with PROSPERO (CRD42018111659) and followed the PRISMA guidelines for reporting (supplementary Table S1, available at *Annals of Oncology* online).^15^

### Eligibility criteria and outcomes

We included studies reporting on the absolute incidence of cervical cancer or relative incidence and mortality of cervical, HPV-related, or non-HPV-related cancers after local treatment of CIN. Studies were eligible if they used nationwide or regionwide cancer registries as a source of follow-up data, and presented data with at least 5 years of follow-up. We excluded studies where hysterectomy was the primary treatment of CIN in the analysis for cervical cancer incidence. When a subset of the study population had hysterectomy, these women were removed if data were provided separately. If this was not possible, the study was retained if the proportion of women undergoing hysterectomy was less than 10%. For other cancers, we also included studies where the primary CIN treatment was hysterectomy. Studies assessing recurrence rates in women with microinvasive and invasive cervical cancer were excluded. Studies reporting on the outcomes of interest after treatment of both CIN and invasive disease without providing separate data were excluded. In cases of duplicate studies reporting on the same population, we retained the largest study for analysis. We preferred cohort studies to case-control, and those using a ‘lag’ period of at least 6 months between treatment and beginning of cancer incidence follow-up, to avoid the inclusion of cancer cases present but missed at the time of the original treatment. Data from duplicate studies were included in subgroup analyses, where applicable. There were no language or other restrictions.

For each outcome of interest (incidence and mortality of cervical, other HPV-related, non-HPV-related malignancies) we explored both absolute and relative measures compared with the reference population. We focused on the IR defined as the number of cases or deaths per woman-years. We also included RR or hazard ratio (HR) when the reference group included women without CIN, and standardised incidence ratio (SIR) or standardised mortality ratio (SMR) when the general population was used as a reference.

### Literature search, data extraction and assessment of risk of bias

We searched Medline, Embase, and Central from inception to 18 August 2018 for eligible studies (search strategy in supplementary Methods, available at *Annals of Oncology* online). From each study, we extracted, independently and in duplicate, data on the study design, setting, demographics, CIN grades, treatment method used, length of the follow-up, data sources, and outcomes. We also extracted data on the reference population where available. We extracted data on the absolute and relative incidence and mortality for different follow-up time periods, age groups, histological CIN grades, and for each treatment technique when these were provided. Disagreements were resolved by discussion.

Our objective was to explore the absolute and relative incidence of malignancies in women previously treated for CIN versus untreated populations. We therefore used the Quality in Prognosis Studies (QUIPS) tool^16^ (supplementary Methods, available at *Annals of Oncology* online) to explore the risk of bias at the study level independently and in duplicate using six domains: study participation, study attrition, prognostic factor measurement (i.e. treatment of CIN), outcome measurement, adjustment of outcome measurements, and statistical analysis and reporting.

### Data synthesis and assessment of heterogeneity

We fitted a generalised linear mixed model using the log transformation to synthesise the raw IRs of cancer amongst treated women per 100 000 woman-years.^17^^,^^18^ We back-transformed the summary absolute IRs to the original scale to ease interpretation. The between-study variance was estimated using the maximum likelihood method.^19^

Studies reporting on relative cancer incidence and mortality used RR, HR, SIR, or SMR to compare the risks between treated and untreated or the general population. Since the prevalence of CIN treatment or cervical cancer in the general population is low, we considered SIR, HR, and RR to be comparable and therefore meta-analysed them jointly.^20^^,^^21^ The pooled RRs, along with their 95% confidence interval (CI) for cancer incidence and mortality, were estimated using the random-effects model, since we anticipated clinical and methodological heterogeneity. We estimated the summary cancer incidence or mortality RR and its 95% CI using the Hartung-Knapp-Sidik-Jonkman method^22^^,^^23^ to handle meta-analyses with a small number of studies. The between-study variance was estimated using the Paule-Mandel estimator^24^^,^^25^ for the relative estimates, and its 95% CI using the Q-profile approach.^26^ Full details of the analysis are included in supplementary Methods, available at *Annals of Oncology* online.

For all meta-analyses, we quantified the between-study heterogeneity using the *I*^2^ statistic. In meta-analyses of relative effects, we also calculated a 95% CI for the *I*^2^ statistic.^27^, ^28^, ^29^ If there was evidence of substantial heterogeneity and more than two studies were available, the possible reasons for this were investigated through sensitivity and subgroup analyses (supplementary Methods, available at *Annals of Oncology* online). We calculated 95% prediction intervals (PIs) for the absolute and relative treatment effect estimates accounting for between-study heterogeneity to obtain a range in which the predicted true treatment effect in a new study is expected to lie.^30^ We were not able to formally assess for publication bias and small-study effects in our meta-analyses of relative effects due to the small number of studies (<10) in each outcome.^31^ The effect of publication bias in studies assessing prevalence or absolute incidence of cancer is not well established, so we did not perform any such analysis. All analyses were carried out in R V.3.4.3^32^ using the *metafor* package^33^; all forest plots were plotted using the *meta* package.^34^

## Results

### Characteristics of studies

**Figure 1:**
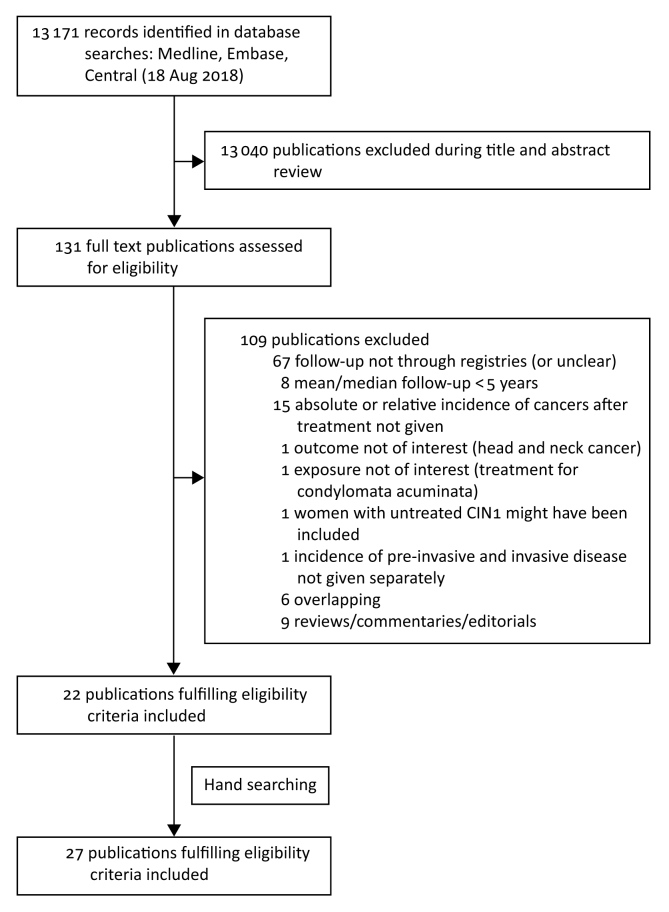
PRISMA flow chart.

We retrieved 13 171 potentially eligible papers, of which 27 publications from 24 cohort studies met the inclusion criteria (Figure 1).^4^^,^^5^^,^^7^^,^^8^^,^^35^, ^36^, ^37^, ^38^, ^39^, ^40^, ^41^, ^42^, ^43^, ^44^, ^45^, ^46^, ^47^, ^48^, ^49^, ^50^, ^51^, ^52^, ^53^, ^54^, ^55^, ^56^, ^57^ The characteristics of the studies are reported in Tables 1 and 2. All studies except two described retrospective cohorts; one was a nested case-control study,^38^ and one reported the pooled analysis for three cohorts within The Netherlands.^45^ The mean or median follow-up time varied from 5 to 27.5 years. The largest study included 150 883 women and the smallest 72. More details are found in supplementary Results, available at *Annals of Oncology* online.

**Table 1:** Characteristics of studies on cervical cancer incidence after treatment of cervical intraepithelial neoplasia (CIN)

Author, year	Country	Study design	*N* treated (women-years)	Treatment method	Degree of treated CIN^a^	Follow-up time (median)^b^	Lag period^c^	*N* cervical cancers among treated	Ascertainment of (a) exposure (i.e. CIN) (b) outcome (i.e. cervical cancer)	Reference population	Effect estimate
Evans, 2003	UK	Retrospective cohort	59 519 (477 069)	NR (<10% had hysterectomy; NR if all women had Tx but most probably did because only CIN3 cases were included)	CIN3	8 years^b^	No	194	(a) TCR (b) Prospective f-u (until 1982) for patients with CIN diagnosed before 1971; retrospective f-u through NHS Cancer Registry for patients with CIN diagnosed after 1970	General female population covered by TCR	SIR
Kalliala, 2005, 2007	Finland	Retrospective cohort	7466 (100 284)	CKC, LLETZ, LC, LA, CT	CIN1–3	11.9 years^b^	0.5 years	22	(a) Records of Helsinki University Hospital (b) Finnish Cancer Registry	General female population from Southern Finland	SIR
Taylor, 2006	USA	Retrospective cohort	56 020 (3 047 808)	NR (because Tx period was 1988–1999, we assumed that hysterectomy was probably carried out in relatively few cases)	CIN3	5 years	No	168	(a) and (b) California Cancer Registry	General female population from California	SIR
Strander, 2007	Sweden	Retrospective cohort	NR (742 765) (only women treated during 1981–2000)	NR (the Swedish Cancer Register does not include data on treatment; we excluded women treated during 1958–1980 when hysterectomy was common for CIN3, and we only included women treated during 1981–2000)	CIN3	NR (17.5 years^b^ for the whole study period)	1 year	327	(a) and (b) Swedish Cancer Registry	General Swedish female population	SIR
Melnikow, 2009	Canada	Retrospective cohort	37 142 (391 892)	CKC, LLETZ, LC, LA, CT	CIN1–3	10.6 years^b^	0.5 years	145	(a) British Columbia Cancer Agency cytology database (b) British Columbia cytology database and British Columbia Cancer Registry	Women (≥21 years old) from British Columbia cytology database with ≥3 consecutive normal smears and no previous Tx for CIN	RR (unadjusted)
McCredie, 2010	New Zealand	Retrospective cohort	72 (1699)	CKC	CIN3	27.5 years	No cancer during the first ∼2.5 years^c^	7	(a) Records of National Women’s Hospital (b) Medical records, histopathological review or cancer and death registries	General female population from New Zealand	SIR
Mitchell, 2002	Australia	Retrospective cohort	6849 (42 463)	NR (hysterectomies were excluded; NR if all women had Tx)	CIN2–3	6.2 years^b^	1 year	15	(a) and (b) VCGS	Women from VCGS with (i) no history of CIN2+, (ii) negative Pap test during the years when CIN2+ was diagnosed in cases, and (iii) available Pap test or histology before study ends	RR (unadjusted)
Jakobsson, 2011	Finland	Retrospective cohort	26 876 (226 510)	Excision (CKC, LLETZ, LC); Ablation (LA, electrocoagulation, CT); other (other excision, cervix amputation etc.)	CIN1–3^a^	8.4 years^b^	f-u started at end of calendar year of CIN treatment^d^	23	(a) National Hospital Discharge Register (b) Finnish Cancer Registry	General Finnish female population	SIR
Kocken, 2011	The Netherlands	Pooled analysis of 2 RCTs and 1 prospective cohort	435 (3464)	CKC, LLETZ	CIN2–3	7.2 years^b^	1.2 years^c^	2	(a) Hospital records (b) Hospital records and The Netherlands nationwide network and registry of histopathology and cytopathology	–	–
Kreimer, 2012	Costa Rica	Retrospective cohort	352 (2082)	CKC, LLETZ	CIN2–3	6 years	No	3	(a) Guanacaste Natural History Study (a population-based study in a rural province) (b) Costa-Rican population-based cancer registry	–	–
Rapiti, 2012	Switzerland	Retrospective cohort	2658 (35 946)	Excision (hysterectomy, CKC, LLETZ, LC); ablation (LA, electrocautery, diathermy, CC); hysterectomy was upon patient’s request; 103 women had no treatment	CIN3 (in 275 women, diagnosis was cytological)	11.1 years	0.5 years	17	(a) and (b) Geneva Cancer Registry	General female population from the Geneva canton	SIR
Rebolj, 2012	The Netherlands	Retrospective cohort	38 956 episodes^e^ (56 956 women-years)	NR (type of treatment not consistently registered; because only patients treated during the 1990s or 2000s were included, we assumed that hysterectomy was carried out in relatively few cases)	CIN1–3	1.5 women-years per episode^b^	∼2 years^f^	20	(a) and (b) Dutch nationwide network and register of histopathology and cytopathology (PALGA)	Women from the whole of The Netherlands with normal smears and without previous CIN	HR (adjusted for year in f-u)
Sand, 2018	Denmark	Retrospective cohort	59 464 (663 925)	Excision (LLETZ, CKC, LC)	CIN3	11.2 years^b^	1 year	237	(a) Pathology Data Bank (b) Danish Cancer Registry	Women from whole Denmark with normal cytology and no previous history of abnormal histology or cytology	HR (adjusted for age and education)

CIN, cervical intraepithelial neoplasia; CC, cold coagulation; CKC, cold knife conisation; CT, cryotherapy; f-u, follow-up; HR, hazard ratio; LA, laser ablation; LC, laser conisation; LLETZ, large loop excision of the transformation zone; *N*, number; NR, not reported; RCT, randomised controlled trial; RR, relative risk; SIR, standardised incidence ratio; TCR, Thames Cancer Registry; Tx, treatment; VCGS, Victorian Cytology Gynaecological Service.

a Some women had cytological diagnosis (or not reported).

b Mean if median is not reported.

c No lag period, but no cancers occurred during the first 6 or 12 months (or we were able to exclude cancers occurring during the first 6 or 12 months after treatment).

d This means that the lag period varied from 0 to 12 months, depending on the month when treatment was carried out.

e Number of women is not reported. Instead, only number of ‘episodes’ is reported. An episode was defined as the following: ‘An episode started with an abnormal smear/biopsy until the f-u of this abnormal smear/biopsy was completed according to guidelines; after the f-u of the abnormal test was completed and woman returned to regular screening, each normal test was considered a separate episode. Additionally, if more than 4 years had passed since the last test, this was considered a new episode.’

f Only women with three consecutive normal cytology smears were included. The interval between last abnormal smear and third consecutive normal smear was allowed to be 1.5—2 years (recommended: 2 years). If abnormal smear, the counter was reset to zero.

**Table 2:** Characteristics of studies on incidence of cancers other than cervical, and on cervico-vaginal cancer mortality after treatment of cervical intraepithelial neoplasia (CIN)

Author, year	Country	Study design	*N* treated	Treatment method	Degree of treated CIN^a^	Follow-up time (median)^b^	Lag period^c^	Outcomes used in meta-analysis	Ascertainment of (a) exposure (b) outcome	Reference population	Effect estimate
Pettersson, 1990	Sweden	Retrospective cohort	56 116	NR (conisation was the usual procedure; hysterectomy was carried out in relatively few cases)	CIN3	8.1 years^b^	1 year	Other cancers: corpus uterus, ovaries, breast	(a) and (b) Swedish National Cancer Registry	General Swedish female population	SIR
Bjorge, 1995	Norway	Retrospective cohort	37 001	NR (conisation was the usual Tx; alternatively, hysterectomy)	CIN3	9.1 years^b^	1 year	Other cancers: overall, corpus uterus, ovaries/fallopian tubes, colon/rectum, breast, lung/bronchus/trachea, female anogenital HPV-related (vagina, vulva, cervix)	(a) and (b) Cancer Registry of Norway	General Norwegian female population	SIR
Frisch, 1995	Denmark	Retrospective cohort	30 294	NR (some women might have received no treatment; hysterectomies might have been included)	CIN3	12.4 years^b^	No	Other cancers: lung	(a) and (b) Danish Cancer Registry	General Danish female population	SIR
Levi, 1996	Switzerland	Retrospective cohort	2190	NR	CIN3	10.1 years^b^	NR	Other cancers: overall, corpus uterus, breast	(a) and (b) Vaud Cancer Registry	General female population from Swiss canton of Vaud	SIR
Evans, 2003	UK	Retrospective and prospective cohort	59 519	NR (<10% had radical surgery; NR if all women had Tx but most probably did because only CIN3 cases were included)	CIN3	8 years^b^	No	Other cancers: overall, vulva, vagina, corpus uterus, ovaries, anus, colon/rectum, breast, lung, cervix/vagina, female anogenital HPV-related (vagina, vulva, cervix, anus)	(a) TCR (b) Prospective f-u (until 1982) for patients with CIN diagnosed before 1971; retrospective f-u through NHS Cancer Registry for patients with CIN diagnosed after 1970	General female population covered by TCR	SIR
Taylor, 2006	USA	Retrospective cohort	56 020	NR	CIN3	5 years	No	Other cancers: ovaries, lung	(a) and (b) California Cancer Registry	General female population from California	SIR
Edgren, 2007	Sweden	Retrospective cohort	125 292	NR (CIN has traditionally been treated by CKC, LC, cryosurgery, LLETZ; 5% were treated with hysterectomy)	CIN3	18.4 years^b^	1 year	Other cancers: vulva, anus, rectum, female anogenital HPV-related (vagina, vulva, anus)	(a) and (b) Swedish Cancer Registry	Women without previous history of CIN3	RR (adjusted for age, calendar period, socioeconomic status and parity)
McCredie, 2010	Australia and New Zealand	Retrospective cohort	72	CKC	CIN3	27.5 years	>∼2.5 years^c^	Other cancers: cervix/vagina Mortality: cervix/vagina	(a) Records of National Women’s Hospital (b) Medical records, histopathological review or cancer and death registries	General female population from New Zealand	SIR
Jakobsson, 2009 for mortality, 2011 for other cancers	Finland	Retrospective cohort	26 876 for other cancers; 25 827 for mortality	Excision (CKC, LLETZ, LC); ablation (LA, electrocoagulation, CT); other (other excision, cervix amputation etc.)	CIN1–3^a^	8.4 years^b^	f-u started at end of calendar year of CIN treatment^d^	Other cancers: overall, vulva, vagina, corpus uterus, ovaries, anus, colon/rectum, breast, lung, cervix/vagina, female anogenital HPV-related (vagina, vulva, cervix, anus) Mortality: cervix	(a) National Hospital Discharge Register (b) Finnish Cancer Registry for other cancers; Finnish Cause-of-Death Register for mortality	General Finnish female population	SIR
Strander, 2007 for other cancers, 2014 for other cancers and mortality	Sweden	Retrospective cohort	132 493 in 2007; 150 883 in 2014	NR (the Swedish Cancer Register does not include date on treatment; hysterectomies have been included)	CIN3	17.5 years^b^ in 2007; 20.9^b^ in 2014	1 year	Other cancers: vagina (in 2007), cervix/vagina (in 2014) Mortality: cervix/vagina	(a) Swedish Cancer Registry (b) Swedish Cancer Registry for other cancers; Swedish Cause-of-Death Register for mortality	General Swedish female population	SIR
Saleem, 2011	USA	Retrospective cohort	124 075	NR (hysterectomies might have been included; NR if all women had Tx but most probably did because only CIS cases were included)	CIN3	NR	1 year	Other cancers: anus	(a) and (b) SEER registry (large population-based registry from 17 regions)	General female population covered by SEER registry	SIR
Gaudet, 2014	Canada	Retrospective cohort	54 320	NR (hysterectomies might have been included; NR if all women had Tx but most probably did because only CIN2+ cases were included)	CIN2–3	10.1 years	0.5 years	Other cancers: vulva, vagina, anus, female anogenital HPV-related (vulva, vagina, anus)	(a) British Columbia Cervical Cancer Screening Program (b) British Columbia Cancer Registry	General female population from British Columbia	SIR
Kirkegard, 2014	Denmark	Retrospective cohort	83 008	Cervical conisation (‘minor surgical procedure’, thus hysterectomies have probably been excluded)	NR (probably any CIN, histological or cytological)	14.9 years	No (lag period only for SIR of cancer incidence in the time window 1–5 years, but not for SIR of overall cancer incidence)	Other cancers: colon/rectum	(a) Danish National Patient Registry (b) Danish Cancer Registry	General Danish female population	SIR
Coffey, 2016	UK	Case-control study (nested case-control in the Million Women Study)	797 vulval cancers in a cohort of 1.3 million women aged 49–65 years; 19/797 had a history of CIN3	NR (hysterectomies might have been included; NR if all women with CIN3 had Tx but most probably did because the likelihood of expectant management of CIN3 is low)	CIN3^a^	13.8 years^b^^,^^e^	3 years	Other cancers: vulva	(a) and (b) UK National Health Service Central Registers (NHSCR) (self-reported data from the recruitment questionnaire were used to define most exposures, but NHSCR was used for ascertainment of CIN3)	Women with vulval cancer but no previous CIN3 diagnosis (case-control study)	RR (adjusted for smoking, alcohol, BMI, diabetes, age at menarche, oral contraceptive use, parity, prior tubal ligation, prior hysterectomy and deprivation)
Sand, 2016	Denmark	Retrospective cohort	156 290	NR (hysterectomies might have been included; some women might have received no Tx)	CIN2–3	13.6 years^b^	1 year	Other cancers: vulva, vagina, anus, female anogenital HPV-related (vulva, vagina, anus)	(a) Danish Cancer Registry & Pathology Data Bank (b) Danish Cancer Registry	Denmark population without history of CIN2/3	HR (adjusted for age and education)
Ebisch, 2017	The Netherlands	Retrospective cohort	89 018	NR (hysterectomies might have been included; NR if all women had Tx but most probably did because only CIN3 cases were included)	CIN3	14	1 year	Other cancers: vulva, vagina, anus, female anogenital HPV-related (vulva, vagina, anus)	(a) and (b) Dutch nationwide registry of histopathology and cytopathology (PALGA; Houten, The Netherlands)	Dutch population without history of CIN3	RR (adjusted for age)

BMI, body mass index; CIN, cervical intraepithelial neoplasia; CIS, carcinoma *in situ*; CKC, cold knife conisation; CT, cryotherapy; f-u, follow-up; HPV, human papillomavirus; HR, hazard ratio; LA, laser ablation; LC, laser conisation; LLETZ, large loop excision of the transformation zone; *N*, number; NR, not reported; RR, relative risk; SIR, standardised incidence ratio; TCR, Thames Cancer Registry; Tx, treatment.

a Some women had cytological diagnosis (or not reported).

b Mean if median is not reported.

c No lag period, but we were able to exclude cancers occurring during the first 6 or 12 months after treatment (or no cancers occurred during the first 6 or 12 months).

d This means that the lag period varied from 0 to 12 months, depending on the month when treatment was carried out.

e This was a nested case-control from the Million Women Study and the mean reported is for a cohort of 1.3 million women aged 49–65 years.

Seven additional studies met the inclusion criteria,^46^^,^^58^, ^59^, ^60^, ^61^, ^62^, ^63^ but were excluded from the main analysis because they presented duplicate results of the same population. Some data from the duplicate studies were used in the subgroup analyses.^46^ The reasons for preferential inclusion of a study over the duplicate on the same population are explained in supplementary Table S2, available at *Annals of Oncology* online.

Thirteen cohorts reported on absolute^7^^,^^8^^,^^35^^,^^41^, ^42^, ^43^^,^^45^, ^46^, ^47^, ^48^, ^49^^,^^51^, ^52^, ^53^ and 10 cohorts on relative^7^^,^^8^^,^^35^^,^^41^^,^^42^^,^^46^^,^^48^^,^^49^^,^^51^, ^52^, ^53^ cervical cancer incidence after CIN treatment. The treatment methods used were reported in eight cohorts.^7^^,^^35^^,^^42^^,^^43^^,^^45^, ^46^, ^47^, ^48^, ^49^ Four cohorts^7^^,^^35^^,^^48^^,^^49^^,^^53^ excluded women treated primarily with hysterectomy (supplementary Results, available at *Annals of Oncology* online). Seventeen cohorts provided data on the relative incidence of other cancers than cervical cancer^5^^,^^8^^,^^36^, ^37^, ^38^, ^39^, ^40^^,^^44^^,^^46^^,^^47^^,^^50^, ^51^, ^52^^,^^54^, ^55^, ^56^, ^57^ Ten cohorts reported on relative HPV-related non-cervical female anogenital cancer incidence [six on vaginal, seven on anal, seven on vulvar, and four on cervical plus vaginal (not separately) cancer relative incidence].^5^^,^^8^^,^^36^^,^^37^^,^^40^^,^^44^^,^^46^^,^^47^^,^^50^^,^^52^^,^^56^ Nine cohorts reported on relative non-HPV-related cancer incidence (five on endometrial, five on ovarian, five on breast, five on lung, and five on colorectal cancer relative incidence).^39^^,^^46^^,^^50^, ^51^, ^52^^,^^54^, ^55^, ^56^, ^57^ One cohort^4^ reported on relative cervical cancer mortality, and two^5^^,^^47^ on relative cervico-vaginal cancer mortality after treatment of CIN.

### Risk of bias assessment

The risk of bias assessment of the included studies is presented in supplementary Table S3, available at *Annals of Oncology* online. Only two publications scored a high risk of bias in one or more domains.^38^^,^^47^ The risk of selection bias was deemed to be moderate in five studies^4^^,^^38^^,^^39^^,^^43^^,^^46^ that did not have histological confirmation of CIN for the whole cohort. The risk of attrition bias was overall deemed to be low, as all studies used centralised registries. The risk of bias on prognostic factor measurement (i.e. treatment of CIN) was moderate in fourteen cohorts,^5^^,^^8^^,^^36^, ^37^, ^38^^,^^40^, ^41^, ^42^^,^^44^^,^^51^^,^^53^, ^54^, ^55^, ^56^, ^57^ as these may have included women with untreated CIN grade 2 (CIN2)^37^^,^^40^^,^^42^^,^^53^ or women treated with hysterectomy.^5^^,^^8^^,^^36^, ^37^, ^38^^,^^40^, ^41^, ^42^^,^^44^^,^^51^^,^^54^, ^55^, ^56^, ^57^ The lack of lag period between treatment and the start of follow-up introduced a moderate risk for outcome measurement bias in six studies.^39^^,^^41^^,^^43^^,^^51^^,^^52^^,^^55^ Lack of adjustment for age or calendar period introduced a moderate risk of bias in two studies^48^^,^^53^ and was unclear in another two.^43^^,^^45^ Statistical analysis and reporting did not lead to an increase in the risk of bias apart from two studies, due to selected grouping of treatment modalities^47^ or a case-control design.^38^

### Cervical cancer

**Figure 2:**
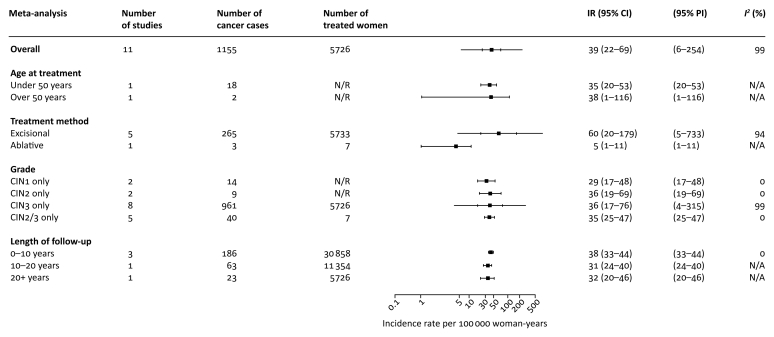
Pooled incidence rate of cervical cancer per 100 000 woman-years. Subgroup analyses according to age at cervical intraepithelial neoplasia (CIN) treatment, treatment method for CIN, CIN grade, and length of follow-up. CI, confidence interval; IR, incidence rate; N/A, not available (i.e. meta-analysis not possible); PI, prediction interval.

The pooled absolute IR of cervical cancer after treatment of CIN per 100 000 woman-years was reported in 11 cohorts (IR 39, 95% CI 22–69; *I*^2^ 99%; 11 cohorts, 1155 cancers, 5 562 889 woman-years) (Figure 2; supplementary Table S4 and supplementary Figure S1, available at *Annals of Oncology* online).^7^^,^^8^^,^^35^^,^^41^, ^42^, ^43^^,^^47^, ^48^, ^49^^,^^51^, ^52^, ^53^ In the subgroup analyses, the IR for older women (≥50 years) was 38 (1–116) and for younger (<50 years) was 35 (0.020–53). The rate increased as the grade of treated CIN increased (CIN1: 29, 17–48; CIN2: 36, 19–69; CIN3: 36, 17–76), and was the highest during the first decade (<10 years: 38, 33–44; 10–20 years: 31, 24–40; >20 years: 32, 20–46). Five cohorts reported on IR after excisional treatment (IR 60, 20–179, *I*^2^ 99%; five cohorts, 265 cancers, 797 848 woman-years).^35^^,^^43^^,^^45^, ^46^, ^47^ One cohort assessed IR after ablative treatments^46^ and one after cryotherapy^7^^,^^49^; meta-analysis was not possible.

**Figure 3:**
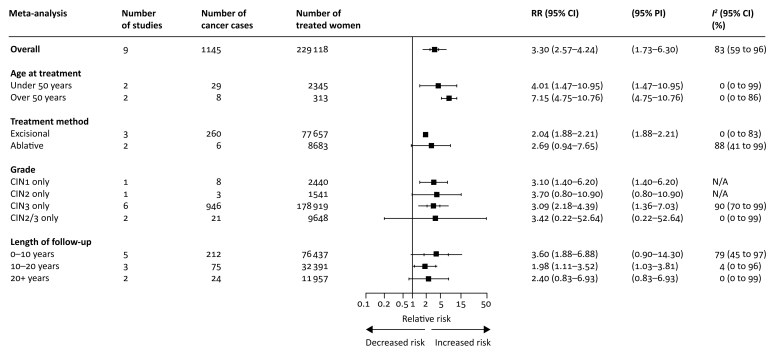
Pooled relative incidence of cervical cancer as compared with the reference population. Subgroup analyses according to age at cervical intraepithelial neoplasia (CIN) treatment, treatment method for CIN, CIN grade and length of follow-up. CI, confidence interval; N/A, not available (i.e. meta-analysis not possible); PI, prediction interval; RR, relative risk. *Fixed effect estimate.

The risk for cervical cancer after any local treatment of any grade of CIN was found to be higher in treated women rather than the reference population (RR 3.30, 2.57–4.24, *I*^2^ 83%; nine cohorts, 1145 cancers, 229 118 treated women) (Figure 3; supplementary Table S5 and supplementary Figure S2, available at *Annals of Oncology* online).^7^^,^^8^^,^^35^^,^^41^^,^^42^^,^^48^^,^^49^^,^^51^, ^52^, ^53^ The RR for women over the age of 50 was 7.15, 4.75–10.76, *I*^2^ 0%; two cohorts, eight cancers, 313 treated women. For women under 50 at the time of diagnosis, RR was 4.01, 1.47–10.95, *I*^2^ 0%; two cohorts, 29 cancers, 2345 treated women. The RR for treated CIN3 lesions alone was 3.09, 2.18–4.39, *I*^2^ 90%; six cohorts, 946 cancers, 178 919 treated women; meta-analytical pooling for other grades was not possible. The RR for cervical cancer was high after excisional treatment (RR 2.04, 1.88–2.21, *I*^2^ 0%; three cohorts, 260 cancers, 77 657 treated women). There was much uncertainty in the random-effects meta-analysis of RR after ablative treatment because of only two studies being included with non-overlapping CIs. A fixed-effect meta-analysis estimated the RR 2.69 (0.94–7.65) for ablation (supplementary Figure S2, available at *Annals of Oncology* online). The summary estimates for cervical RRs were highest in the early follow-up period, but remained consistently elevated thereafter. Our estimate for RR after 20 years was more uncertain, due to the small sample size (RR 2.40, 0.83–6.93, *I*^2^ 0%). However, the inverse variance method (random- or fixed-effect) reduced uncertainty (RR 2.40, 1.60–3.60) (supplementary Figure S2, available at *Annals of Oncology* online).

### Anogenital HPV-related cancers

**Figure 4:**
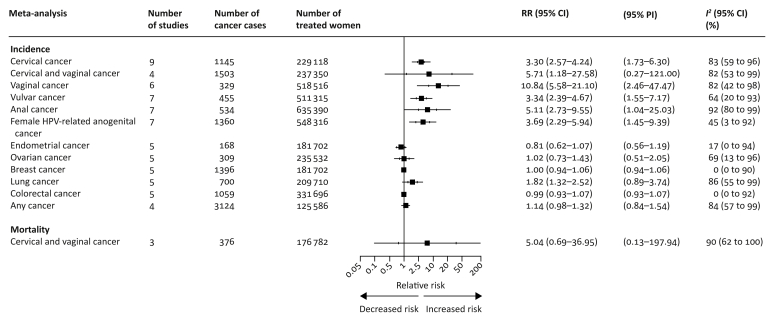
Pooled relative incidence of cervical and other cancers and mortality from cervical and vaginal cancer after treatment of cervical intraepithelial neoplasia (CIN) as compared with the reference population. CI, confidence interval; N/A, not available (i.e. meta-analysis not possible); PI, prediction interval; RR, relative risk.

Eleven cohorts were included in the meta-analysis of the RRs of anogenital HPV-related cancers (Figure 4; supplementary Table S6 and supplementary Figure S3, available at *Annals of Oncology* online).^5^^,^^36^, ^37^, ^38^^,^^40^^,^^44^^,^^46^^,^^47^^,^^50^^,^^52^^,^^56^ The RR of vaginal cancer was elevated in women treated for CIN (RR 10.84, 5.58–21.10, *I*^2^ 82%; six cohorts, 329 cancers, 518 516 treated women) and likewise for vulvar cancer (RR 3.34, 2.39–4.67, *I*^2^ 64%; seven cohorts, 455 cancers, 511 315 treated women), and anal cancer (RR 5.11, 2.73–9.55, *I*^2^ 92%; seven cohorts, 534 cancers, 635 390 treated women). The RR of cervical or vaginal cancers combined were elevated compared with the general population (RR 5.71, 1.18–27.58, *I*^2^ 82%; four cohorts, 1503 cancers, 237 350 treated women). Likewise, for the risk of any anogenital HPV-related cancer (RR 3.69, 2.29–5.94; *I*^2^ 45%; seven cohorts, 1360 cancers, 548 316 treated women).

### Non-HPV-related cancers

Nine cohorts reported on the RR of non-HPV-related cancers (Figure 4; supplementary Table S6 and supplementary Figure S3, available at *Annals of Oncology* online).^39^^,^^46^^,^^50^, ^51^, ^52^^,^^54^, ^55^, ^56^, ^57^ The risk of any cancer after CIN treatment was slightly elevated compared with the general population (RR 1.14, 0.98–1.32, *I*^2^ 84%; four cohorts, 3124 cancers, 125 586 treated women). By a different statistical technique (random-effects inverse variance model) we obtained narrower CIs (any cancer: RR 1.14, 1.04–1.25) (supplementary Figure S3, available at *Annals of Oncology* online). The only malignancy for which we had strong evidence that it had higher risk amongst the treated was lung cancer (RR 1.82, 1.32–2.52, *I*^2^ 86%; five cohorts, 700 cancers, 209 710 treated women).

### Mortality

Three cohorts were included in the meta-analysis of the mortality from cervical and/or vaginal cancer after CIN treatment (Figure 4; supplementary Table S6 and supplementary Figure S3, available at *Annals of Oncology* online).^4^^,^^5^^,^^47^ We found that mortality from cervical/vaginal cancer was elevated compared with untreated women, but our estimate was uncertain (RR 5.04, 0.69–36.94, *I*^2^ 90%; three cohorts, 376 deaths, 176 782 treated women). Using the random-effects inverse variance model, we obtained narrower CIs (RR 5.04, 2.04–12.49) (supplementary Figure S3, available at *Annals of Oncology* online). Meta-analysis of mortality from only cervical cancer, other cancers, or any cause was not possible because of the inadequate number of studies.

### Subgroup and sensitivity analyses

The between-study heterogeneity of the absolute IR of cervical cancer was reduced when subgroup analyses were carried out according to CIN grade and length of follow-up. In the subgroup analysis after treatment of CIN3 alone, sensitivity analyses according to geography reduced heterogeneity (supplementary Table S4, available at *Annals of Oncology* online).

For the RR of cervical cancer, heterogeneity was reduced in subgroup analyses according to age and method of treatment (supplementary Table S5, available at *Annals of Oncology* online).

Including only European studies reduced heterogeneity for anal, ovarian, cervical/vaginal, and any female HPV-related anogenital cancer. For vaginal and vulvar cancer, heterogeneity was still high for European countries, but choosing studies only from Northern or Western Europe reduced *I*^2^. For lung cancer, sensitivity analyses could not explain the high heterogeneity (supplementary Table S6, available at *Annals of Oncology* online). The effect estimates did not markedly change in the sensitivity analyses, apart from cervical/vaginal and anal cancer, where including only European studies at low risk of bias and studies only from Northern Europe, respectively, reduced the point estimates.

In order to confirm that selection of SIR or RR/HR did not affect point estimates or heterogeneity, we meta-analysed studies with HR/RR and SIR separately, and found no marked differences in the point estimates or heterogeneity. In two outcomes (anal and any HPV-related female anogenital cancer) sensitivity analysis of RR/HR reduced heterogeneity, but this could be explained by inclusion of only European studies for these two sensitivity analyses.

## Discussion

### Main findings in the context of current literature

Although the cancer-preventive effect of local CIN treatment is as high as 95%–99%,^7^^,^^48^ women after treatment are thought to be at higher risk of cervical disease than the general population. Our analysis estimated the pooled absolute IR for cervical cancer to be 39/100 000 woman-years, which is consistent with the only previously published pooled analysis (56/100 000 woman-years).^14^ Our pooled rate was slightly lower and likely more accurate, as we have included only studies with centralised follow-up, eliminating overestimates that may result from small single-arm studies pooled in the previous report.^14^

Our findings show that the pooled cervical cancer RR amongst treated women was three times higher than the general population. This risk remains elevated for at least 20 years after the index treatment. These results were also in agreement with a previously published report.^14^

The RR of other HPV-related anogenital cancers was also markedly raised in treated as opposed to untreated women. The risk of non-HPV-related malignancies was not increased when compared with the general population, with the exception of a twofold rise in lung cancer, possibly reflecting a significantly higher prevalence of smokers amongst women treated for CIN, given that smoking is a known risk factor for CIN.^64^ We found that mortality was five times higher than that in the general population, although there was uncertainty around this estimate.

In recent decades, there has been a transition from more radical excision with cold knife conisation (CKC) that was routinely practiced in the 1980s to laser conisation, and to the less aggressive large loop excision of the transformation zone (LLETZ) in the 1990s that is practiced predominantly to date.^8^^,^^65^ The subsequent increased awareness that treatment, particularly excisional, increases the risk of preterm birth and other adverse reproductive outcomes in subsequent pregnancies^66^, ^67^, ^68^, ^69^, ^70^, ^71^ has led to further reduction in the radicality of treatment, with more clinicians opting for smaller excision,^72^ or even ablative treatment.^66^ The previously published Cochrane review exploring the comparative efficacy of excision versus ablation was grossly underpowered to show a difference for highly efficacious treatment; this would require a large, appropriately-powered non-inferiority trial that has yet to be conducted.^73^ A recent meta-analysis provided indirect evidence that LLETZ when compared with CKC, and incomplete as compared with complete margin clearance, affects treatment failure rates (7 versus 2% and 17 versus 4%, respectively).^3^ The impact of less radical treatments on the future risk of invasion remains unclear.^5^^,^^74^ Although we carried out analyses for excisional and ablative techniques separately, these were not informative and were limited by the small number of studies. In one study, the point estimate for cervical cancer was higher after ablative than excisional treatment.^46^ In two cohorts exploring the differences between treatment methods, cryotherapy was shown to increase cervical cancer risk threefold when compared with other local methods^48^ or CKC.^49^ The pooled RRs after excisional or ablative treatment were elevated compared with the reference population, although based on just a few studies and small numbers of incident cancers.

There are a number of plausible theories explaining the increase in the risk of cervical cancer after CIN treatment. A number of cases predominantly diagnosed close to the index treatment may be a result of inadequate disease excision, disease hidden in the endocervical crypts, and misdiagnosis of invasive malignancies as preinvasive. To minimise the risk of inflating the pooled cancer incidence due to misdiagnosis, all but one study used a lag-period of at least 6 months from the time of treatment to capture faults in diagnosis. Despite this, the incidence of cervical cancer was comparatively higher in the early follow-up periods, although this continued to be higher than the general population for more than two decades. Residual preinvasive disease within or outside of the endocervical crypts is harder to detect and prevent after previous treatment, as cytology and colposcopy can be more difficult to perform adequately and interpret.^41^ Avoiding heavy cauterisation of the crater during treatment might decrease the risk of ‘burying’ residual disease inside the crypts, which subsequent cytology and colposcopy might not be able to detect.

‘Lingering’ disease, persistent high-risk HPV infection, and misdiagnosis could only partly explain the prolonged increase in the risk of invasive cancer after CIN treatment in some cases. The higher risk of all other HPV-related malignancies and the slightly higher risk in women over the age of 50 years suggest further possible explanations. Although cervical cancer is not considered to be a hereditary disease, there is evidence to suggest that genetic polymorphisms,^75^ variations in immune defences and an innate immune system,^76^ microbiome predisposition^77^ and an inherent sensitivity to HPV infection and persistence in some individuals may increase their risk of HPV-related malignancies. These women are often particularly sensitive to the infection and rapidly get re-infected even if they clear this at the time of treatment.

This analysis may inform more personalised screening strategies in women previously treated for CIN and assist decision-making for clinicians and health policy makers. The interruption of cervical screening for previously treated women at an age similar to that of the general population has been long debated.^78^ Advocates of prolonged screening for the subset of treated women note that the second peak in cervical cancer incidence, as well as peak incidence of other HPV-related cancers, is observed after the end of screening,^79^ whereas cervical cancer mortality increases with advancing age.^80^ Our findings support this notion, as the risk remained high for more than 20 years after treatment and was slightly higher for women over 50 years old. Prolonged screening after treatment for 20 years, or even for the remainder of their lifetime, may enhance prevention of cervical cancer, but may also promote early detection of asymptomatic vulvar, vaginal, and other HPV-related neoplasms post-treatment, as these women will attend health services and have an examination of the anogenital area. Further recommendations on strategies for the prevention and early detection of non-cervical malignancies are limited by the absence of currently validated screening tools. Future research should further explore the value and cost-effectiveness of preventative interventions for other HPV-related malignancies (such as vault sample and/or colposcopic inspection in hysterectomised women for the prevention of vaginal and vulvar cancer, and anal sampling with anoscopy for anal cancer). With the introduction of the hrHPV DNA test in primary screening and HPV prophylactic vaccination, current screening programmes have undergone substantial reconfiguration making previously published evidence difficult to apply to awaited future screening structures. The expected prolongation of screening intervals may allow extension of current screening programmes beyond the age of 65 years, in line with prolonged life expectancy, particularly for treated women. These results also support further education for lifestyle, sexual, and behaviour changes that may enhance the prevention of HPV-related malignancies, and further emphasise the need for smoking cessation initiatives.

### Strengths and weaknesses

This is the first systematic review and meta-analysis of observational studies with centralised follow-up assessing cancer incidence and mortality after treatment of CIN. Centralised registry data offer great advantages in minimising losses to follow-up due to population movement, and attrition bias that can arise from women facing barriers to healthcare access or without symptoms prompting them to seek medical advice, when based on the records of a single clinic. The risk of bias in included studies was, overall, considered to be low. Furthermore, we used the Knapp-Hartung-Sidik-Jonkman method for our analyses, which is known to outperform the traditional Wald type method, particularly in the context of a limited number of studies. This method usually produces more conservative estimates, reduces the risk of spurious results, and is robust to the use of different estimators for the between-study variance.

There were several limitations in our meta-analyses. In some of these analyses, there was high between-study heterogeneity, resulting in uncertainty in the estimated effects. Subgroup and sensitivity analyses according to age, CIN grade, treatment method, or length of follow-up were able to reduce heterogeneity to some extent, although many included only a small number of studies. Furthermore, we could not reliably assess for small-study effects or publication bias due to the dearth of studies. Finally, we were unable to perform subgroup analyses for the status of post-treatment test-of-cure (HPV testing and/or cytology at 6 months) due to limitations in the published data. Future studies should stratify cancer rates to HPV status after treatment.

### Conclusions

Women treated for CIN have an increased incidence of not only cervical, but of all HPV-related female genital tract cancers, compared with the general population. Treated women remain at increased risk for developing invasive cervical cancer for more than 20 years. Our findings suggest that a sufficiently long follow-up, perhaps lifelong, after the end of organised screening may be warranted for this high-risk population previously treated for CIN.

## Supplementary data

Supplementary Methods and Results
