# Supplementary material for: Incidence and mortality from cervical cancer and other malignancies after treatment of cervical intraepithelial neoplasia: a systematic review and meta-analysis of the literature
Source: Ann Oncol. 2020 Feb;31(2):213–27. doi: 10.1016/j.annonc.2019.11.004 (PMC7479506; doi:10.1016/j.annonc.2019.11.004)
Supplement: Supplementary Methods and Results [file mmc1.docx]

**SUPPLEMENTARY METHODS**

**Search Strategies** (date:18.8.2018)

We used a very broad search with keywords including ‘cervical intraepithelial neoplasia (CIN)’, ‘cervical cancer’, ‘LLETZ or LEEP’, ‘conisation’, ‘excision’, ‘follow-up’. To identify studies that might have been missed during the electronic search, we further hand-searched the references of the articles in the full-text stage. Titles, abstracts, and full texts were screened in duplicate and disagreements were resolved with discussion.

**Pubmed**

| 1. (CIN or Cervical intraepithelial neoplasia or Cervical neoplasm or cervical dysplasia).ab,ot,ti. |  |
| --- | --- |

| 2. (Therapy or treatment or Conisation or large loop excision of the transformation Zone or LLETZ or loop electrosurgical excision procedure or LEEP or cryo or ablat* or hysterectomy or NETZ).ab,ot,ti. |  |
| --- | --- |

| 3. (Cervical cancer or Cancer or Survival or Mortality or Outcomes).ab,ot,ti. |  |
| --- | --- |

| 4. 1 and 2 and 3 |
| --- |
| 5. limit 4 to human |

**Embase**

| 1. (CIN or Cervical intraepithelial neoplasia or Cervical neoplasm or cervical dysplasia).ab,ot,ti. |  |
| --- | --- |

| 2. (Therapy or treatment or conisation or conization or large loop excision of the transformation Zone or LLETZ or loop electrosurgical excision procedure or LEEP or cryo* or ablat* or hysterectomy, or NETZ).ab,ot,ti. |  |
| --- | --- |

| 3. (Cervical cancer or Cancer or Survival or Mortality or Outcomes).ab,ot,ti. |  |
| --- | --- |

| 4. 1 and 2 and 3 |  |
| --- | --- |

| 5. limit 4 to human |
| --- |

**CENTRAL**

| 1. (CIN or Cervical intraepithelial neoplasia or Cervical neoplasm or cervical dysplasia) |  |
| --- | --- |

| 2. (Therapy or treatment or conisation or conization or large loop excision of the transformation Zone or LLETZ or loop electrosurgical excision procedure or LEEP or cryo or ablat or hysterectomy, or NETZ). |  |
| --- | --- |

| 3. (Cervical cancer or Cancer or Survival or Mortality or Outcomes) |  |
| --- | --- |

in Trials, Methods Studies and Technology Assessments

**Data synthesis**

We estimated the summary cancer incidence or mortality RR and its 95% CI using the Hartung-Knapp-Sidik-Jonkman method[1, 2] to handle meta-analyses with small number of studies. When four or fewer studies were included in a meta-analysis, the modified Hartung-Knapp-Sidik-Jonkman method was performed[3]. For fewer than five studies we also used the inverse variance method to assess robustness of results (presented in Supplementary Figures). We used the random-effects model, although incorporation of between-study variance could result in power loss, particularly in meta-analyses with a small number of studies[4]. For this reason, we also performed a fixed-effect meta-analysis for outcomes with four or less studies (presented in Supplementary Figures). In meta-analyses where random-effects meta-analysis yielded too wide (and thus uninformative) confidence intervals, only results from fixed-effect meta-analysis are presented in Figures and Supplementary Tables.

When study-specific RR estimates were not directly available, we estimated incidence rate ratios and their 95% CI from the number of incident cases and person-years for exposed and unexposed women using the *epitab* Stata command[5]. I^2^ statistics was calculated for both absolute incidence rate (IR) and relative cervical cancer incidence. However, 95% confidence interval (CI) was only possible to be calculated for relative cervical cancer incidence; *metafor* package does not provide CI for the I^2^ under the generalised linear mixed-effects model which was used for the calculation of the IR.

**Risk of Bias Assessment**

The quality of the included studies was assessed independently in duplicate by using the Quality in Prognosis Studies (QUIPS) tool. Disagreements were resolved through discussion. QUIPS explores the quality of studies with four grades (unclear, low, moderate, or high risk of bias) in six domains: study participation, study attrition, prognostic factor measurement, outcome measurement, study confounding, and statistical analysis and reporting. The overall risk of bias was determined by the domain with the worst score.

A) Study participation: Studies using histological diagnosis of CIN in all cases were at low risk of bias. Those that did not describe whether diagnosis of CIN was histological or cytological, were downgraded to moderate risk of bias, unless the percentage of cytological diagnoses was less than 10%.

B) Study attrition: The risk of attrition bias was low if the follow-up was based on nation- or region-wide registers. This was moderate if a study used data from both registries and hospital records.

C) Prognostic factor measurement: Studies clearly documenting that all women were treated locally were at low risk of bias. If hysterectomies could have been included, the study was at moderate risk unless the rate was less than 10%. If the study did not document that all lesions were treated, the risk of bias was also moderate due to selective conservative management of small number of CIN2 lesions. If the study only included CIN3, the study was not downgraded as conservative management is expected to be very low.

D) Outcome measurement: Studies with no lag period were downgraded to moderate risk of bias.

E) Study adjustment: If the reference population was not matched or adjusted for age or calendar period, the study was downgraded to moderate risk. If a study did not use a reference population, the risk was considered unclear.

F) Statistical analysis and reporting: Studies with a case-control design or with suspicion of selective reporting were downgraded from low to high risk of bias.

**Subgroup Analyses**

For cervical cancer absolute and relative incidence, we performed subgroup analyses according to: the age at CIN treatment (over or under age 50); the treatment method (excisional: cold knife conisation [CKC]; laser conisation (LC); needle excision of the transformation zone [NETZ]; large loop excision of the transformation zone [LLETZ / LEEP] – ablative: laser ablation [LA]; cold coagulation [CC]; cryotherapy [CT]); the grade of the initial histology (CIN1, 2, 3) and the length of follow-up time (0-10, 10-20, >20, 0-5, 5-10, 10-15, 15-20, 0-20 years).

The follow-up windows and age groups varied across studies. When these data could not be directly grouped together for meta-analysis due to these variations, we first meta-analysed the data from different age or follow-up groups within a study, and subsequently included one common estimate with corresponding confidence interval per study in the main meta-analysis in question.

**Sensitivity analyses**

In order to explore the possible sources of heterogeneity and differences in summary estimates, whenever more than two studies were available we performed a predefined series of sensitivity analyses for absolute incidence of cervical cancer or relative incidence and mortality of cervical or other cancers: according to the choice of comparison group and the effect estimate used (SIR or other); according to continent, including Europe, Northern Europe (Finland, Sweden, Norway, Denmark), Western Europe (the Netherlands, UK, Switzerland); including only studies where none of the domains had high risk of bias; including only studies that used a lag-period between treatment and beginning of cancer incidence follow-up; including only studies that had no cytological diagnoses of CIN; including only studies that had no untreated women; including only studies that had no hysterectomies. For combined outcomes (HPV-related female anogenital cancer incidence and colorectal cancer incidence) we performed the analyses also excluding studies that did not report on all possible outcomes.

**SUPPLEMENTARY RESULTS**

**Eligible studies**

Thirteen cohorts reported on absolute[7, 8, 37, 43-45, 47-51, 53-55] and 10 cohorts on relative[7, 8, 37, 43, 44, 48, 50, 51, 53-55] cervical cancer incidence after CIN treatment. The treatment methods used were reported in eight cohorts[6, 8-13, 15, 17]. Four cohorts[6, 13, 15, 17, 19] excluded women treated primarily with hysterectomy. In one study, we included data only from 1981 onwards when the rate of hysterectomies as the primary treatment markedly reduced due to the introduction of local treatments[14]. Five cohorts provided relative risk data on cervical cancer incidence stratified according to the length of follow-up[6-8, 15, 17, 19] and two according to the age at treatment[7, 8]. All apart from three cohorts[9, 16, 18] used a ‘lag period’ of at least 6 months since the treatment. In most studies diagnosis of CIN was based on histopathology; in five studies this was not clearly stated[9, 11, 20-22] and in one study histology was available in 90% and cytology in 10% of cases[8]. Four studies may have included untreated CIN2 or worse lesions as the treatment was not clearly documented, although the number is likely to have been very small[8, 19, 23, 24].

Seventeen cohorts provided data on relative incidence of other cancers than cervical cancer[11, 12, 14, 16, 18, 20, 21, 23-32]. Ten cohorts reported on relative HPV-related non-cervical female anogenital cancer incidence (six on vaginal, seven on anal, seven on vulvar and four on cervical plus vaginal (not separately) cancer relative incidence)[11, 12, 14, 18, 23-27, 30, 32]. Nine cohorts reported on relative non-HPV-related cancer incidence (five on endometrial, five on ovarian, five on breast, five on lung and five on colorectal cancer relative incidence)[11, 16, 18, 21, 27-31]. One cohort[22] reported on relative cervical cancer mortality, and two[12, 32] on relative cervico-vaginal cancer mortality after treatment of CIN.

**References**

1. Hartung J, Knapp G. A refined method for the meta-analysis of controlled clinical trials with binary outcome. Stat Med 2001; 20: 3875-3889.

2. Sidik K, Jonkman JN. A simple confidence interval for meta-analysis. Stat Med 2002; 21: 3153-3159.

3. Knapp G, Hartung J. Improved tests for a random effects meta-regression with a single covariate. Stat Med 2003; 22: 2693-2710.

4. Jackson D, Turner R. Power analysis for random-effects meta-analysis. Res Synth Methods 2017; 8: 290-302.

5. StataCorp. Stata Statistical Software: Release 13. College Station, TX: StataCorp LP 2013.

6. Sand FL, Frederiksen K, Munk C et al. Long-term risk of cervical cancer following conization of cervical intraepithelial neoplasia grade 3-A Danish nationwide cohort study. International Journal of Cancer 2018; 142: 1759-1766.

7. Rebolj M, Helmerhorst T, Habbema D et al. Risk of cervical cancer after completed post-treatment follow-up of cervical intraepithelial neoplasia: population based cohort study. BMJ 2012; 345: e6855.

8. Rapiti E, Usel M, Neyroud-Caspar I et al. Omission of excisional therapy is associated with an increased risk of invasive cervical cancer after cervical intraepithelial neoplasia III. Eur J Cancer 2012; 48: 845-852.

9. Kreimer AR, Schiffman M, Herrero R et al. Long-term risk of recurrent cervical human papillomavirus infection and precancer and cancer following excisional treatment. Int J Cancer 2012; 131: 211-218.

10. Kocken M, Helmerhorst TJM, Berkhof J et al. Risk of recurrent high-grade cervical intraepithelial neoplasia after successful treatment: a long-term multi-cohort study. The Lancet Oncology 2011; 12: 441-450.

11. Jakobsson M, Pukkala E, Paavonen J et al. Cancer incidence among Finnish women with surgical treatment for cervical intraepithelial neoplasia, 1987-2006. Int J Cancer 2011; 128: 1187-1191.

12. McCredie MR, Paul C, Sharples KJ et al. Consequences in women of participating in a study of the natural history of cervical intraepithelial neoplasia 3. Aust N Z J Obstet Gynaecol 2010; 50: 363-370.

13. Melnikow J, McGahan C, Sawaya GF et al. Cervical Intraepithelial Neoplasia Outcomes After Treatment: Long-term Follow-up From the British Columbia Cohort Study. JNCI Journal of the National Cancer Institute 2009; 101: 721-728.

14. Strander B, Andersson-Ellstrom A, Milsom I, Sparen P. Long term risk of invasive cancer after treatment for cervical intraepithelial neoplasia grade 3: population based cohort study. Bmj 2007; 335: 1077.

15. Kalliala I, Nieminen P, Dyba T et al. Cancer free survival after CIN treatment: comparisons of treatment methods and histology. Gynecol Oncol 2007; 105: 228-233.

16. Taylor TH, Bringman D, Anton-Culver H. Malignancies following in situ cervical cancer in Hispanic Americans and non-Hispanic Whites. Gynecol Oncol 2006; 103: 1012-1016.

17. Kalliala I, Anttila A, Pukkala E, Nieminen P. Risk of cervical and other cancers after treatment of cervical intraepithelial neoplasia: retrospective cohort study. BMJ 2005; 331: 1183-1185.

18. Evans HS, Newnham A, Hodgson SV, Møller H. Second primary cancers after cervical intraepithelial neoplasia III and invasive cervical cancer in Southeast England. Gynecologic Oncology 2003; 90: 131-136.

19. Mitchell H, Hocking J. Influences on the risk of recurrent high grade cervical abnormality. International Journal of Gynaecological Cancer 2002; 12: 728-734.

20. Coffey K, Gaitskell K, Beral V et al. Past cervical intraepithelial neoplasia grade 3, obesity and earlier menopause are associated with an increased risk of vulval cancer in postmenopausal women. British Journal of Cancer 2016; 115: 599-606.

21. Kirkegård J, Farkas DK, Søgaard M et al. Conization as a marker of persistent cervical human papillomavirus (HPV) infection and risk of gastrointestinal cancer: a Danish 34-year nationwide cohort study. Cancer Causes & Control 2014; 25: 1677-1682.

22. Jakobsson M, Gissler M, Paavonen J, Tapper AM. Long-term mortality in women treated for cervical intraepithelial neoplasia. BJOG 2009; 116: 838-844.

23. Sand FL, Munk C, Jensen SM et al. Long-Term Risk for Noncervical Anogenital Cancer in Women with Previously Diagnosed High-Grade Cervical Intraepithelial Neoplasia: A Danish Nationwide Cohort Study. Cancer Epidemiology Biomarkers & Prevention 2016; 25: 1090-1097.

24. Gaudet M, Hamm J, Aquino-Parsons C. Incidence of ano-genital and head and neck malignancies in women with a previous diagnosis of cervical intraepithelial neoplasia. Gynecol Oncol 2014; 134: 523-526.

25. Ebisch R, Rutten D, IntHout J et al. Long-Lasting Increased Risk of Human Papillomavirus–Related Carcinomas and Premalignancies After Cervical Intraepithelial Neoplasia Grade 3: A Population-Based Cohort Study. Journal of Clinical Oncology 2017; 35: 2542-2550.

26. Saleem AM, Paulus JK, Shapter AP et al. Risk of Anal Cancer in a Cohort With Human Papillomavirus–Related Gynecologic Neoplasm. Obstetrics & Gynecology 2011; 117: 643-649.

27. Edgren G, Sparén P. Risk of anogenital cancer after diagnosis of cervical intraepithelial neoplasia: a prospective population-based study. The Lancet Oncology 2007; 8: 311-316.

28. Levi F, Randimbison L, La Vecchia C, Franceschi S. Incidence of invasive cancers following carcinoma in situ of the cervix. Br J Cancer 1996; 74: 1321-1323.

29. Frisch M, Melbye M. Risk of lung cancer in pre- and post-menopausal women with anogenital malignancies. International Journal of Cancer 1995; 62: 508-511.

30. Bjorge T, Hennig EM, Skare GB et al. Second primary cancers in patients with carcinoma in situ of the uterine cervix. The Norwegian experience 1970-1992. Int J Cancer 1995; 62: 29-33.

31. Pettersson F, Ryberg M, Malker B. Second primary cancer after treatment of invasive carcinoma of the uterine cervix, compared with those arising after treatment for in situ carcinomas. An effect of irradiation? A cancer registry study. Acta Obstet Gynecol Scand 1990; 69: 161-174.

32. Strander B, Hallgren J, Sparen P. Effect of ageing on cervical or vaginal cancer in Swedish women previously treated for cervical intraepithelial neoplasia grade 3: population based cohort study of long term incidence and mortality. BMJ 2014; 348: f7361.
