# Supplementary material for: Incidence and mortality from cervical cancer and other malignancies after treatment of cervical intraepithelial neoplasia: a systematic review and meta-analysis of the literature
Source: Ann Oncol. 2020 Feb;31(2):213–27. doi: 10.1016/j.annonc.2019.11.004 (PMC7479506; doi:10.1016/j.annonc.2019.11.004)

**Supplementary Figures 1**: Forest plots for incidence rate of cervical cancer (per 100,000 woman-years): overall incidence and subgroup analyses according to age at CIN treatment, treatment method for CIN, CIN grade and length of follow-up (when ≥2 studies are available).

**Overall**


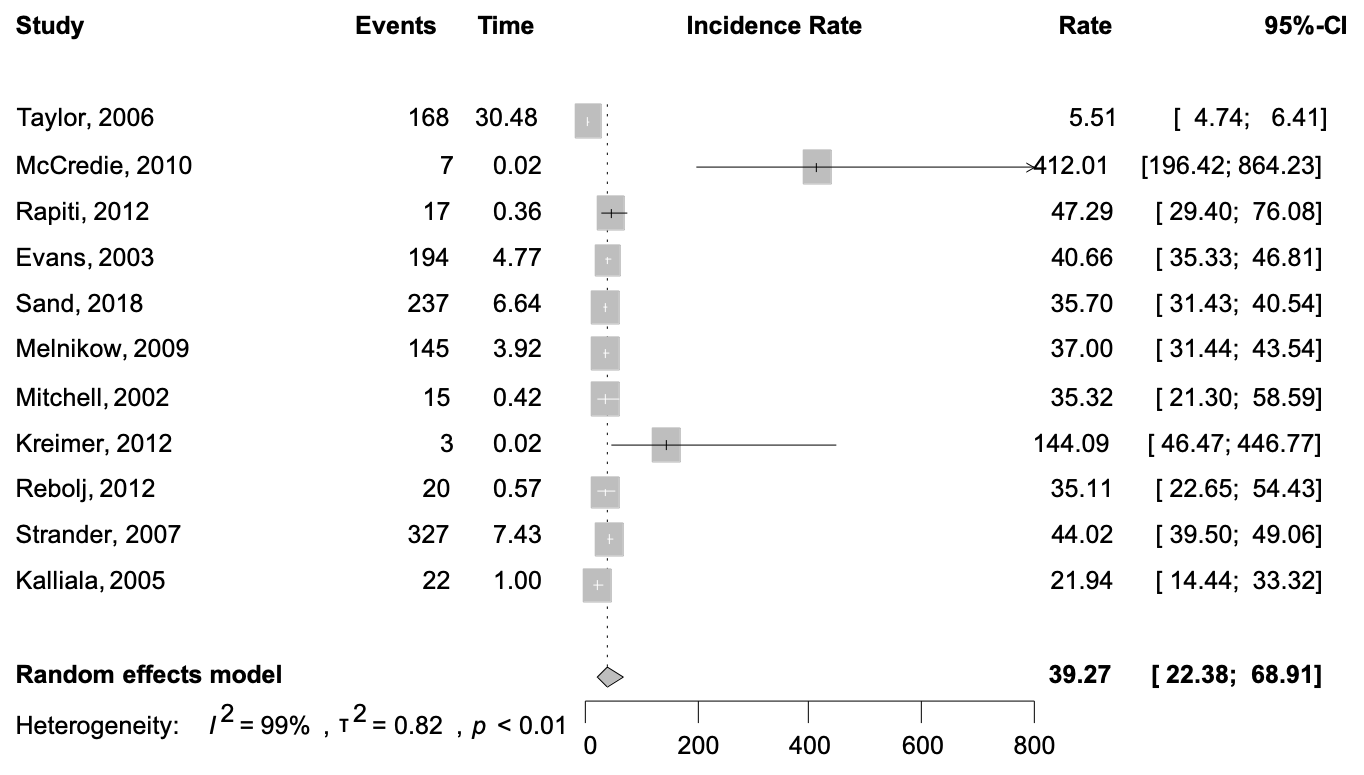


**Treatment method for CIN: excision**


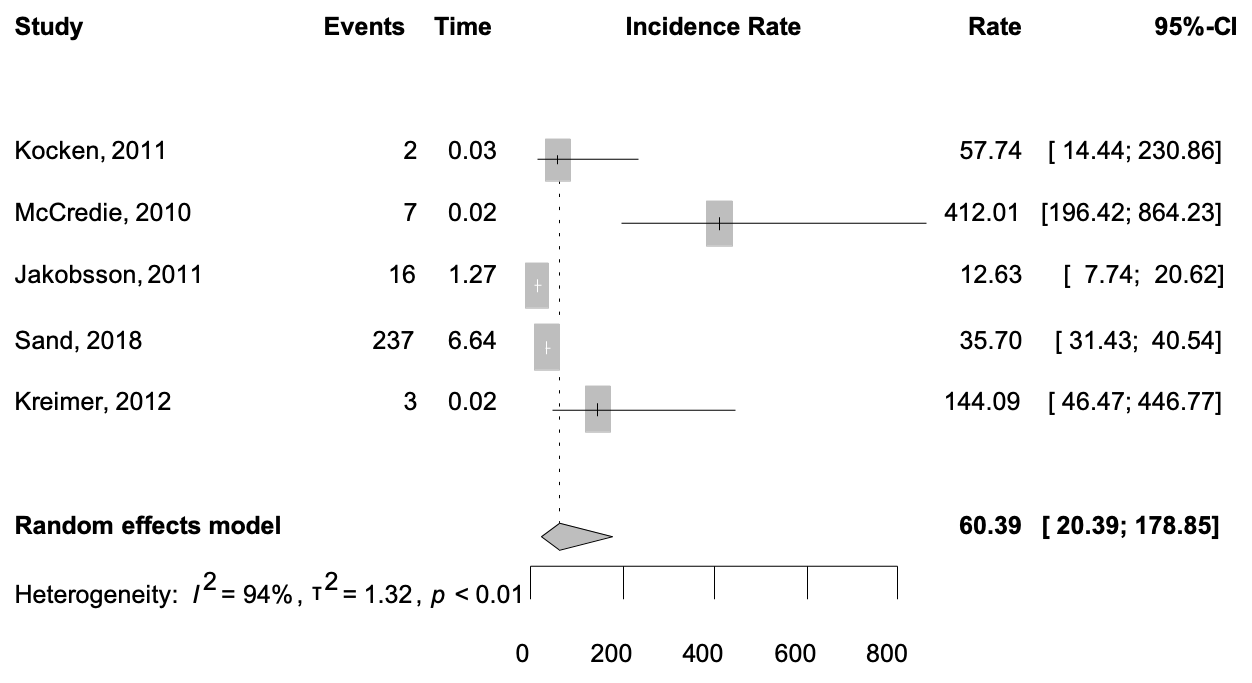


**CIN grade: CIN1**

**
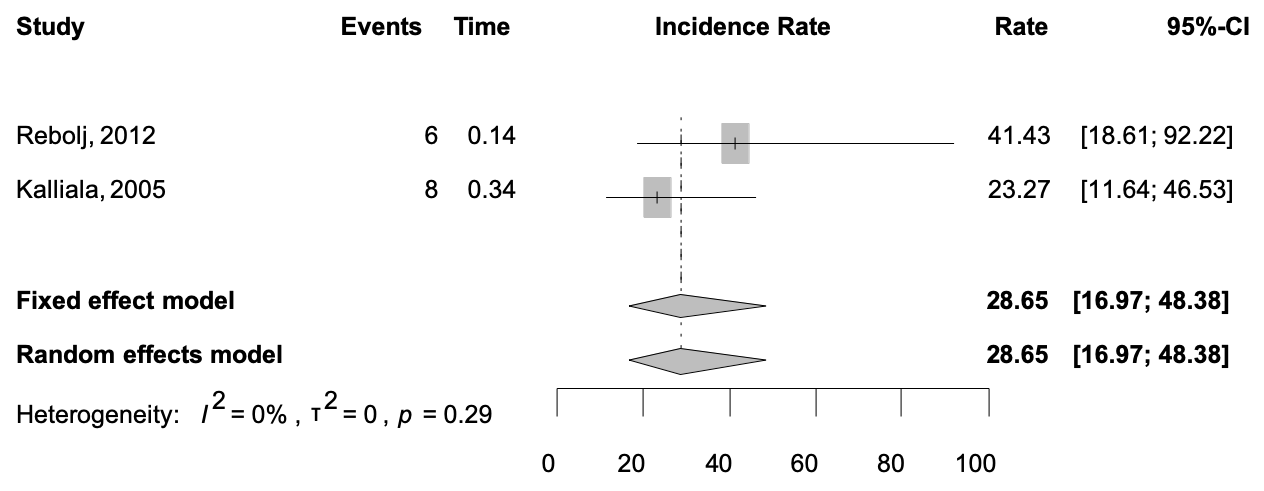
**

**CIN grade: CIN2**


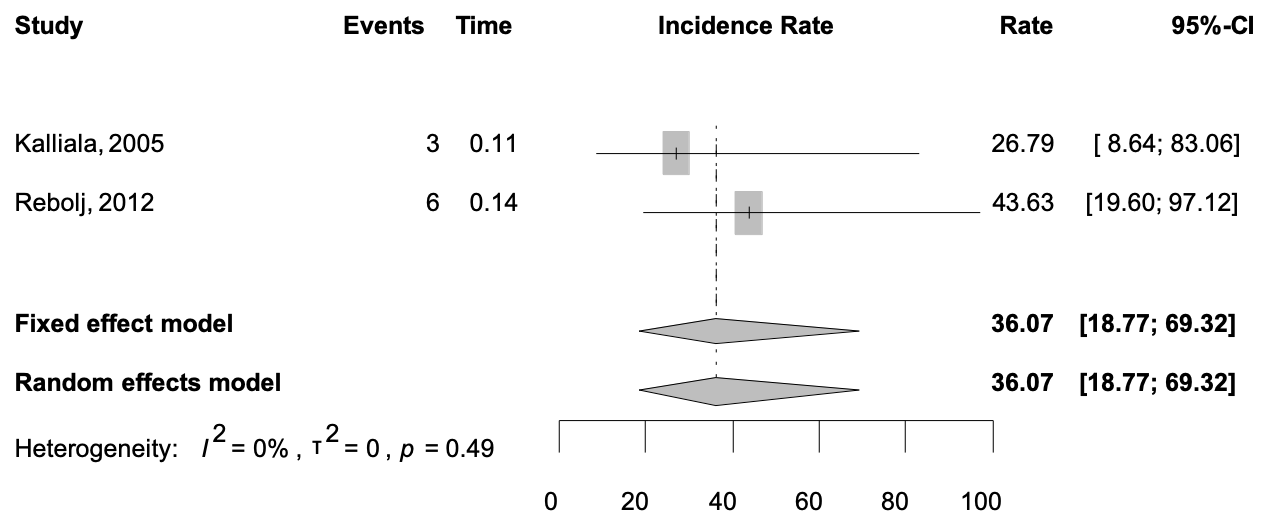


**CIN grade: CIN3**


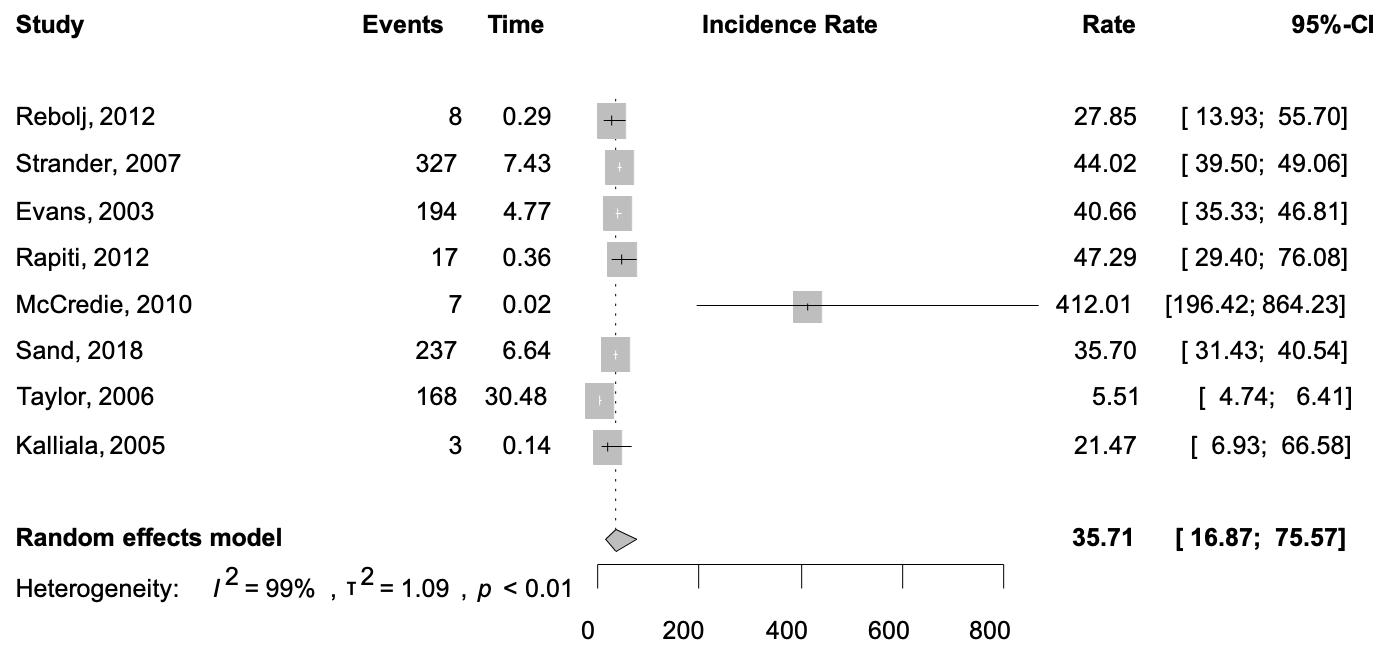


**CIN grade: CIN2/3**


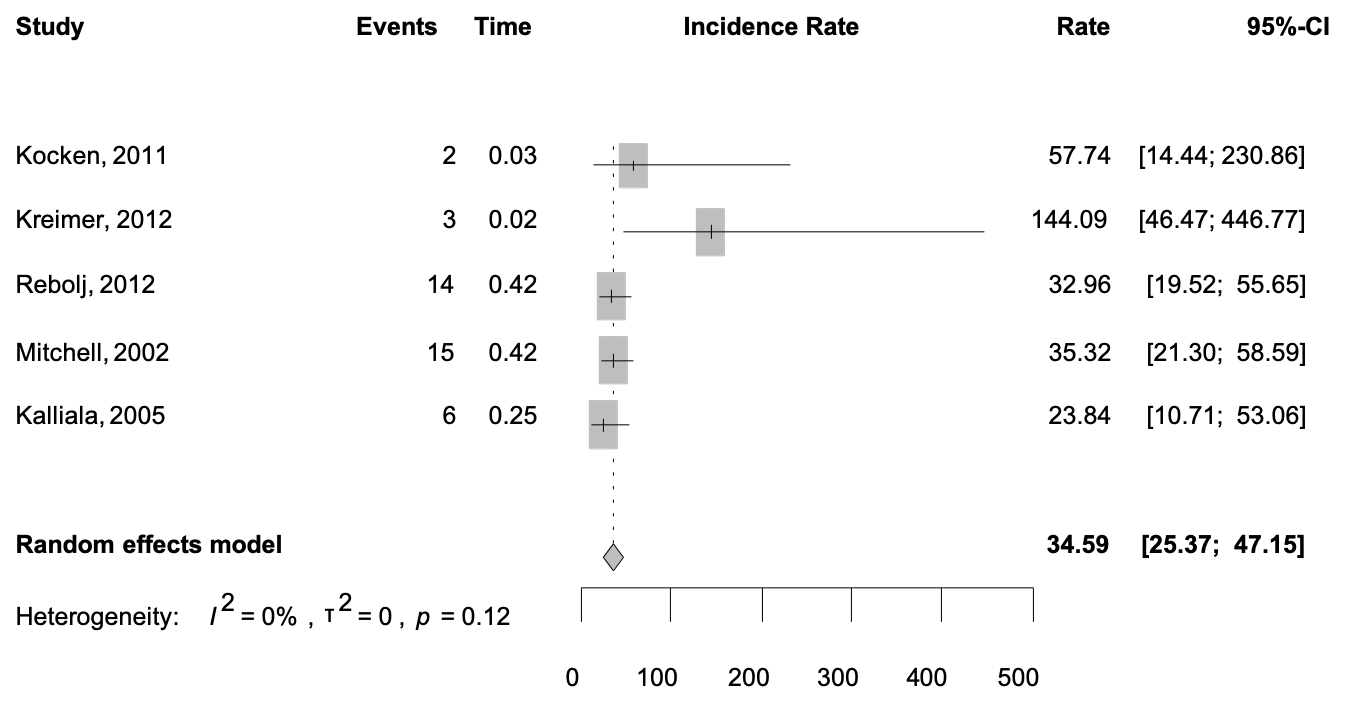


**Length of follow-up after CIN treatment: 0-10y**


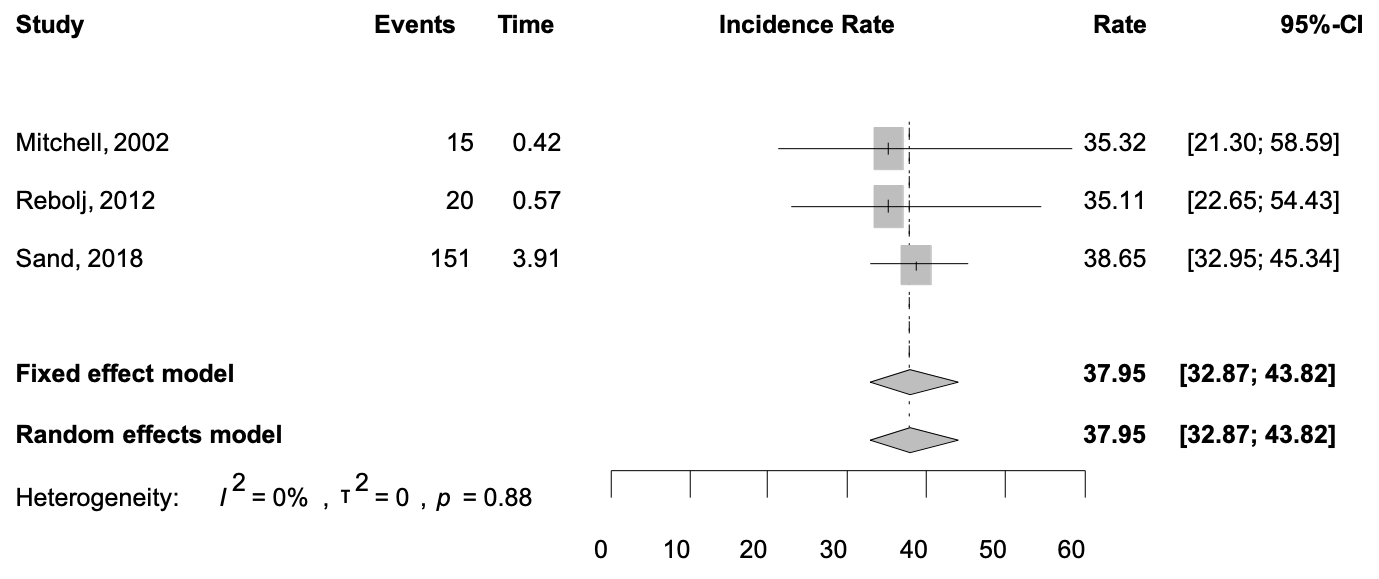


**Length of follow-up after CIN treatment: 0-5y**


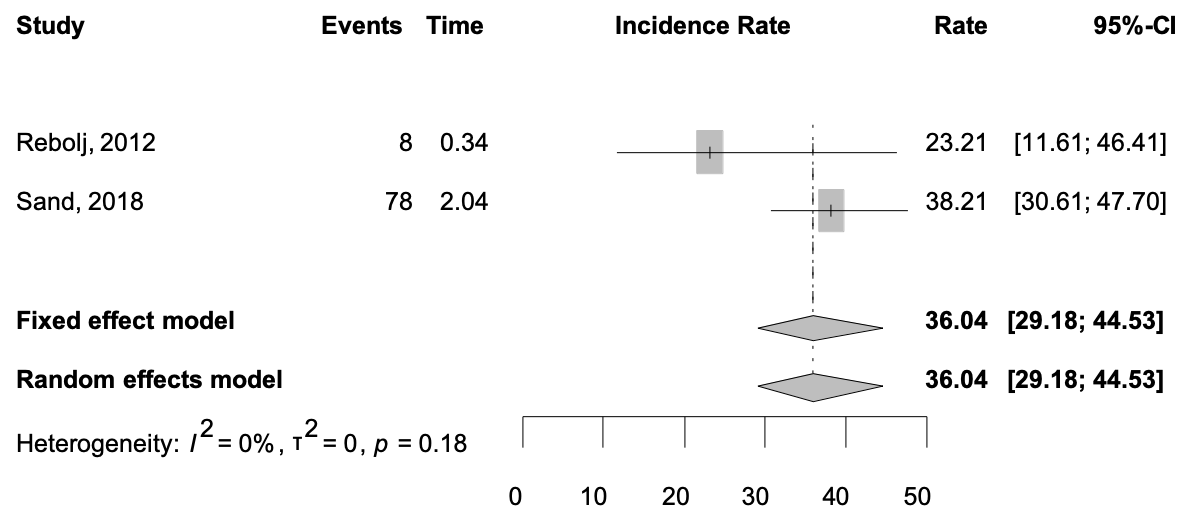


**Length of follow-up after CIN treatment: 5-10y**


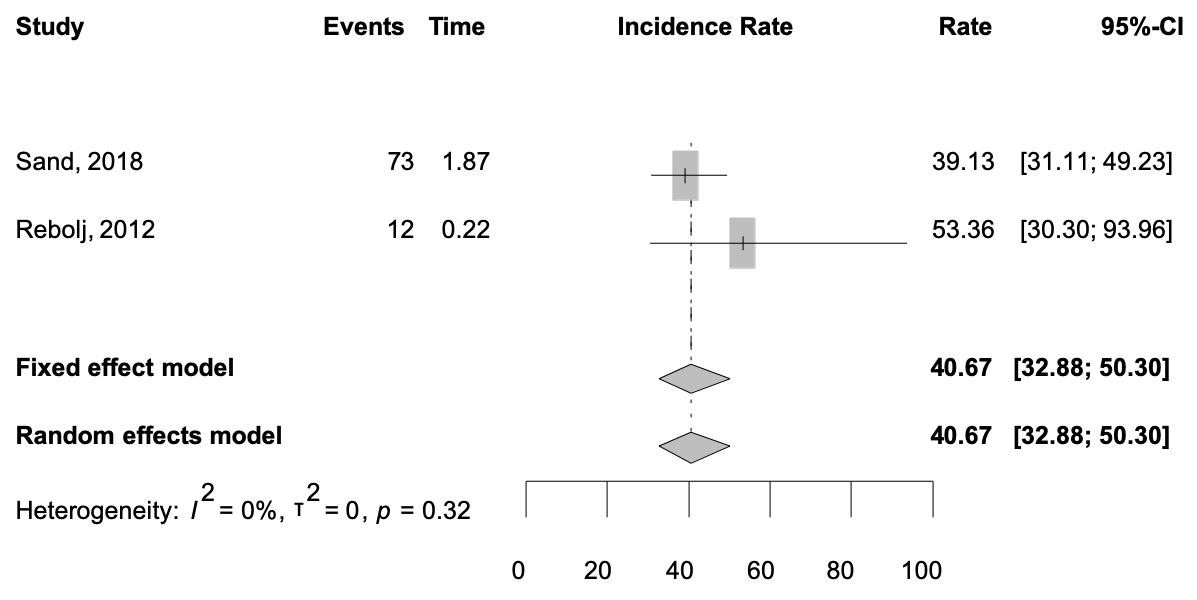


**Length of follow-up after CIN treatment: 0-20y**


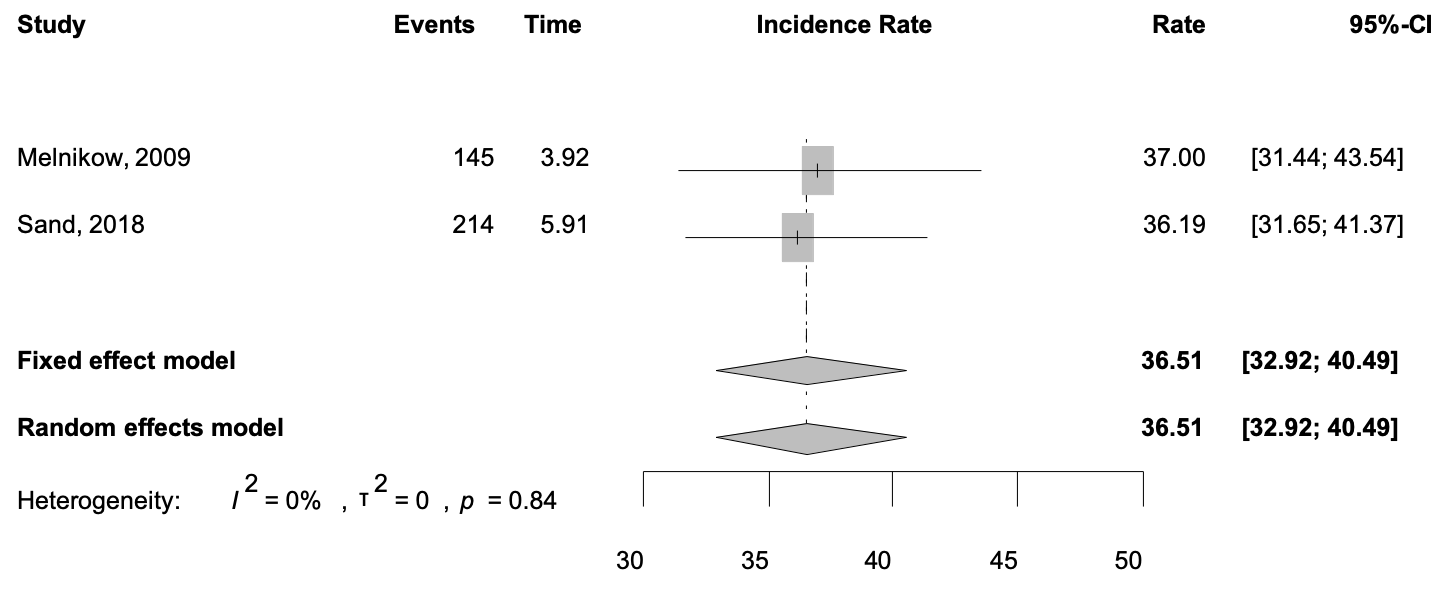

Supplement: Supplementary Figure S1 [file mmc2.docx]
