# Supplementary material for: Incidence and mortality from cervical cancer and other malignancies after treatment of cervical intraepithelial neoplasia: a systematic review and meta-analysis of the literature
Source: Ann Oncol. 2020 Feb;31(2):213–27. doi: 10.1016/j.annonc.2019.11.004 (PMC7479506; doi:10.1016/j.annonc.2019.11.004)

**Supplementary Figures 2**: Pooled relative incidence of cervical cancer as compared to the reference population: overall incidence and subgroup analyses according to age at CIN treatment, treatment method for CIN, CIN grade and length of follow-up (when ≥2 studies are available).

**Overall**


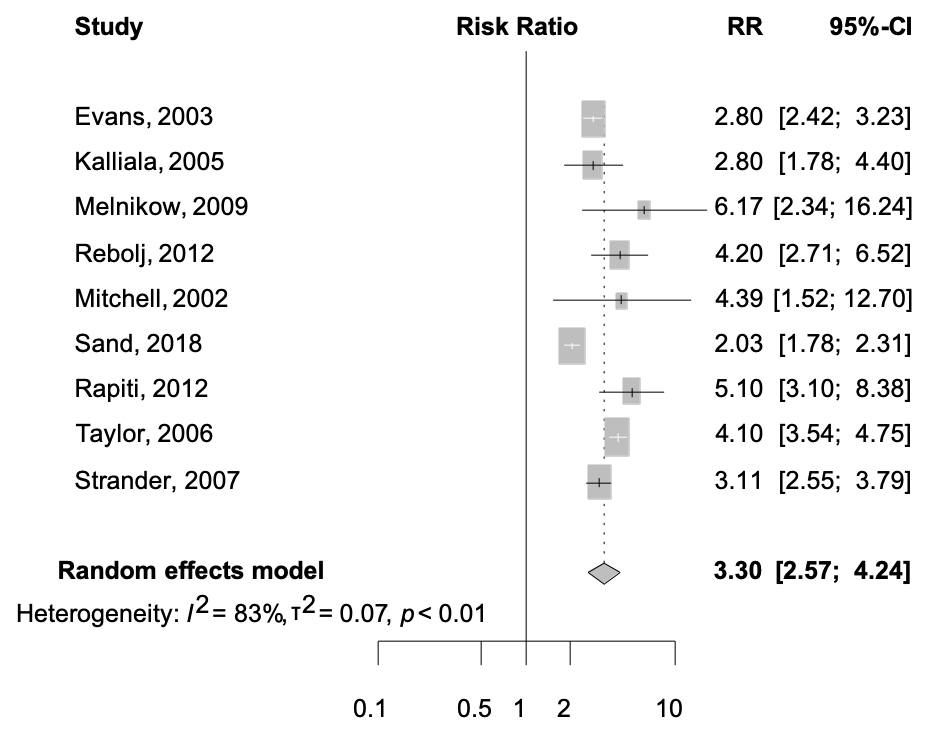


**Age at CIN treatment: <50y (adjusted Hartung-Knapp-Sidik-Jonkman)**


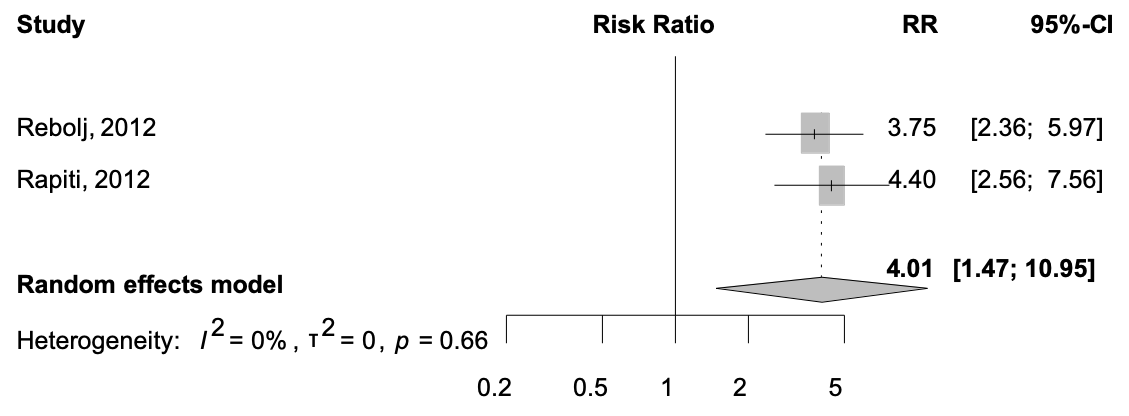


**Age at CIN treatment: <50y (inverse variance)**


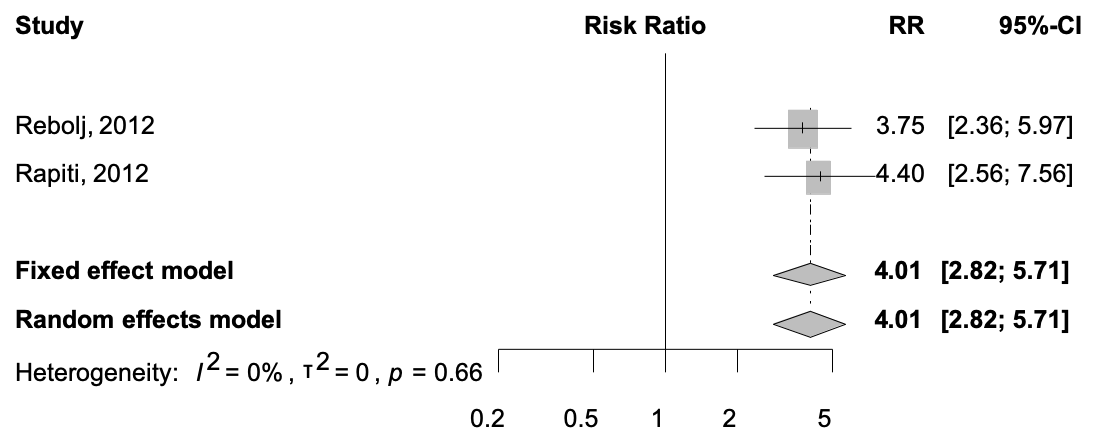


**Age at CIN treatment: ≥50y (adjusted Hartung-Knapp-Sidik-Jonkman)**


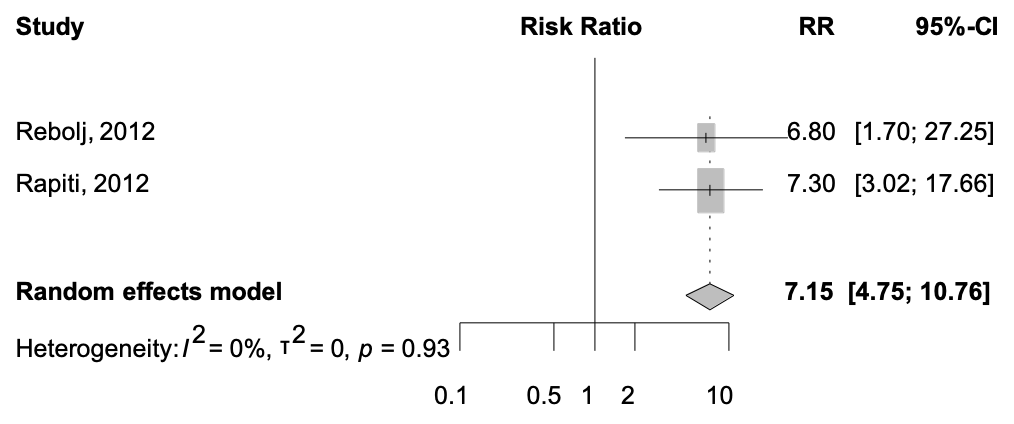


**Age at CIN treatment: ≥50y (inverse variance)**


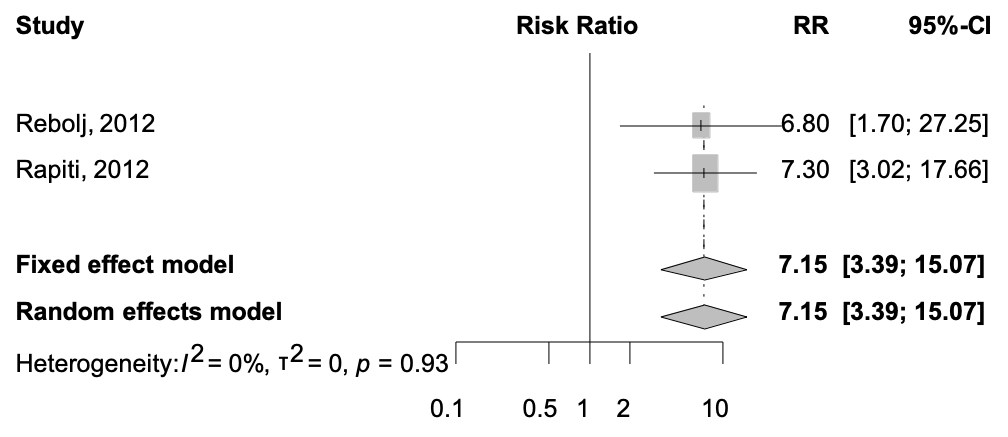


**Treatment method for CIN: excision (adjusted Hartung-Knapp-Sidik-Jonkman)**


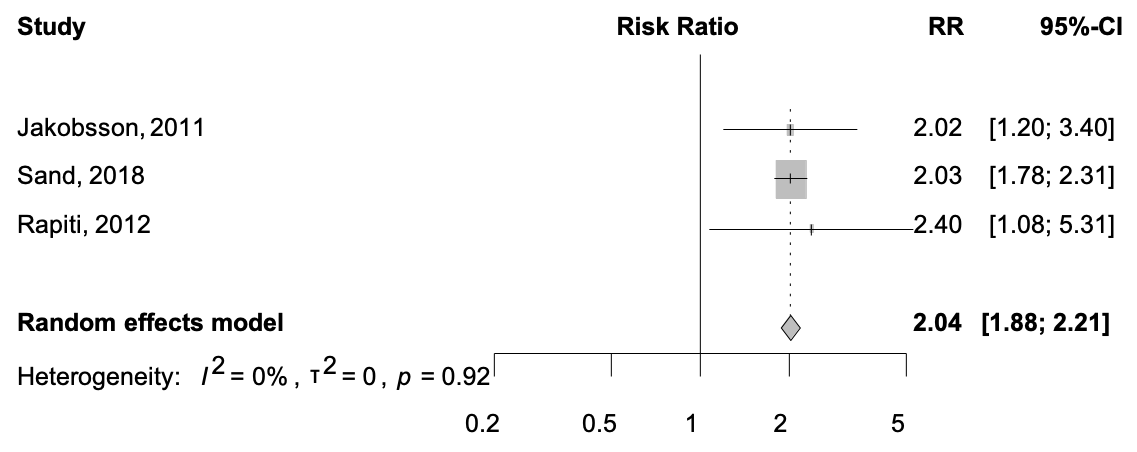


**Treatment method for CIN: excision (inverse variance)**


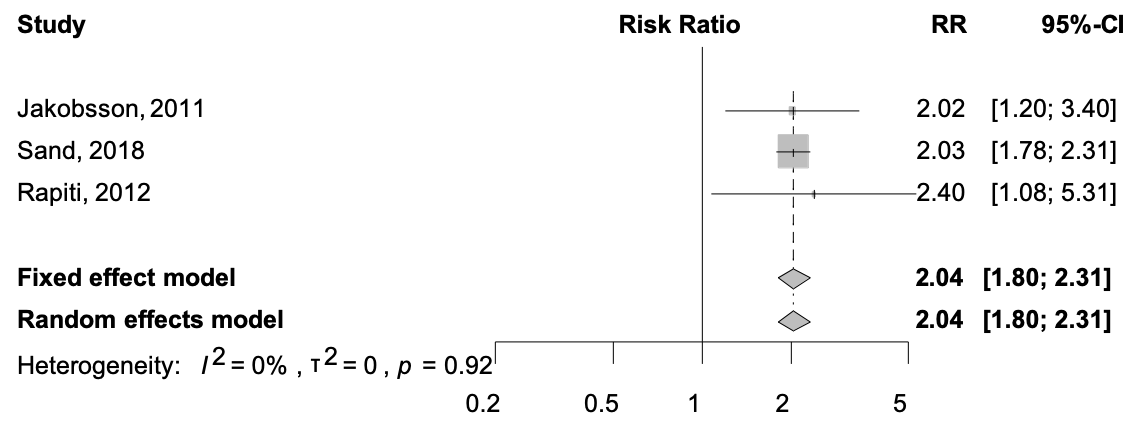


**Treatment method for CIN: ablation (adjusted Hartung-Knapp-Sidik-Jonkman)**


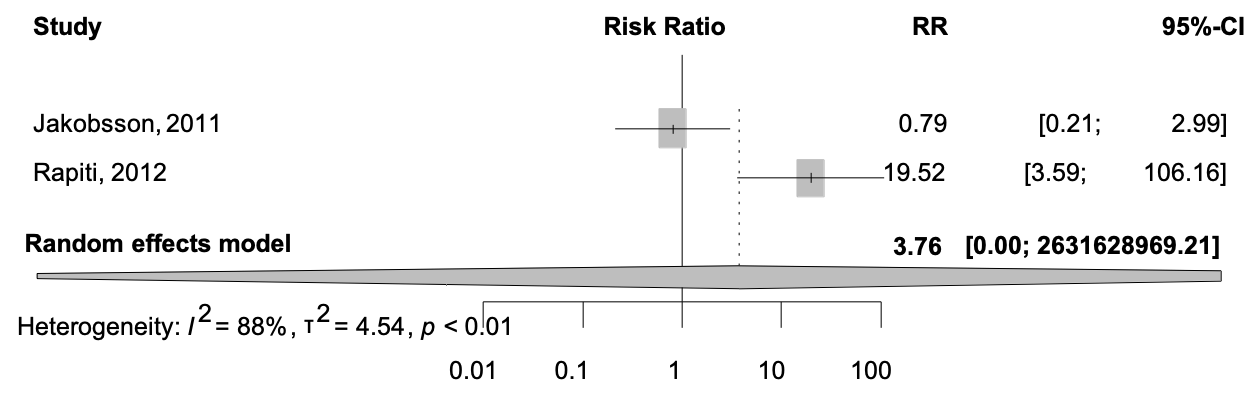


**Treatment method for CIN: ablation (inverse variance)**


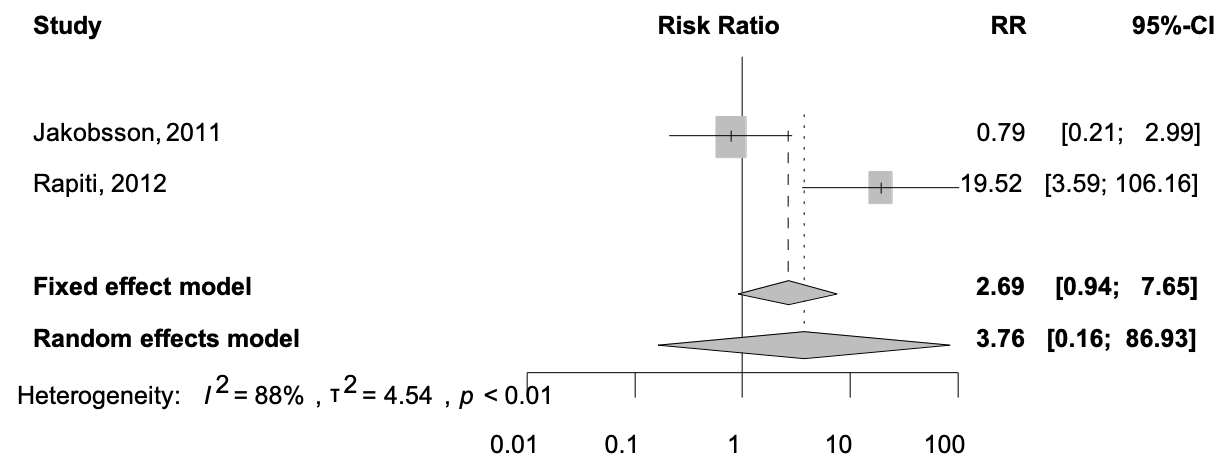


**CIN grade: CIN3**


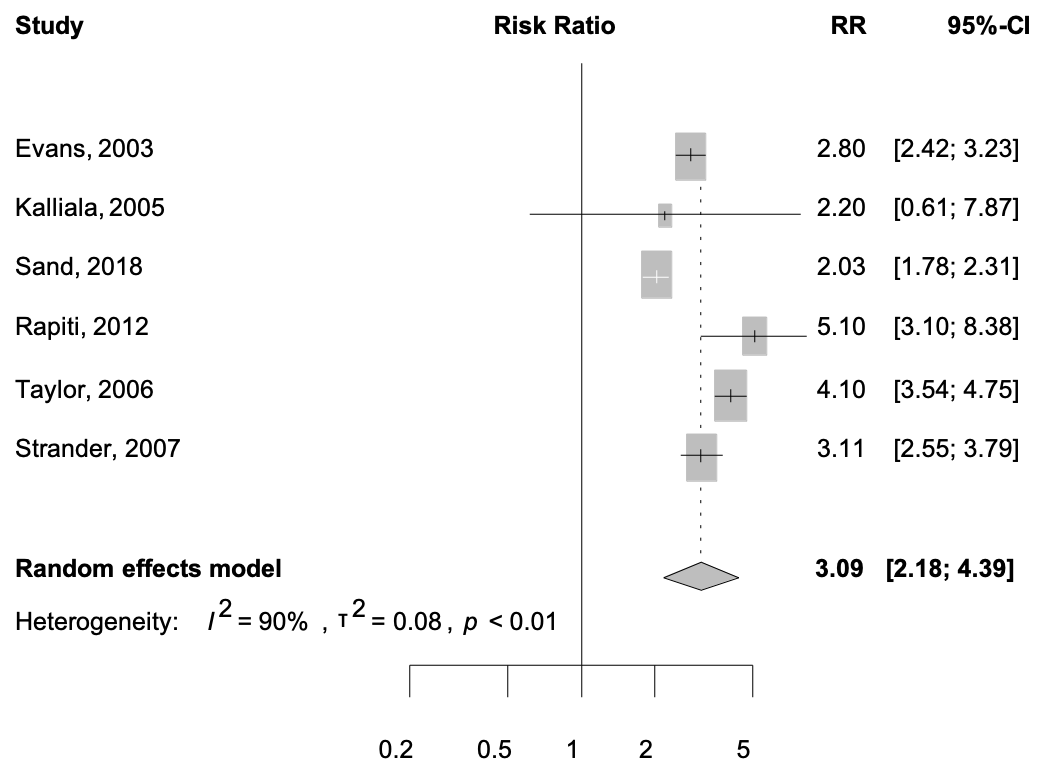


**CIN grade: CIN2/3 (adjusted Hartung-Knapp-Sidik-Jonkman)**


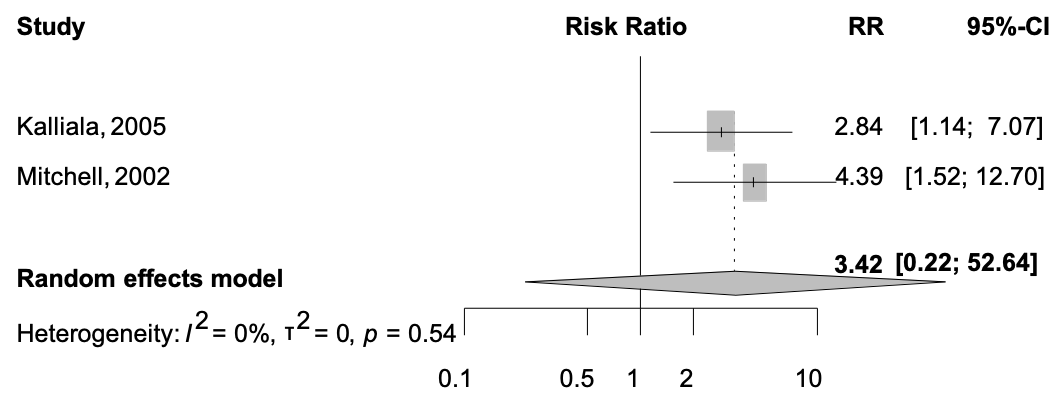


**CIN grade: CIN2/3 (inverse variance)**


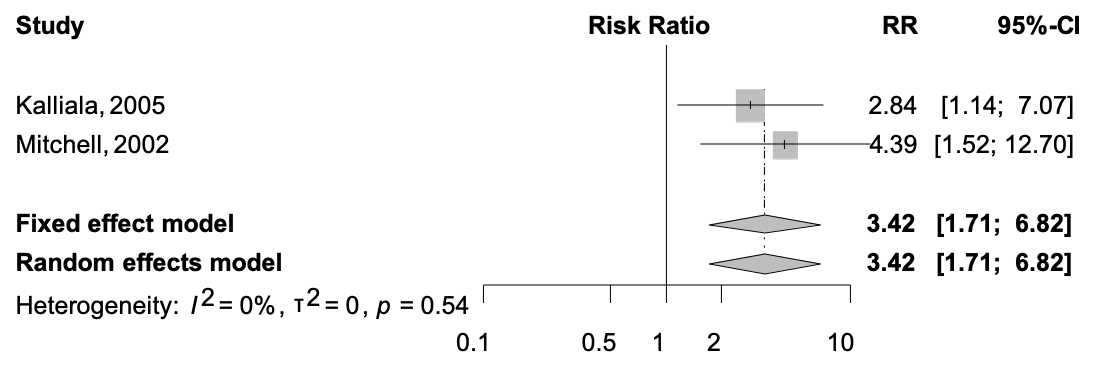


**Length of follow-up after CIN treatment: 0-10y**


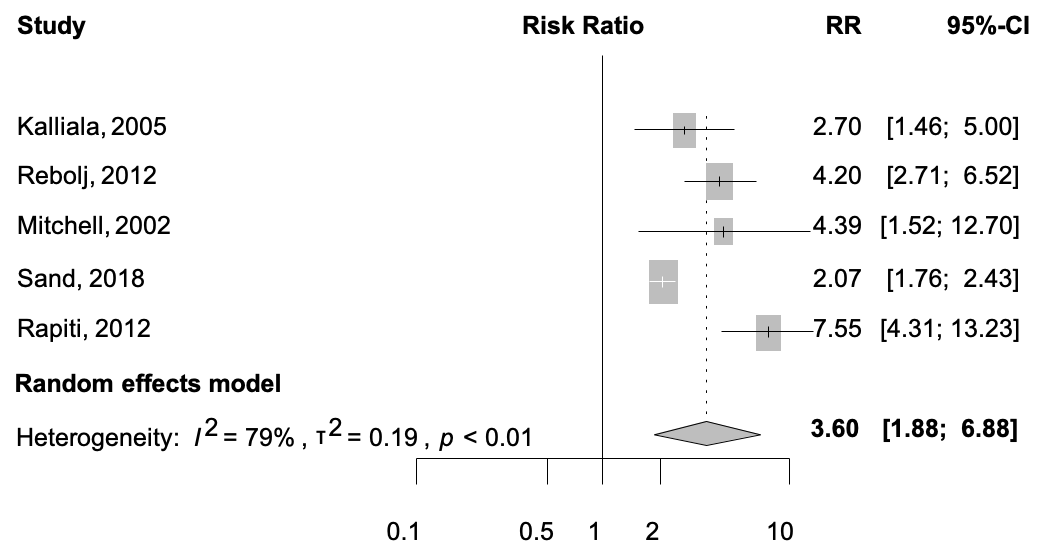


**Length of follow-up after CIN treatment: 10-20y (adjusted Hartung-Knapp-Sidik-Jonkman)**


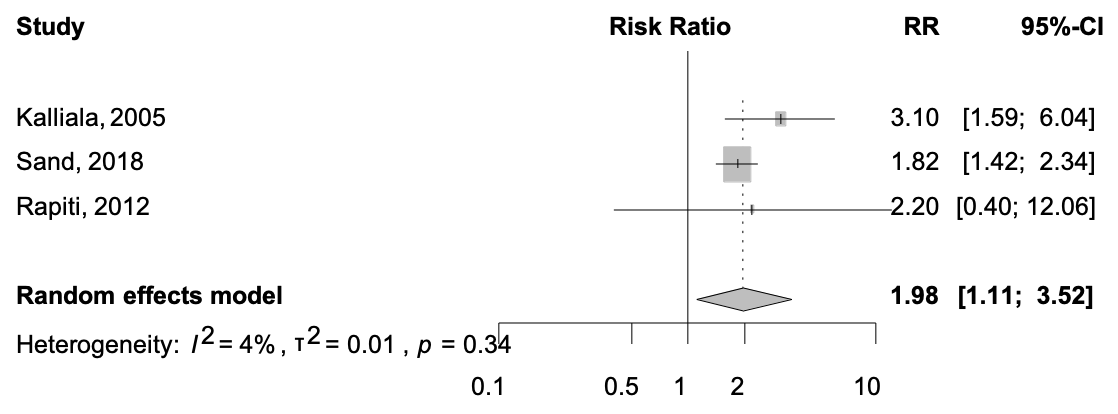


**Length of follow-up after CIN treatment: 10-20y (inverse variance)**


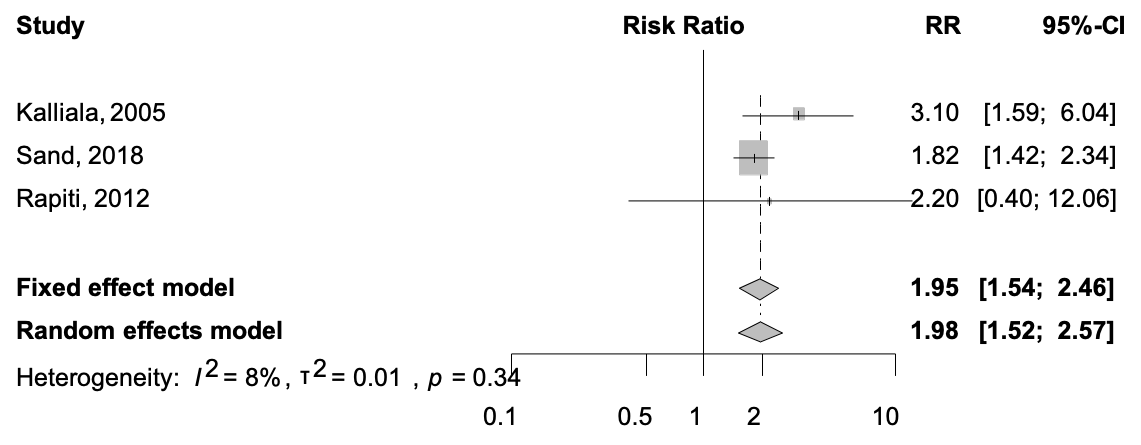


**Length of follow-up after CIN treatment: >20y (adjusted Hartung-Knapp-Sidik-Jonkman)**


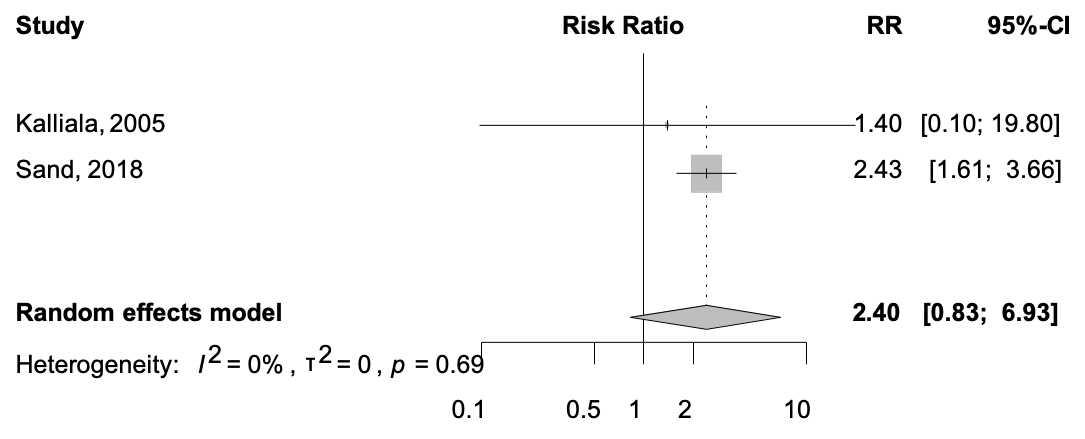


**Length of follow-up after CIN treatment: >20y (inverse variance)**


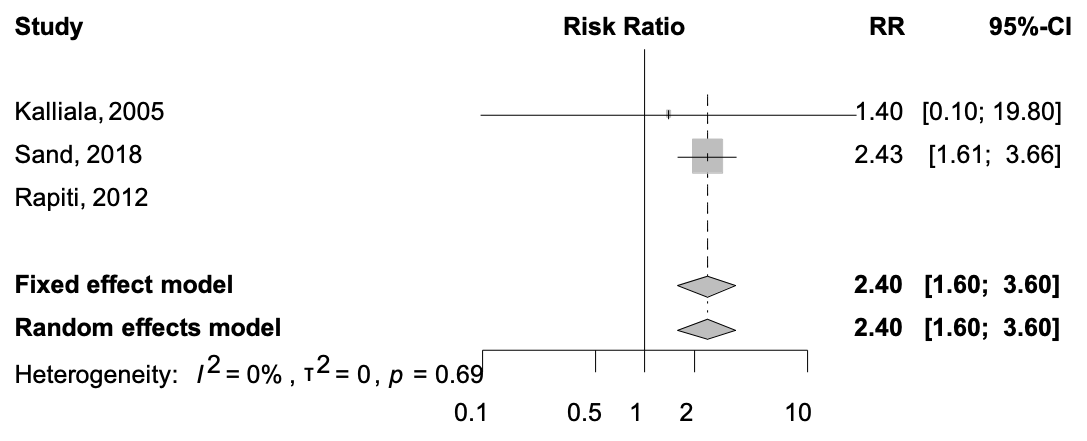


**Length of follow-up after CIN treatment: 0-5y (adjusted Hartung-Knapp-Sidik-Jonkman)**


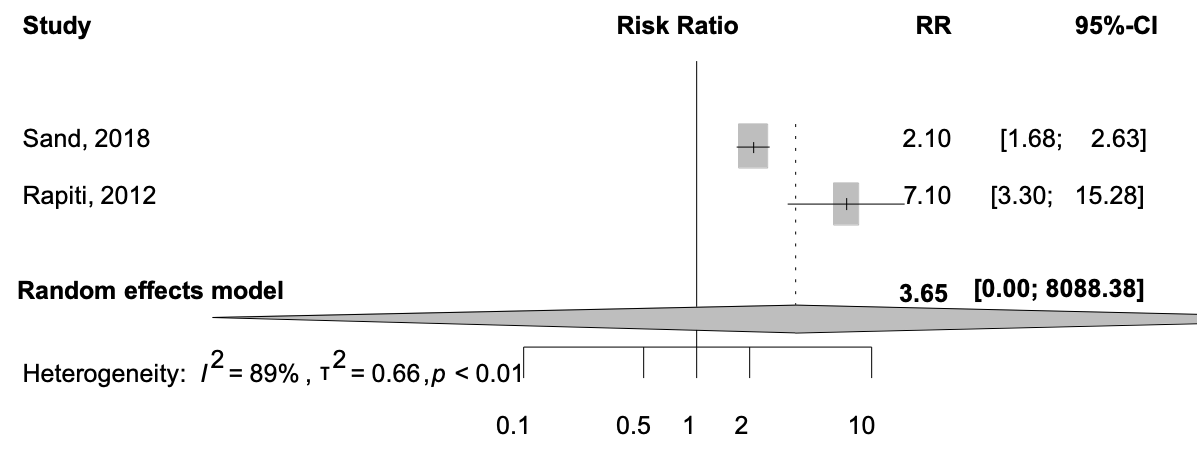


**Length of follow-up after CIN treatment: 0-5y (inverse variance)**


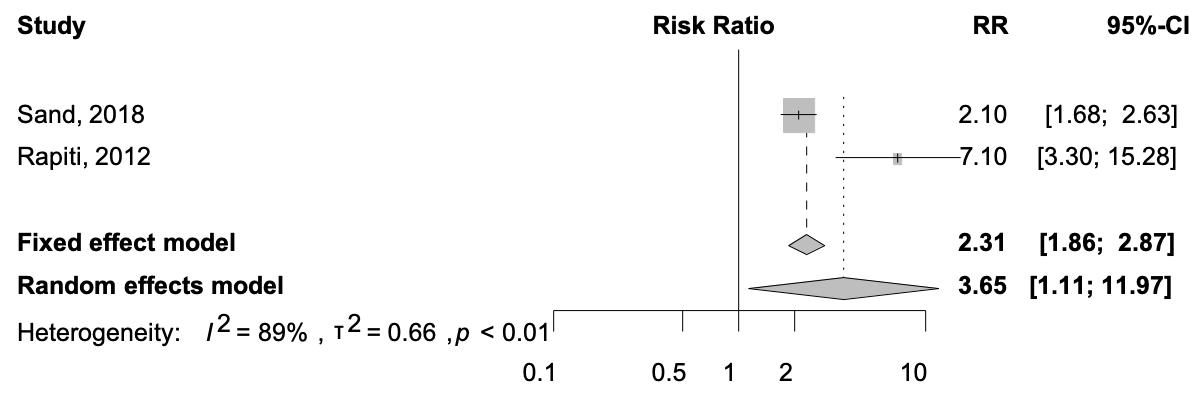


**Length of follow-up after CIN treatment: 5-10y (adjusted Hartung-Knapp-Sidik-Jonkman)**


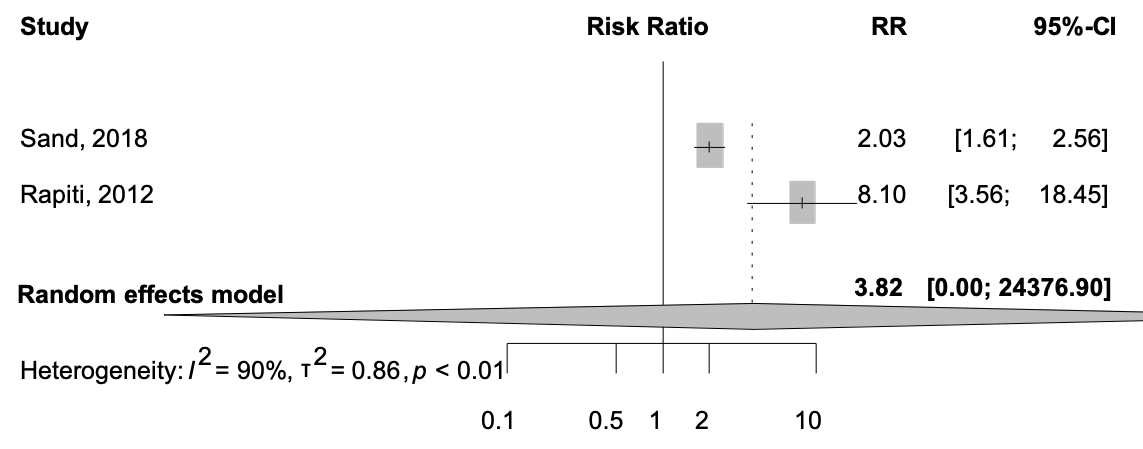


**Length of follow-up after CIN treatment: 5-10y (inverse variance)**


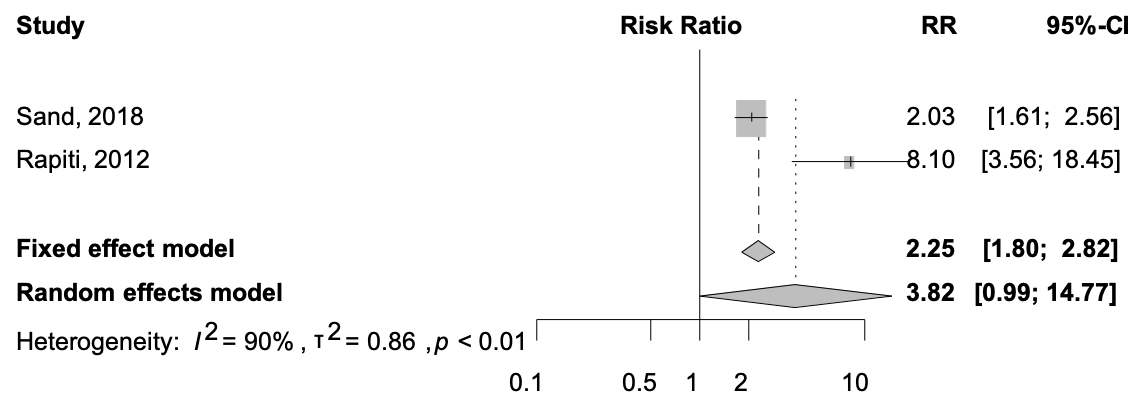


**Length of follow-up after CIN treatment: 10-15y (adjusted Hartung-Knapp-Sidik-Jonkman)**


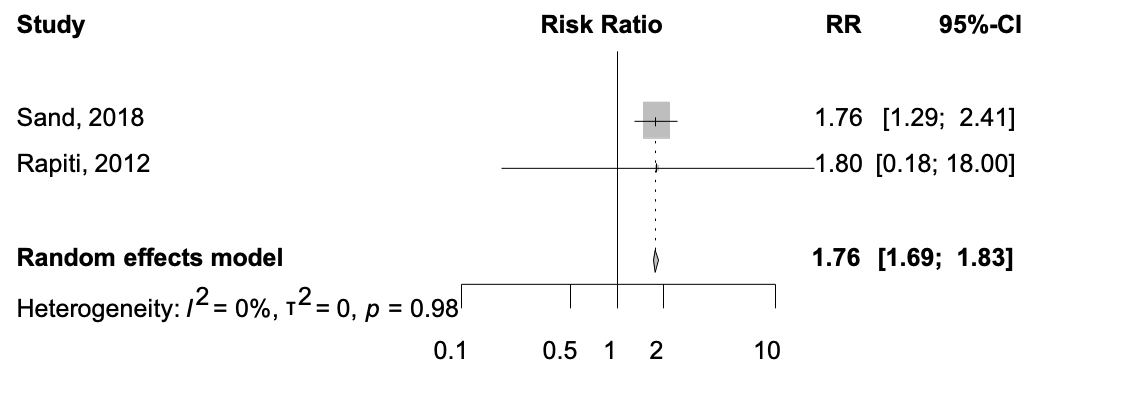


**Length of follow-up after CIN treatment: 10-15y (inverse variance)**


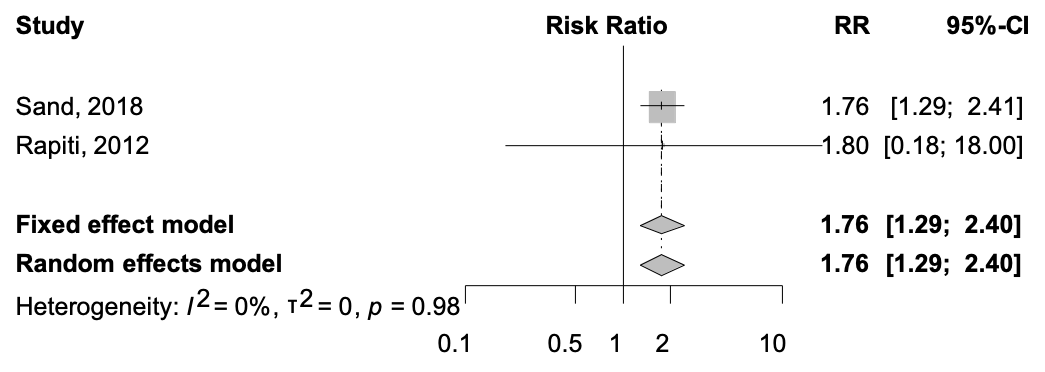


**Length of follow-up after CIN treatment: 15-20y (adjusted Hartung-Knapp-Sidik-Jonkman)**


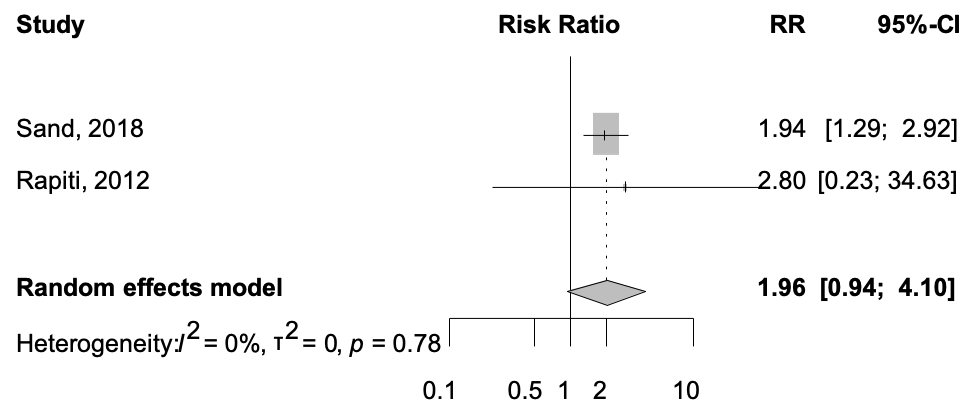


**Length of follow-up after CIN treatment: 15-20y (inverse variance)**


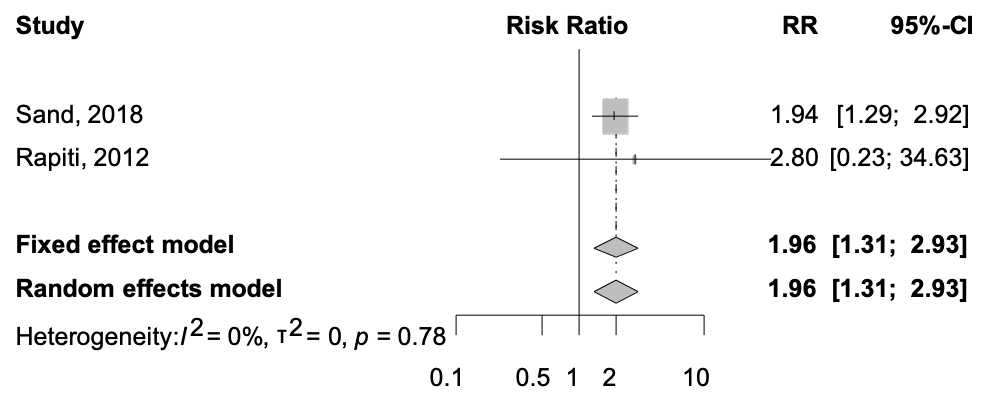


**Length of follow-up after CIN treatment: 0-20y (adjusted Hartung-Knapp-Sidik-Jonkman)**


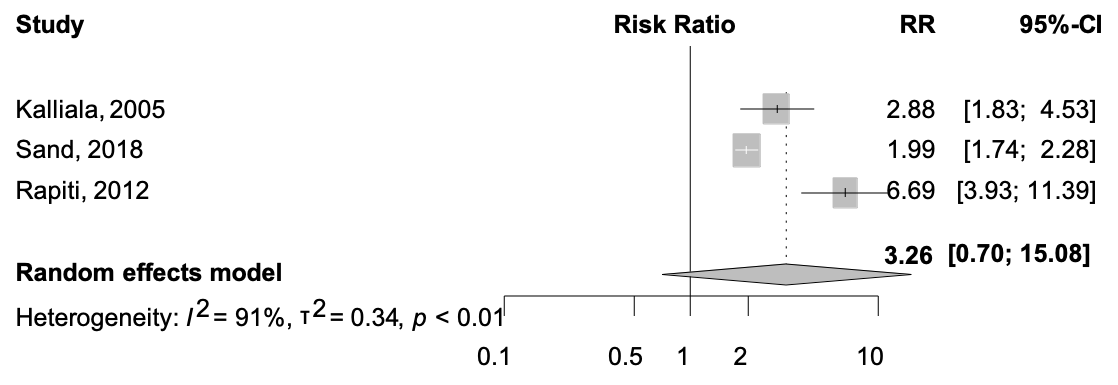


**Length of follow-up after CIN treatment: 0-20y (inverse variance)**


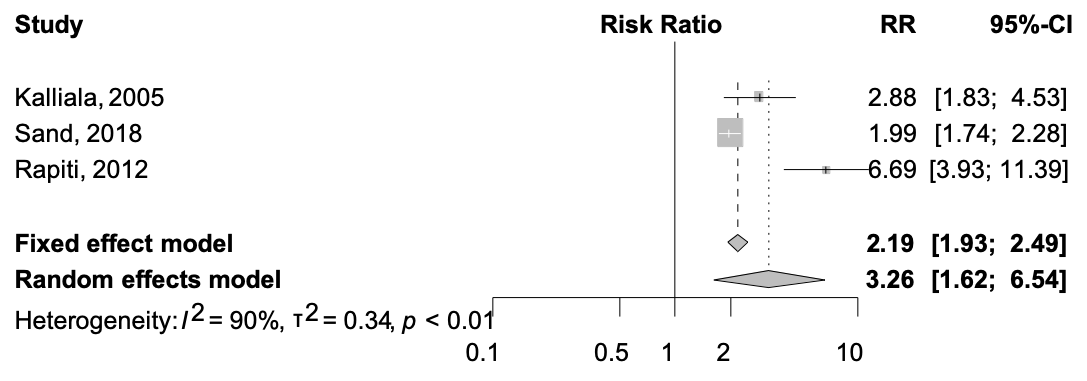

Supplement: Supplementary Figure S2 [file mmc3.docx]
