# Supplementary material for: Incidence and mortality from cervical cancer and other malignancies after treatment of cervical intraepithelial neoplasia: a systematic review and meta-analysis of the literature
Source: Ann Oncol. 2020 Feb;31(2):213–27. doi: 10.1016/j.annonc.2019.11.004 (PMC7479506; doi:10.1016/j.annonc.2019.11.004)

**Supplementary Figures 3:** Pooled relative incidence of cervical and other cancers and mortality from cervical and vaginal cancer after treatment of CIN as compared to the reference population.

**Incidence of cervical cancer**


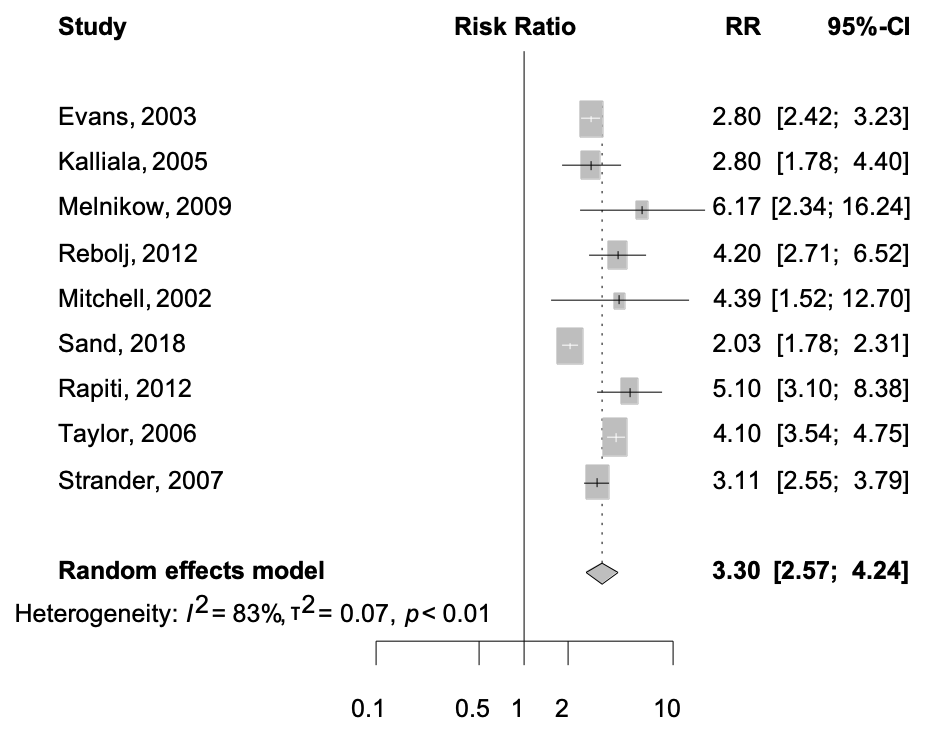


**Incidence of cervical & vaginal cancer (adjusted Hartung-Knapp-Sidik-Jonkman)**


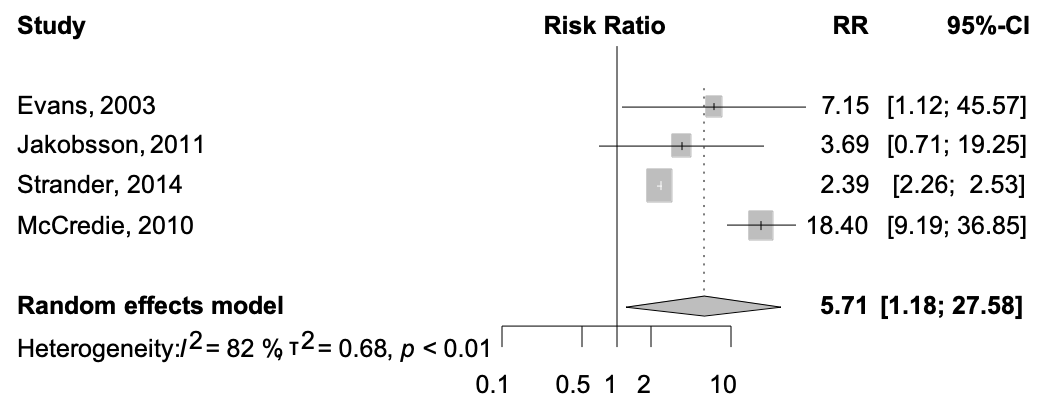


**Incidence of cervical & vaginal cancer (inverse variance)**


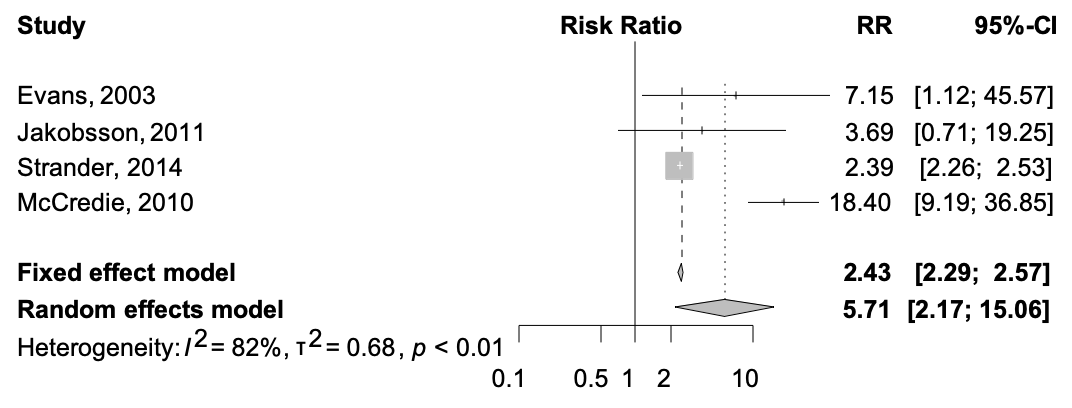


**Incidence of vaginal cancer**


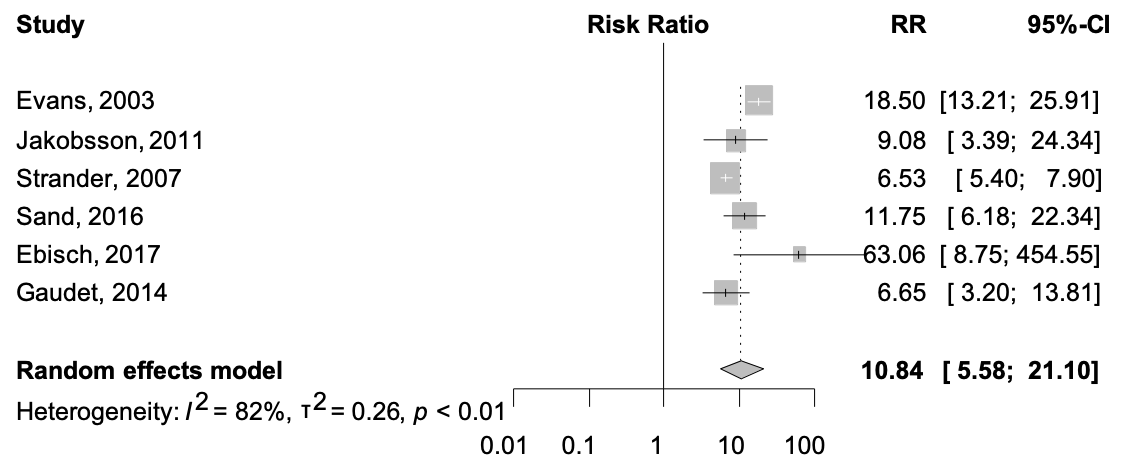


**Incidence of vulvar cancer**


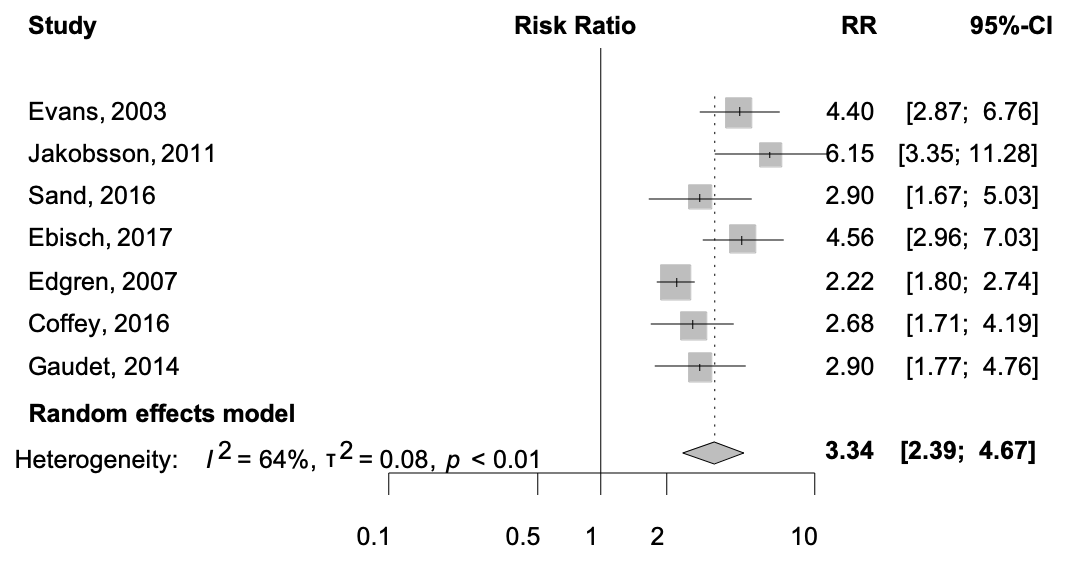


**Incidence of anal cancer**


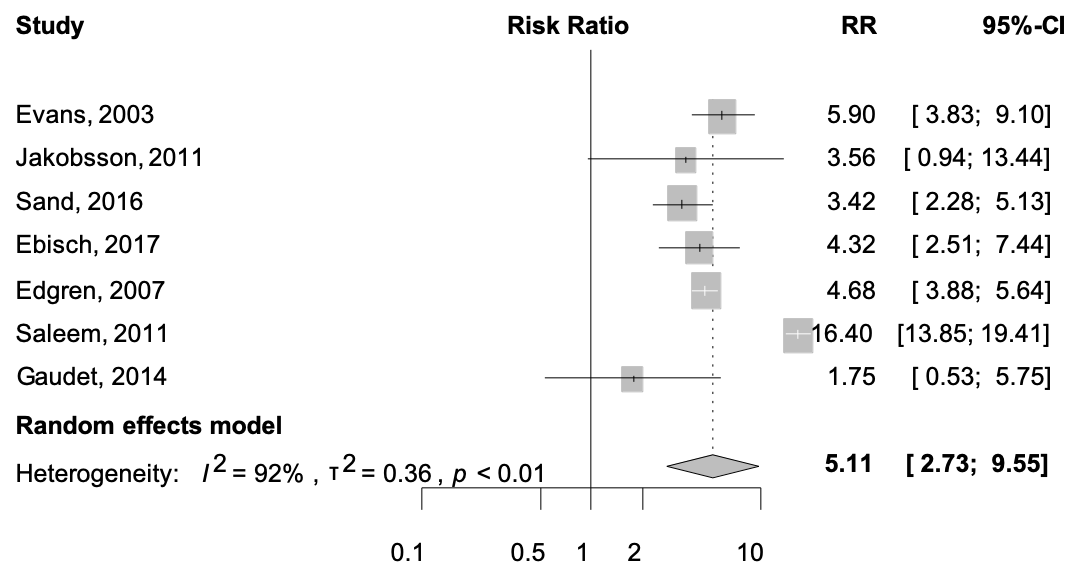


**Incidence of female HPV-related anogenital cancers (anal-vulvar-vaginal-cervical)**


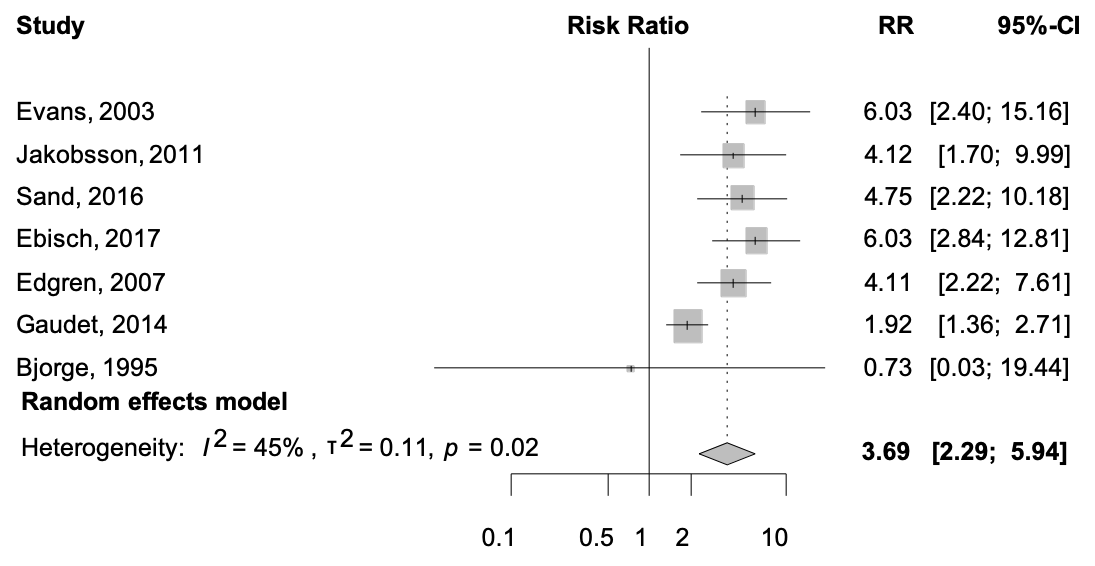


**Incidence of endometrial cancer**


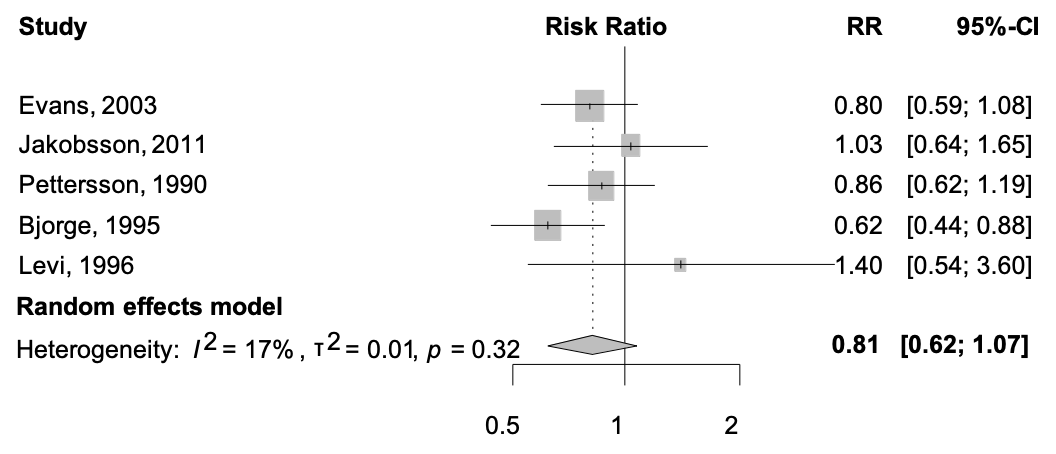


**Incidence of ovarian cancer**


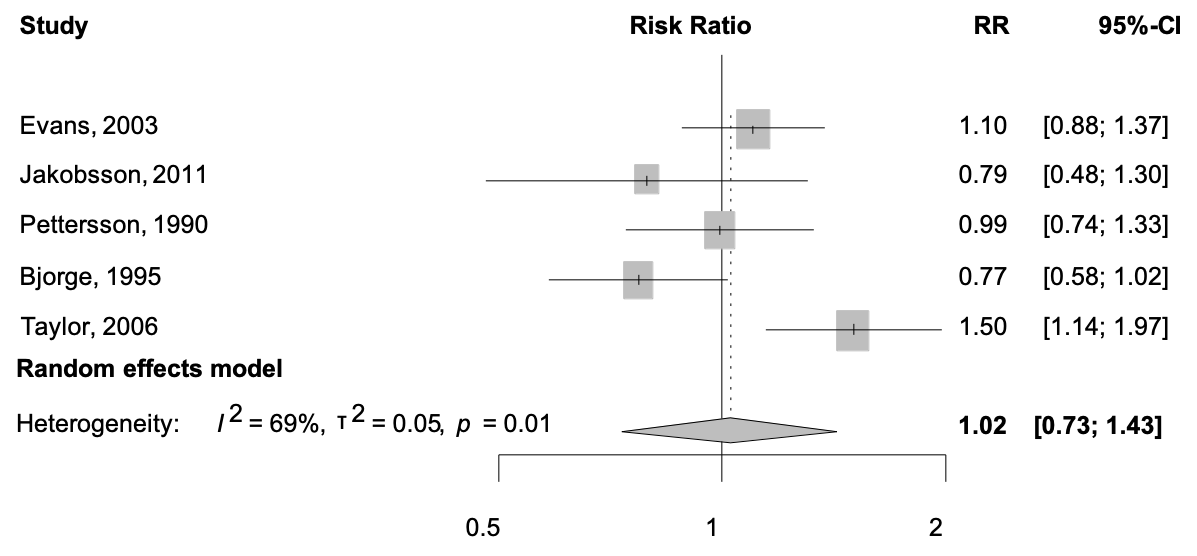


**Incidence of breast cancer**


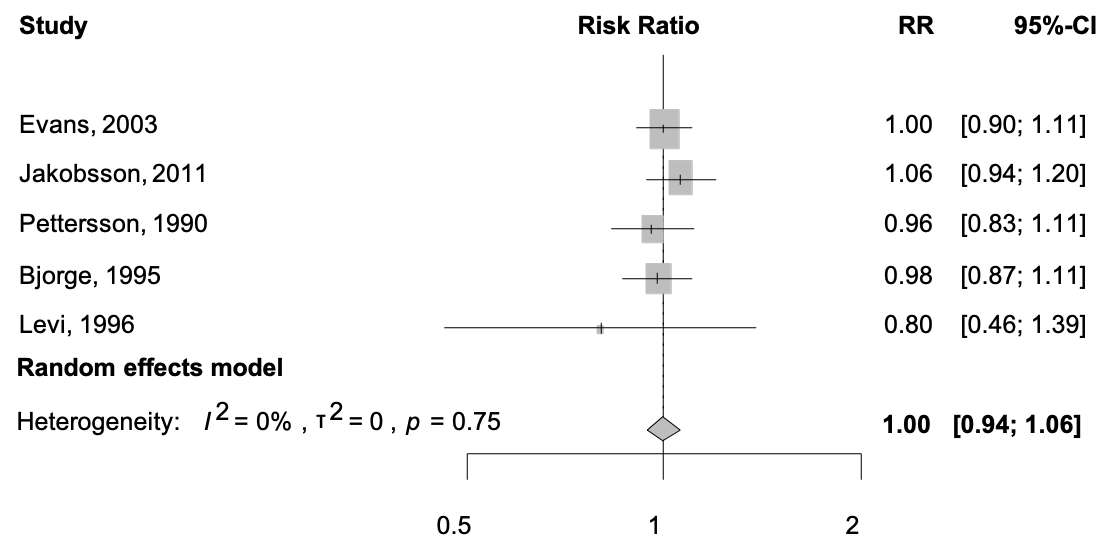


**Incidence of lung cancer**


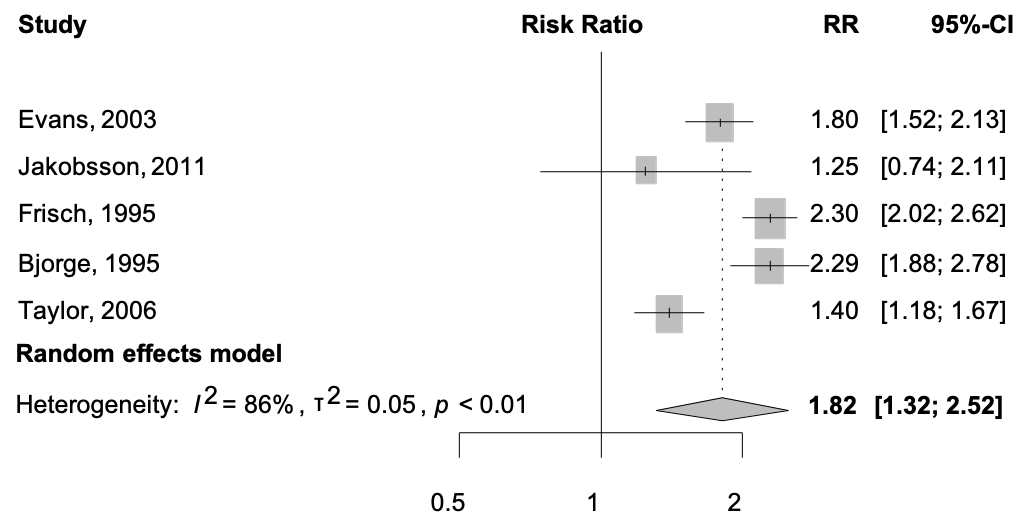


**Incidence of colorectal cancer**


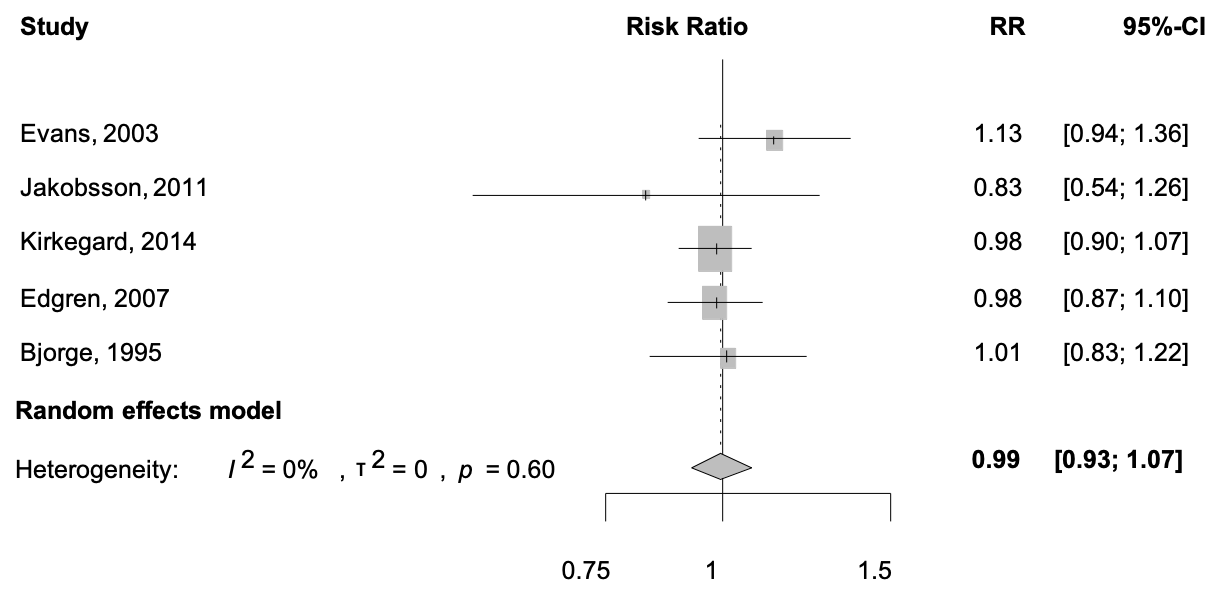


**Incidence of any cancer (adjusted Hartung-Knapp-Sidik-Jonkman)**


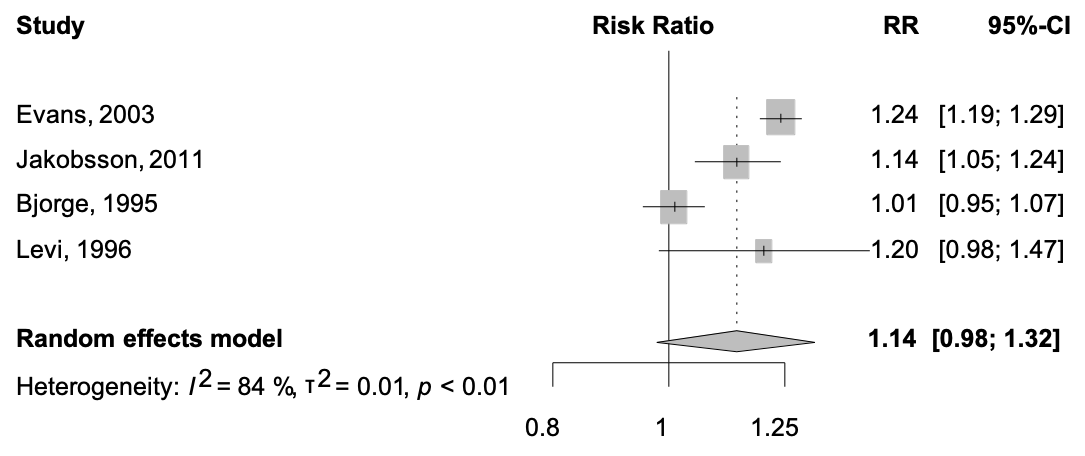


**Incidence of any cancer (inverse variance)**


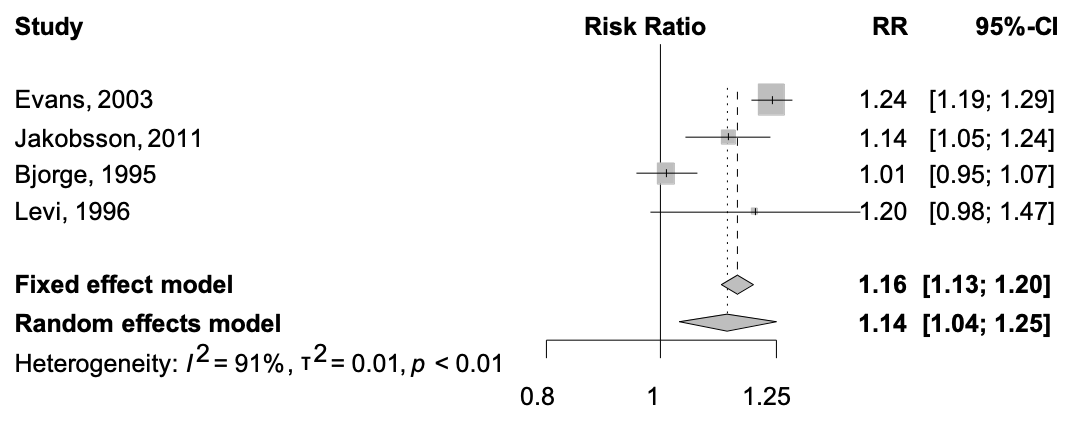


**Mortality from cervical/vaginal cancer (adjusted Hartung-Knapp-Sidik-Jonkman)**


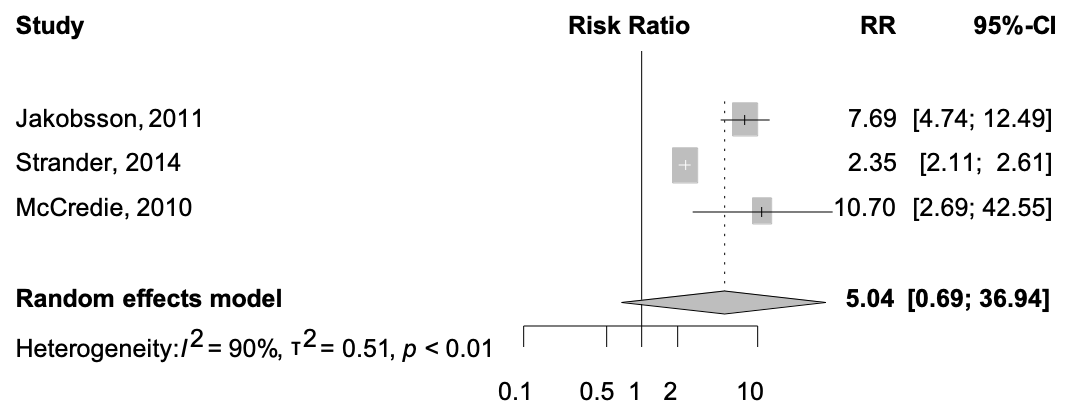


**Mortality from cervical/vaginal cancer (inverse variance)**


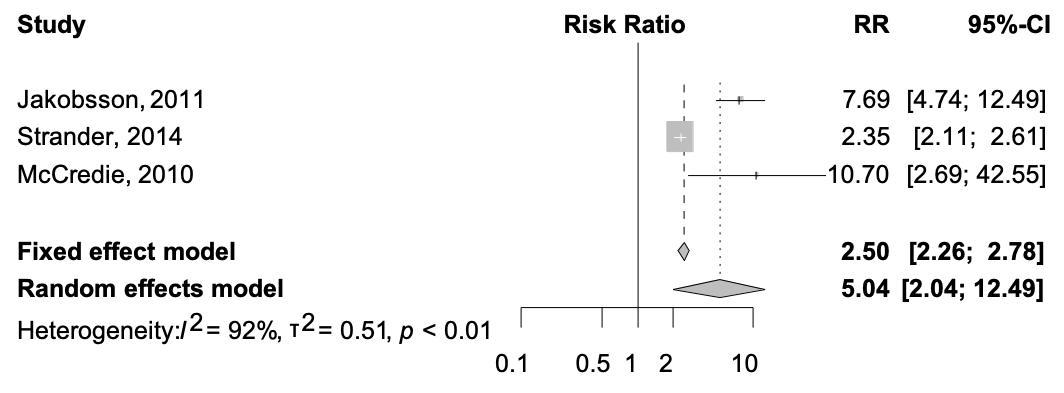

Supplement: Supplementary Figure S3 [file mmc4.docx]
