# Supplementary material for: Incidence and mortality from cervical cancer and other malignancies after treatment of cervical intraepithelial neoplasia: a systematic review and meta-analysis of the literature
Source: Ann Oncol. 2020 Feb;31(2):213–27. doi: 10.1016/j.annonc.2019.11.004 (PMC7479506; doi:10.1016/j.annonc.2019.11.004)
Supplement: Supplementary Table S2 [file mmc6.docx]

**Supplementary Table 2:** Duplicate studies and reasons for exclusion

| **Excluded Studies** | **Country** | **Treatment period** | **FU period** | **Outcomes** | **Included Studies** | **Treatment period** | **FU period** | **Reason for exclusion** |
| --- | --- | --- | --- | --- | --- | --- | --- | --- |
| **Pettersson, 1989** | Sweden | 1958-1981 | NR | Cervical Ca | **Strander, 2007** | 1958-2002 | Up to 2005 | Both studies used the Swedish Cancer Registry for the ascertainment of the outcome and the exposure. Pettersson 1989 is an older study. |
| **Hemminki, 2000** | Sweden | 1958-1996 | 1985-1996 | Cervical Ca | **Strander, 2007** | 1958-2002 | Up to 2005 | Both studies used the Swedish Cancer registry for the ascertainment of the outcome and the exposure. Hemminki 2000 is an older study with no lag period. |
| **Hakama, 2004** | Finland | 1953-2000 | Up to 2001 | Mortality | **Jakobsson, 2009** | 1986-2003 | Up to 2006 | Both studies are population-based in Finland. We included Jakobsson 2009, as this reports on cervical cancer-related mortality for meta-analytical pooling, whilst Hakama 2004 only reports on overall mortality. |
| **McCredie, 2008** | New Zealand | 1955-1976 | Up to 2000 | Other Ca (cervical/vaginal Ca) | **McCredie, 2010** | 1965-1974^1^ | Up to 2000 | The population and setting are similar in both studies. We included McCredie 2010 in the analysis as the study provided data to allow exclusion of hysterectomies for the period 1965-1974, which was not possible in McCredie 2008. Furthermore, it reports on incidence of cervical and vaginal cancer separately, which is not the case in McCredie 2008. |
| **Kalliala, 2010** | Finland | 1974-2001 | Up to 2005 | Mortality | **Jakobsson, 2009** | 1986-2003 | Up to 2006 | Both are population-based studies from Finland. We included Jakobsson 2009 because of the larger cohort size (treated women in Kalliala 2010: 7,104; treated women in Jakobsson 2009: 25,827) |
| **Jakobsson, 2011** | Finland | 1986-2004 | Up to 2006 | Cervical Ca | **Kalliala, 2005/2007** | 1974-2001 | Up to 2003 | Both studies used the Finnish Cancer Registry for the ascertainment of the outcome. We used Kalliala for the main analysis in preference to Jakobsson. The lag period in Jakobsson varied from 0 to 12 months, while in Kalliala this was 6 months for all women. For the subgroup analysis according to type of treatment (excision or ablation), we used the data from Jakobsson as Kalliala did not provide this kind of information. |
| **Silfverdal, 2011** | Sweden | NR | 1999-2001 | Cervical Ca | **Strander, 2007** | 1958-2002 | Up to 2005 | Silfverdal 2011 is a population-based case-control study, while Strander 2007 is a population-based retrospective cohort. |

^1^ The whole study period is 1955-1976. We only included data from 1965-1974 in our analysis as the authors provide data for different treatments separately allowing the exclusion of hysterectomies

Abbreviations:

Ca: cancer; fu: follow-up; NR: not reported
