# Supplementary material for: Incidence and mortality from cervical cancer and other malignancies after treatment of cervical intraepithelial neoplasia: a systematic review and meta-analysis of the literature
Source: Ann Oncol. 2020 Feb;31(2):213–27. doi: 10.1016/j.annonc.2019.11.004 (PMC7479506; doi:10.1016/j.annonc.2019.11.004)
Supplement: Supplementary Table S3 [file mmc7.docx]

**Supplementary Table 3:** Risk of bias in the included studies assessed using the QUIPS-tool

| **Author, Year** | **Study Participation** | **Study Attrition** | **Prognostic Factor Measurement** | **Outcome Measurement** | **Study Confounding** | **Statistical Analysis and Reporting** |
| --- | --- | --- | --- | --- | --- | --- |
| **Pettersson, 1990** | Low bias (women with a diagnosis of CIN3 were drawn from a population-based registry) | Low bias | Moderate bias (conisation was the usual procedure and hysterectomies only in a few cases, without any further details) | Low bias | Low bias | Low bias |
| **Bjorge, 1995** | Low bias (women with a histological diagnosis of CIN3 were drawn from a population-based registry) | Low bias | Moderate bias (conisation was the usual procedure and no further details were provided; treatment period was 1970-1992, when hysterectomy for CIN3 was still common in Norway – this study was therefore excluded from cervical cancer incidence analysis) | Low bias | Low bias | Low bias |
| **Frisch, 1995** | Low bias (women with a diagnosis of CIN3 were drawn from a population-based registry) | Low bias | Moderate bias (not clearly reported if all women received treatment but likely they had as CIN3 is not managed expectantly; inclusion of women with hysterectomy is possible) | Moderate bias (registry but no lag period) | Low bias | Low bias |
| **Levi, 1996** | Low bias (women with a histological diagnosis of CIN3 were drawn from a population-based registry) | Low bias | Moderate bias (no information about treatment; not clearly reported if all women received treatment but likely they had as CIN3 is not managed expectantly; inclusion of women with hysterectomy is possible; treatment period was 1974-1993, when hysterectomy for CIN3 was still common - this study was therefore excluded from cervical cancer incidence analysis) | Unclear (registry but unclear if there is a lag period) | Low bias | Low bias |
| **Mitchell, 2002** | Low bias (women were drawn from the records of a statewide registry where participation in screening is high (81%); histological diagnosis of CIN2-3) | Low bias (no rates/description for those lost to follow-up - stated that many women had a lifetime screening record) | Moderate bias (Victorian Cervical Cytology Registry holds incomplete information on treatment, although most must have been treated as only CIN2+ cases were included; hysterectomies were excluded) | Low bias | Moderate bias (no adjustment for age) | Low bias |
| **Evans, 2003** | Low bias (women with a diagnosis of CIN3 were drawn from a population-based registry) | Low bias (no rates/description for those lost to follow-up - through a population-based registry and therefore losses to follow-up expected to be low) | Low bias (not clearly reported if all women received treatment but likely they had as CIN3 is not managed expectantly; types of treatment not described; <10% had radical surgery) | Moderate bias (registry but no lag period) | Low bias | Low bias |
| **Taylor, 2006** | Low bias (women with a histological diagnosis of CIN3 were drawn from a population-based registry) | Low bias | Moderate bias (not clearly reported if all women received treatment but likely they had as CIN3 is not managed expectantly; because Tx period was 1988-1999, hysterectomy rates likely to be low) | Moderate bias (registry but no lag period) | Low bias | Low bias |
| **Edgren, 2007** | Low bias (women with a histological diagnosis of CIN3 were drawn from a population-based registry) | Low bias | Low bias (the common CIN treatments in Sweden are reported with no further details; 5% received hysterectomy) | Low bias | Low bias | Low bias |
| **Kalliala, 2005 & 2007** | Low bias (women from a large University Hospital; histological diagnosis of CIN1-3) | Low bias (follow-up through a population-based registry and losses to follow-up very low (<1-2%) due to emigration, death or incorrect information) | Low bias | Low bias | Low bias | Low bias |
| **Strander, 2007 & 2014** | Low bias (women from a population-based registry; histological diagnosis of CIN3) | Low bias | Moderate bias (Swedish Cancer Registry does not included data on treatment; hysterectomies were included; as hysterectomy was the preferred treatment for CIN3 in 60-70’s women treated during 1958-1980 were excluded from cervical cancer incidence; women treated during 1981-2000 were included) | Low bias | Low bias | Low bias |
| **Jakobsson, 2009 & 2011** | Moderate bias (women from a population-based registry; no information on the histopathological diagnoses, - some women may have received treatment for non-CIN lesions) | Low bias (no rates/description for losses to follow-up, as follow-up through a population-based registry and rates are expected to be low) | Low bias (9.1% received radical treatment such as cervical amputation) | Low bias (lag period varied from 0 to 12 months) | Low bias | Low bias |
| **Melnikow, 2009** | Low bias (women from a population-based registry; histological diagnosis of CIN1-3) | Low bias (no rates/description for losses to follow-up, as follow-up through a population-based registry and rates are expected to be low) | Low bias | Low bias | Moderate bias (no adjustment for age) | Low bias |
| **McCredie, 2010** | Low bias (women from a single hospital; ~8% were not included because of missing data or initial diagnosis made elsewhere; histological diagnosis of CIN3) | Moderate bias (follow-up through registries but also through hospital records and histopathological review) | Low bias | Low bias | Low bias | High bias (selective reporting: results stratified by treatment modality are available only for 1965-74, and not for 1955-64 or 1975-76; outcomes for ring biopsy (“shallow” CKC <1.5-2cm) are reported separately but results for “deeper” CKC are reported with hysterectomy/amputation) |
| **Kocken, 2011** | Low bias (pooled analysis from 3 hospitals; histological diagnosis of CIN2-3) | Low bias (follow-up was population-based and hospital-based; not reported how many did not attend hospital-based follow-up; only 2% were lost to population-based follow-up due to emigration or unrelated death) | Low bias | Low bias | Unclear | Low bias |
| **Saleem, 2011** | Low bias (women with a diagnosis of CIN3 were drawn from a population-based registry) | Low bias | Moderate bias (not clearly reported if all women received treatment but likely they had as CIN3 is not managed expectantly; types of treatments are not described; hysterectomies may have been included) | Low bias | Low bias | Low bias |
| **Kreimer, 2012** | Moderate bias (population-based study in a rural province that received treatment for CIN2+: 96.5% of eligible treated women participated in the second (current) study; women were treated because of HSIL in cytology or CIN2+ on biopsy or colposcopy; women treated for non-biopsy-confirmed CIN2+ were included) | Low bias (a cancer registry was used for finding women with cervical cancer after CIN treatment even if they didn’t attend post-treatment visit) | Low bias | Moderate bias (registry but no lag period) | Unclear | Low bias |
| **Rapiti, 2012** | Low bias (women were drawn from a population-based registry; only 10% the diagnosis of CIN3 was cytological) | Low bias | Moderate bias (4% had no treatment for CIN3; number of treated patients with hysterectomy is not reported, but hysterectomy was upon patient’s request) | Low bias | Low bias | Low bias |
| **Rebolj, 2012** | Low bias (population-based registry but women with 0.5% of the most common four-letter combinations in maiden names were excluded to avoid false identity matches (in the pathology register, women were identified by their sex, birth date, and the first four letters of their maiden name) - exclusion 30% of women but no expected bias; histological diagnosis of CIN1-3) | Low bias (no rates/description for those lost to follow-up - through a population-based registry and therefore losses to follow-up expected to be low) | Moderate bias (type of treatment not registered consistently - only patients treated during 90’s or 00’s were included, we assumed that hysterectomy was performed in relatively few cases) | Moderate bias (registry but no lag period) | Low bias | Low bias |
| **Gaudet, 2014** | Low bias (women from a population-based registry with a histological diagnosis of CIN2-3) | Low bias | Moderate bias (not clearly reported if all women received treatment but likely they had as CIN2+ is not managed expectantly; types of treatments are not described; hysterectomies may have been included) | Low bias | Low bias | Low bias |
| **Kirkegard, 2014** | Moderate bias (all eligible women from a population-based registry; no description of CIN grade) | Low bias | Low bias (treatment was cervical conisation described as “a minor surgical procedure”) | Moderate bias (lag period for the time window 1-5y but not for the overall time window) | Low bias | Low bias |
| **Coffey, 2016** | Moderate bias (nested case-control study; not reported if diagnosis of CIN3 was histological or cytological) | Low bias | Moderate bias (not clearly reported if all women received treatment but likely they had as CIN3 is not managed expectantly; types of treatments are not described; hysterectomies may have been included, but effect estimate was adjusted for prior hysterectomy) | Low bias | Low bias | High bias (nested case-control study; reference population was women with vulvar cancer but no previous CIN3 diagnosis) |
| **Sand, 2016** | Low risk (women from population-based registries with histologically-confirmed CIN2-3; although registration of exposure was not entirely complete until mid-1990s, the study was ranked as low risk because of the large number of participants) | Low bias | Moderate bias (not clearly reported if all women received treatment but likely they had as CIN2+ is not managed expectantly; types of treatments are not described; hysterectomies may have been included) | Low bias | Low bias | Low bias |
| **Ebisch, 2017** | Moderate risk (women from a population-based registry with a previous histological diagnosis of CIN3; the first four letters were used as a personal identifier, but not excluding the most common four-letter combinations might have resulted in false identity matches) | Low bias | Moderate bias (not clearly reported if all women received treatment but likely they had as CIN3 is not managed expectantly; types of treatments are not described; hysterectomies may have been included) | Low bias | Low bias | Low bias |
| **Sand, 2018** | Low risk (population-based registries with histologically-confirmed CIN3; although registration of exposure was not entirely complete until mid-1990s, the study was ranked as low risk because of the large number of participants) | Low bias | Low bias | Low bias | Low bias | Low bias |

Abbreviatioan:

CIN: cervical intraepithelial neoplasia; CKC: cold knife conisation
