# Supplementary material for: Incidence and mortality from cervical cancer and other malignancies after treatment of cervical intraepithelial neoplasia: a systematic review and meta-analysis of the literature
Source: Ann Oncol. 2020 Feb;31(2):213–27. doi: 10.1016/j.annonc.2019.11.004 (PMC7479506; doi:10.1016/j.annonc.2019.11.004)
Supplement: Supplementary Table S4 [file mmc8.docx]

**Supplementary table 4:** Pooled incidence rate of cervical cancer per 100,000 woman-years. Subgroup analyses according to age at CIN treatment; treatment method; CIN grade; length of follow-up. Sensitivity analyses including only studies at low or moderate risk of bias in all domains; with lag period; with histological diagnosis of CIN; without women with untreated CIN; without women treated with hysterectomy before cancer diagnosis; according to continent.

| **Cervical Cancer** | **Studies** | **Outcomes** | **Woman-years** | **IR (95% CI) per 100,000 women-years**  **(95% PI)** | **Q-test (*P*-value)** | **I^2^** | **τ^2^** |
| --- | --- | --- | --- | --- | --- | --- | --- |
| **Overall incidence** | 11 | 1155 | 5562889 | 39 (22 to 69)  (6 to 254) | 852·01 (<0·001) | 98·72 | 0·82 |
| *Studies with lag period between CIN diagnosis and cancer* | 8 |  |  | 44 (28 to 69)  (13 to 147) | 37·12 (<0·001) | 96·34 | 0·33 |
| *Studies with histological diagnosis* | 9 |  |  | 35 (19 to 67)  (5 to 250) | 836·47 (<0·001) | 99·03 | 0·89 |
| *Studies with low or moderate risk of bias* | 9 |  |  | 31 (19 to 51)  (7 to 133) | 818·61 (<0·001) | 98·22 | 0·49 |
| *Studies without untreated women* | 9 |  |  | 40 (20 to 79)  (5 to 330) | 839·30 (<0·001) | 99·15 | 1·04 |
| *Studies without hysterectomies before cancer diagnosis* | 6 |  |  | 55 (26 to 118)  (8 to 370) | 31·68 (<0·001) | 97·51 | 0·79 |
| *Europe* | 6 |  |  | 39 (22 to 69)  (6 to 254) | 852·01 (<0·001) | 98·72 | 0·82 |
| *Northern Europe* | 3 |  |  | 35 (26 to 46)  (21 to 58) | 15·43 (<0·001) | 87·79 | 0·05 |
| *Western Europe* | 3 |  |  | 41 (36 to 46)  (36 to 46) | 0·82 (0·664) | 0·00 | 0·00 |
| **Age at CIN treatment** |  |  |  |  |  |  |  |
| <50y | 1 | 18 | 51744 | 35 (20 to 53)  (20 to 53) | N/A (N/A) | N/A | N/A |
| ≥50y | 1 | 2 | 5212 | 38 (1 to 116)  (1 to 116) | N/A (N/A) | N/A | N/A |
| **Treatment method for CIN** |  |  |  |  |  |  |  |
| Excision | 5 | 265 | 797848 | 60 (20 to 179)  (5 to 733) | 49·49 (<0·001) | 94·96 | 1·32 |
| *Studies with lag period between CIN diagnosis and cancer* | 3 |  |  | 87 (25 to 303)  (8 to 906) | 21·64 (<0·001) | 89·91 | 1·02 |
| *Studies with histological diagnosis* | 3 |  |  | 87 (25 to 303)  (8 to 906) | 21·64 (<0·001) | 89·91 | 1·02 |
| *Studies with low or moderate risk of bias* | 4 |  |  | 34 (14 to 80)  (7 to 170) | 26·96 (<0·001) | 86·28 | 0·48 |
| *Europe* | 3 |  |  | 25 (13 to 49)  (8 to 80) | 22·63 (<0·001) | 80·18 | 0·24 |
| Ablation | 1 | 3 | 66601 | 5 (1 to 11)  (1 to 11) | N/A (N/A) | N/A | N/A |
| Cryotherapy | 1 | 2 | 11181 | 18 (0 to 54)  (0 to 54) | N/A (N/A) | N/A | N/A |
| **CIN grade** |  |  |  |  |  |  |  |
| CIN1 | 2 | 14 | 48865 | 29 (17 to 48)  (17 to 48) | 1·10 (0·295) | 0·00 | 0·00 |
| CIN2 | 2 | 9 | 24951 | 36 (19 to 69)  (19 to 69) | 0·50 (0·480) | 0·00 | 0·00 |
| CIN3 | 8 | 961 | 5011905 | 36 (17 to 76)  (4 to 315) | 791·30 (<0·001) | 99·12 | 1·09 |
| *Studies with lag period between CIN diagnosis and cancer* | 6 |  |  | 49 (24 to 98)  (9 to 274) | 29·16 (<0·001) | 97·70 | 0·65 |
| *Studies with histological diagnosis* | 7 |  |  | 35 (15 to 81)  (3 to 364) | 780·71 (<0·001) | 99·32 | 1·25 |
| *Studies with low or moderate risk of bias* | 6 |  |  | 26 (14 to 50)  (5 to 134) | 760·63 (<0·001) | 98·80 | 0·59 |
| *Studies without untreated women* | 7 |  |  | 35 (15 to 81)  (3 to 364) | 780·71 (<0·001) | 99·32 | 1·25 |
| *Studies without hysterectomies before cancer diagnosis* | 3 |  |  | 65 (15 to 271)  (4 to 1023) | 22·30 (<0·001) | 93·36 | 1·45 |
| *Europe* | 6 |  |  | 40 (36 to 44)  (34 to 47) | 9·24 (0·100) | 30·61 | 9·24 |
| *Northern Europe* | 3 |  |  | 39 (34 to 46)  (31 to 49) | 7·54 (0·023) | 51·87 | 0·01 |
| *Western Europe* | 3 |  |  | 40 (35 to 46)  (35 to 46) | 1·67 (0·435) | 0·00 | 0·00 |
| CIN2/3 | 5 | 40 | 115653 | 35 (25 to 47)  (25 to 47) | 5·43 (0·246) | 0·00 | 0·00 |
| *Studies with lag period between CIN diagnosis and cancer* | 4 |  |  | 33 (24 to 45)  (24 to 45) | 1·30 (0·730) | 0·00 | 0·00 |
| *Studies with histological diagnosis* | 4 |  |  | 33 (24 to 45)  (24 to 45) | 1·30 (0·730) | 0·00 | 0·00 |
| *Studies with low or moderate risk of bias* | 4 |  |  | 36 (24 to 52)  (24 to 52) | 5·38 (0·146) | 0·00 | 0·00 |
| *Studies without untreated women* | 4 |  |  | 34 (23 to 51)  (23 to 51) | 5·42 (0·143) | 0·00 | 0·00 |
| *Studies without hysterectomies before cancer diagnosis* | 4 |  |  | 36 (24 to 52)  (24 to 52) | 5·38 (0·146) | 0·00 | 0·00 |
| *Europe* | 3 |  |  | 31 (20 to 47)  (20 to 47) | 1·14 (0·566) | 0·00 | 0·00 |
| **Length of follow-up after CIN treatment** |  |  |  |  |  |  |  |
| 0-10y | 3 | 186 | 490087 | 38 (33 to 44)  (33 to 44) | 0·25 (0·881) | 0·00 | 0·00 |
| 10-20y | 1 | 63 | 200734 | 31 (24 to 40)  (24 to 40) | N/A (N/A) | N/A | N/A |
| >20y | 1 | 23 | 72523 | 32 (20 to 46)  (20 to 46) | N/A (N/A) | N/A | N/A |
| 0-5y | 2 | 86 | 238599 | 36 (29 to 45)  (29 to 45) | 2·06 (0·151) | 0·00 | 0·00 |
| 5-10y | 2 | 85 | 209024 | 41 (33 to 50)  (33 to 50) | 0·92 (0·338) | 0·00 | 0·00 |
| 10-15y | 1 | 40 | 124966 | 32 (23 to 43)  (23 to 43) | N/A (N/A) | N/A | N/A |
| 15-20y | 1 | 23 | 75768 | 30 (19 to 44)  (19 to 44) | N/A (N/A) | N/A | N/A |
| 0-20y | 2 | 359 | 983294 | 37 (33 to 40)  (33 to 40) | 0·04 (0·836) | 0·00 | 0·00 |

Abbreviations:

CI: confidence interval; CIN: cervical intraepithelial neoplasia; IR: incidence rate; N/A: not available; PI: prediction interval; y: years
