# Supplementary material for: Incidence and mortality from cervical cancer and other malignancies after treatment of cervical intraepithelial neoplasia: a systematic review and meta-analysis of the literature
Source: Ann Oncol. 2020 Feb;31(2):213–27. doi: 10.1016/j.annonc.2019.11.004 (PMC7479506; doi:10.1016/j.annonc.2019.11.004)
Supplement: Supplementary Table S5 [file mmc9.docx]

**Supplementary Table 5:** Pooled relative incidence of cervical cancer as compared to the reference population. Subgroup analyses according to age at CIN treatment; treatment method; CIN grade; length of follow-up. Sensitivity analyses including only studies at low or moderate risk of bias in all domains; with lag period; with histological diagnosis of CIN; without women with untreated CIN; without women treated with hysterectomy before cancer diagnosis; according to continent; according to type of effect estimate.

| **Cervical Cancer** | **Studies** | **Cases** | **Total**  **Population** | **RR (95% CI)**  **(95% PI)** | ***P*-value** | **Q-test (*P*-value)** | **I^2^ (95% CI)** | **τ^2^ (95% CI)** |
| --- | --- | --- | --- | --- | --- | --- | --- | --- |
| **Overall incidence** | 9 | 1145 | 229118 | 3·30 (2·57 to 4·24)  (1·73 to 6·30) | <0·001 | 61·33 (<0·001) | 82·94 (58·66-96·34) | 0·07 (0·02-0·36) |
| *Studies with lag period between CIN diagnosis and cancer* | 7 |  |  | 3·31 (2·33 to 4·69)  (1·49 to 7·32) | <0·001 | 32·39 (<0·001) | 76·08 (40·30 to 95·92) | 0·08 (0·02 to 0·62) |
| *Studies with histological diagnosis* | 9 |  |  | 3·16 (2·52 to 3·97)  (1·68 to 5·93) | <0·001 | 56·86 (<0·001) | 78·18 (51·51 to 95·46) | 0·05 (0·01 to 0·29) |
| *Studies excluding untreated CIN* | 8 |  |  | 3·13 (2·41 to 4·06)  (1·67 to 5·93) | <0·001 | 56·16 (<0·001) | 82·61 (57·08 to 97·18) | 0·06 (0·02 to 0·43) |
| *Studies excluding hysterectomies before cancer diagnosis* | 4 |  |  | 2·90 (1·85 to 4·56)  (1·28 to 6·55) | <0·001 | 8·34 (0·039) | 66·07 (0·00 to 98·10) | 0·12 (0·00 to 3·20) |
| *Europe* | 6 |  |  | 3·03 (2·18 to 4·22)  (1·40 to 6·57) | <0·001 | 29·75 (<0·001) | 85·35 (54·08 to 97·95) | 0·07 (0·01 to 0·61) |
| *Northern Europe* | 3 |  |  | 3·26 (0·70 to 15·08)  (0·17 to 61·40) | 0·080 | 20·26 (<0·001) | 90·80 (63·77 to 99·77) | 0·34 (0·06 to 15·21) |
| *Western Europe* | 3 |  |  | 2·90 (1·40 to 6·04)  (0·77 to 10·94) | 0·019 | 8·34 (0·039) | 66·06 (0·00 to 98·10) | 0·12 (0·00 to 3·20) |
| *SIR as effect estimate* | 5 |  |  | 3·39 (2·54 to 4·53)  (1·86 to 6·17) | <0·001 | 17·08 (0·001) | 75·34 (25·69 to 97·86) | 0·04 (0·00 to 0·53) |
| *RR/HR as effect estimate* | 4 |  |  | 3·36 (1·53 to 7·39)  (0·78 to 14·48) | 0·016 | 15·77 (0·001) | 71·43 (25·58 to 98·02) | 0·15 (0·02 to 2·95) |
| **Age at CIN treatment** |  |  |  |  |  |  |  |  |
| <50y | 2 | 29 | 2345 | 4·01 (1·47 to 10·95)  (1·47 to 10·95) | 0·036 | 0·19 (0·661) | 0·00 (0·00 to 99·49) | 0·00 (0·00 to 12·94) |
| ≥50y | 2 | 8 | 313 | 7·15 (4·75 to 10·76)  (4·75 to 10·76) | 0·010 | 0·01 (0·933) | 0·00 (0·00 to 86·25) | 0·00 (0·00 to 2·21) |
| **Treatment method for CIN** |  |  |  |  |  |  |  |  |
| Excision | 3 | 260 | 77657 | 2·04 (1·88 to 2·21)  (1·88 to 2·21) | <0·001 | 0·17 (0·920) | 0·00 (0·00 to 83·06) | 0·00 (0·00 to 0·26) |
| Ablation | 2 | 6 | 8683 | FE: 2·69 (0·94 to 7·65)  (NA)* | FE: 0·064* | 8·52 (0·004) | 88·26 (41·02 to 99·40) | 4·54 (0·42 to 100·00) |
| **CIN grade** |  |  |  |  |  |  |  |  |
| CIN1 | 1 | 8 | 2440 | 3·10 (1·40 to 6·20)  (1·40 to 6·20) | 0·003 | N/A (N/A) | N/A (N/A) | N/A (N/A) |
| CIN2 | 1 | 3 | 1541 | 3·70 (0·80 to 10·90)  (0·80 to 10·90) | 0·050 | N/A (N/A) | N/A (N/A) | N/A (N/A) |
| CIN2/3 | 2 | 21 | 9648 | 3·42 (0·22 to 52·64)  (0·22 to 52·64) | 0·110 | 0·37 (0·542) | 0·00 (0·00 to 99·74) | 0·00 (0·00 to 96·32) |
| CIN3 | 6 | 946 | 178919 | 3·09 (2·18 to 4·39)  (1·36 to 7·03) | <0·001 | 55·66 (<0·001) | 89·62 (70·17 to 98·62) | 0·08 (0·02 to 0·68) |
| *SIR as effect estimate* | 5 |  |  | 3·44 (2·54 to 4·67)  (1·88 to 6·30) | <0·001 | 16·90 (0·002) | 73·96 (23·71 to 98·30) | 0·04 (0·00 to 0·72) |
| *Studies with lag period between CIN diagnosis and cancer* | 4 |  |  | 2·93 (1·52 to 5·63)  (0·82 to 10·39) | 0·014 | 21·78 (<0·001) | 86·37 (49·55 to 99·23) | 0·12 (0·02 to 2·35) |
| *Studies with histological diagnosis* | 5 |  |  | 2·88 (2·02 to 4·10)  (1·34 to 6·23) | 0·001 | 50·30 (<0·001) | 88·47 (69·48 to 98·70) | 0·06 (0·02 to 0·60) |
| *Studies excluding untreated CIN* | 5 |  |  | 2·88 (2·02 to 4·10)  (1·34 to 6·23) | 0·001 | 50·30 (<0·001) | 88·47 (69·48 to 98·70) | 0·06 (0·02 to 0·60) |
| *Europe* | 5 |  |  | 2·87 (1·89 to 4·34)  (1·19 to 6·91) | 0·001 | 24·75 (<0·001) | 87·12 (51·96 to 98·96) | 0·08 (0·01 to 1·00) |
| *Northern Europe* | 3 |  |  | 2·47 (1·30 to 4·66)  (0·85 to 7·12) | 0·026 | 12·44 (0·002) | 73·16 (26·01 to 99·29) | 0·04 (0·01 to 1·99) |
| **Length of follow to up after CIN treatment** |  |  |  |  |  |  |  |  |
| 0-10y | 5 | 212 | 76437 | 3·60 (1·88 to 6·88)  (0·90 to 14·30) | 0·005 | 26·67 (<0·001) | 78·77 (44·70 to 97·45) | 0·19 (0·04 to 1·98) |
| *Studies with histological diagnosis* | 4 |  |  | 2·85 (1·58 to 5·15)  (1·00 to 8·14) | 0·011 | 10·54 (0·015) | 58·82 (6·14 to 97·09) | 0·07 (0·00 to 1·73) |
| *Studies without untreated CIN* | 3 |  |  | 2·76 (1·09 to 6·96)  (0·54 to 14·04) | 0·042 | 9·10 (0·011) | 71·64 (13·78 to 99·24) | 0·10 (0·01 to 5·01) |
| *Studies excluding hysterectomies before diagnosis of cancer* | 3 |  |  | 2·26 (1·25 to 4·10)  (0·99 to 5·17) | 0·027 | 2·47 (0·291) | 18·33 (0·00 to 98·59) | 0·02 (0·00 to 5·57) |
| *Europe* | 4 |  |  | 3·54 (1·44 to 8·73)  (0·54 to 23·04) | 0·021 | 25·55 (<0·001) | 86·40 (56·73 to 99·06) | 0·27 (0·05 to 4·39) |
| *RR/HR as effect estimate* | 3 |  |  | 3·01 (1·00 to 9·00)  (0·45 to 19·91) | 0·050 | 10·27 (0·006) | 72·44 (18·86 to 99·31) | 0·13 (0·01 to 6·96) |
| 10-20y | 3 | 75 | 32391 | 1·98 (1·11 to 3·52)  (1·03 to 3·81) | 0·036 | 2·17 (0·339) | 4·45 (0·00 to 96·06) | 0·01 (0·00 to 2·79) |
| >20y | 2 | 24 | 11957 | 2·40 (0·83 to 6·93)  (0·83 to 6·93) | 0·061 | 0·16 (0·687) | 0·00 (0·00 to 99·07) | 0·00 (0·00 to 100·00) |
| 0-5y | 2 | 86 | 62122 | FE: 2·31 (1·86 to 2·87)  (NA)* | FE: <0·001* | 8·94 (0·003) | 88·81 (43·78 to 99·92) | 0·66 (0·06 to 100·00) |
| 5-10y | 2 | 80 | 46544 | FE: 2·25 (1·80 to 2·82)  (NA)* | FE: <0·001* | 10·05 (0·002) | 90·05 (50·00 to 99·90) | 0·86 (0·10 to 100·00) |
| 10-15y | 2 | 41 | 32391 | 1·76 (1·69 to 1·83)  (1·69 to 1·83) | 0·003 | 0·00 (0·985) | 0·00 (0·00 to 0·00) | 0·00 (0·00 to 0·00) |
| 15-20y | 2 | 24 | 20313 | 1·96 (0·94 to 4·10)  (0·94 to 4·10) | 0·055 | 0·08 (0·778) | 0·00 (0·00 to 98·77) | 0·00 (0·00 to 67·70) |
| 0-20y | 3 | 252 | 69588 | 3·26 (0·70 to 15·08)  (0·17 to 61·40) | 0·080 | 20·26 (<0·001) | 90·80 (63·77 to 99·77) | 0·34 (0·06 to 15·21) |

*FE results presented, because random-effects meta-analysis yielded too wide and uninformative CIs

Abbreviations:

CI: confidence interval; CIN: cervical intraepithelial neoplasia; FE: fixed-effect meta-analysis; N/A: not available; PI: prediction interval; RR: relative risk; y: years
