# Supplementary material for: Incidence and mortality from cervical cancer and other malignancies after treatment of cervical intraepithelial neoplasia: a systematic review and meta-analysis of the literature
Source: Ann Oncol. 2020 Feb;31(2):213–27. doi: 10.1016/j.annonc.2019.11.004 (PMC7479506; doi:10.1016/j.annonc.2019.11.004)
Supplement: Supplementary Table S6 [file mmc10.docx]

**Supplementary Table 6:** Pooled relative risk of cancer incidence and mortality as compared to the reference population. Sensitivity analyses including only studies at low or moderate risk of bias in all domains; with lag period; with histological diagnosis of CIN; without women with untreated CIN; without women treated with hysterectomy before cancer diagnosis; according to continent; according to type of effect estimate.

| **Cancer incidence or mortality** | **Studies** | **Outcomes** | **Women** | **RR (95% CI)**  **(95% PI)** | ***P*-value** | **Q-test (*P*-value)** | **I^2^ (95% CI)** | **τ^2^ (95% CI)** |
| --- | --- | --- | --- | --- | --- | --- | --- | --- |
| **Cervical cancer incidence** | 9 | 1145 | 229118 | 3·30 (2·57 to 4·24)  (1·73 to 6·30) | <0·001 | 61·33 (<0·001) | 82·94 (58·66 to 96·34) | 0·07 (0·02 to 0·36) |
| *Studies with lag period between CIN diagnosis and cancer* | 7 |  |  | 3·31 (2·33 to 4·69)  (1·49 to 7·32) | <0·001 | 32·39 (<0·001) | 76·08 (40·30 to 95·92) | 0·08 (0·02 to 0·62) |
| *Studies with histological diagnosis* | 9 |  |  | 3·16 (2·52 to 3·97)  (1·68 to 5·93) | <0·001 | 56·86 (<0·001) | 78·18 (51·51 to 95·46) | 0·05 (0·01 to 0·29) |
| *Studies excluding untreated CIN* | 8 |  |  | 3·13 (2·41 to 4·06)  (1·67 to 5·93) | <0·001 | 56·16 (<0·001) | 82·61 (57·08 to 97·18) | 0·06 (0·02 to 0·43) |
| *Studies excluding hysterectomies before cancer diagnosis* | 4 |  |  | 2·90 (1·85 to 4·56)  (1·28 to 6·55) | <0·001 | 8·34 (0·039) | 66·07 (0·00 to 98·10) | 0·12 (0·00 to 3·20) |
| *Europe* | 6 |  |  | 3·03 (2·18 to 4·22)  (1·40 to 6·57) | <0·001 | 29·75 (<0·001) | 85·35 (54·08 to 97·95) | 0·07 (0·01 to 0·61) |
| *Northern Europe* | 3 |  |  | 3·26 (0·70 to 15·08)  (0·17 to 61·40) | 0·080 | 20·26 (<0·001) | 90·80 (63·77 to 99·77) | 0·34 (0·06 to 15·21) |
| *Western Europe* | 3 |  |  | 2·90 (1·40 to 6·04)  (0·77 to 10·94) | 0·019 | 8·34 (0·039) | 66·06 (0·00 to 98·10) | 0·12 (0·00 to 3·20) |
| *SIR as effect estimate* | 5 |  |  | 3·39 (2·54 to 4·53)  (1·86 to 6·17) | <0·001 | 17·08 (0·001) | 75·34 (25·69 to 97·86) | 0·04 (0·00 to 0·53) |
| *RR/HR as effect estimate* | 4 |  |  | 3·24 (2·05 to 5·13)  (0·78 to 14·48) | <0·001 | 15·77 (0·001) | 71·43 (25·58 to 98·02) | 0·15 (0·02 to 2·95) |
| **Cervical & vaginal cancer incidence** | 4 | 1503 | 237350 | 5·71 (1·18 to 27·58)  (0·27 to 121·00) | 0·039 | 34·52 (<0·001) | 82·43 (53·43 to 98·69) | 0·68 (0·17 to 10·82) |
| *Studies with histological diagnosis* | 3 |  |  | 6·43 (0·41 to 100·26)  (0·04 to 1025·91) | 0·100 | 34·27 (<0·001) | 89·86 (66·69 to 99·73) | 0·98 (0·22 to 41·15) |
| *Studies with low/moderate risk of bias* | 3 |  |  | 2·39 (2·14 to 2·67)  (2·14 to 2·67) | 0·001 | 1·61 (0·448) | 0·00 (0·00 to 96·68) | 0·00 (0·00 to 11·52) |
| *Studies excluding hysterectomies before diagnosis of cancer* | 2 |  |  | FE: 2·43 (2·29 to 2·57)  (NA)* | FE: <0·001* | 3·09 (0·079) | 67·61 (0·00 to 99·58) | 0·87 (0·00 to 100·00) |
| *Europe* | 3 |  |  | 2·39 (2·14 to 2·67)  (2·14 to 2·67) | 0·001 | 1·61 (0·448) | 0·00 (0·00 to 96·68) | 0·00 (0·00 to 11·52) |
| **Vaginal cancer incidence** | 6 | 329 | 518516 | 10·84 (5·58 to 21·10)  (2·46 to 47·47) | <0·001 | 33·20 (<0·001) | 81·56 (42·45 to 98·43) | 0·26 (0·04 to 3·75) |
| *Studies with lag period between CIN diagnosis and cancer* | 4 |  |  | 9·92 (2·76 to 35·57)  (0·80 to 122·48) | 0·011 | 7·78 (0·051) | 82·88 (0·00 to 99·37) | 0·46 (0·00 to 15·13) |
| *Studies with histological diagnosis* | 5 |  |  | 11·41 (4·66 to 27·94)  (1·63 to 79·84) | 0·002 | 33·19 (<0·001) | 88·20 (52·95 to 99·23) | 0·38 (0·06 to 6·59) |
| *Studies without untreated CIN* | 4 |  |  | 12·72 (3·43 to 47·18)  (0·95 to 170·09) | 0·009 | 31·81 (<0·001) | 90·69 (56·60 to 99·62) | 0·49 (0·07 to 13·38) |
| *Europe* | 5 |  |  | 12·06 (5·28 to 27·54)  (2·14 to 68·03) | 0·001 | 37·20 (<0·001) | 84·56 (47·01 to 99·06) | 0·30 (0·05 to 5·75) |
| *Northern Europe* | 3 |  |  | 7·64 (3·65 to 15·96)  (2·60 to 22·39) | 0·007 | 3·26 (0·196) | 28·63 (0·00 to 97·60) | 0·03 (0·00 to 3·37) |
| *SIR as effect estimate* | 4 |  |  | 9·47 (4·09 to 21·95)  (1·82 to 49·30) | 0·003 | 28·23 (<0·001) | 81·97 (50·24 to 98·67) | 0·20 (0·04 to 3·25) |
| RR/HR as effect estimate | 2 |  |  | FE: 13·80 (7·49 to 25·42)  (NA)* | FE: <0·001* | 2·51 (0·113) | 60·22 (0·00 to 99·44) | 0·85 (0·00 to 100·00) |
| **Vulvar cancer incidence** | 7 | 455 | 511315 | 3·34 (2·39 to 4·67)  (1·55 to 7·17) | <0·001 | 19·99 (0·003) | 63·99 (19·50 to 92·92) | 0·08 (0·01 to 0·58) |
| *Studies with lag period between CIN diagnosis and cancer* | 5 |  |  | 2·84 (2·01 to 4·03)  (1·52 to 5·31) | 0·001 | 8·95 (0·062) | 46·51 (0·00 to 93·00) | 0·04 (0·00 to 0·54) |
| *Studies with histological diagnosis* | 5 |  |  | 3·18 (2·11 to 4·77)  (1·42 to 7·10) | 0·001 | 13·82 (0·008) | 60·94 (12·43 to 94·91) | 0·06 (0·01 to 0·74) |
| *Studies with low/moderate risk of bias* | 5 |  |  | 3·48 (2·34 to 5·19)  (1·45 to 8·33) | <0·001 | 19·84 (0·001) | 67·58 (24·76 to 94·76) | 0·09 (0·01 to 0·80) |
| *Studies without untreated CIN* | 5 |  |  | 3·57 (2·13 to 6·00)  (1·16 to 10·91) | 0·002 | 19·99 (<0·001) | 76·36 (36·16 to 97·24) | 0·13 (0·02 to 1·39) |
| *Europe* | 6 |  |  | 3·44 (2·29 to 5·16)  (1·39 to 8·50) | 0·001 | 19·99 (0·001) | 69·88 (26·95 to 95·21) | 0·10 (0·02 to 0·85) |
| *Northern Europe* | 3 |  |  | 3·24 (0·89 to 11·82)  (0·30 to 34·86) | 0·059 | 9·94 (0·007) | 80·64 (24·81 to 99·53) | 0·21 (0·02 to 10·96) |
| *Western Europe* | 3 |  |  | 3·79 (1·82 to 7·89)  (1·25 to 11·49) | 0·016 | 3·47 (0·176) | 43·04 (0·00 to 98·58) | 0·04 (0·00 to 3·44) |
| *SIR as effect estimate* | 3 |  |  | 4·18 (1·71 to 10·26)  (1·02 to 17·15) | 0·021 | 3·69 (0·158) | 49·12 (0·00 to 98·81) | 0·06 (0·00 to 5·52) |
| *RR/HR as effect estimate* | 4 |  |  | 2·88 (1·74 to 4·75)  (1·15 to 7·19) | 0·007 | 8·78 (0·032) | 60·13 (0·00 to 97·04) | 0·06 (0·00 to 1·26) |
| **Anal cancer incidence** | 7 | 534 | 635390 | 5·11 (2·73 to 9·55)  (1·04 to 25·03) | <0·001 | 132·63 (<0·001) | 92·42 (80·10 to 98·63) | 0·36 (0·12 to 2·11) |
| *Studies with lag period between CIN diagnosis and cancer* | 5 |  |  | 5·00 (1·86 to 13·40)  (0·51 to 49·40) | 0·011 | 129·21 (<0·001) | 96·03 (87·47 to 99·57) | 0·55 (0·16 to 5·32) |
| *Studies with histological diagnosis* | 6 |  |  | 5·22 (2·48 to 10·95)  (0·83 to 32·79) | 0·002 | 131·22 (<0·001) | 94·54 (84·31 to 99·21) | 0·43 (0·13 to 3·11) |
| *Studies without untreated women* | 5 |  |  | 6·39 (2·97 to 13·78)  (1·15 to 35·52) | 0·003 | 107·85 (<0·001) | 92·90 (79·97 to 99·20) | 0·31 (0·09 to 2·91) |
| *SIR as effect estimate* | 4 |  |  | 5·72 (1·26 to 25·91)  (0·26 to 126·54) | 0·035 | 34·31 (<0·001) | 91·37 (68·04 to 99·44) | 0·72 (0·15 to 12·13) |
| *RR/HR as effect estimate* | 3 |  |  | 4·42 (3·13 to 6·25)  (3·13 to 6·25) | 0·003 | 1·90 (0·386) | 0·00 (0·00 to 96·76) | 0·00 (0·00 to 1·02) |
| *Europe* | 5 |  |  | 4·56 (3·74 to 5·58)  (3·74 to 5·58) | <0·001 | 3·54 (0·472) | 0·00 (0·00 to 87·73) | 0·00 (0·00 to 0·32) |
| *Northern Europe* | 3 |  |  | 1·84 (1·38 to 2·46)  (0·38 to 8·99) | <0·001 | 1382·99 (<0·001) | 98·96 (98·12 to 99·38) | 0·59 (0·35 to 1·09) |
| **Female HPV-related anogenital (anal-vulvar-vaginal-cervical) cancer incidence** | 7 | 1360 | 548316 | 3·69 (2·29 to 5·94)  (1·45 to 9·39) | <0·001 | 15·54 (0·016) | 45·27 (3·25 to 91·92) | 0·11 (0·00 to 1·48) |
| *Studies with lag period between CIN diagnosis and cancer* | 5 |  |  | 3·44 (1·70 to 6·95)  (0·91 to 12·94) | 0·008 | 12·57 (0·014) | 58·53 (6·46 to 97·49) | 0·17 (0·01 to 4·55) |
| *Studies with histological diagnosis* | 6 |  |  | 3·70 (2·07 to 6·64)  (1·17 to 11·70) | 0·002 | 15·04 (0·010) | 54·25 (7·78 to 95·53) | 0·15 (0·01 to 2·67) |
| *Studies without untreated women* | 5 |  |  | 4·73 (3·17 to 7·04)  (3·16 to 7·03) | <0·001 | 2·20 (0·698) | 0·00 (0·00 to 95·39) | 0·00 (0·00 to 4·25) |
| *Europe* | 6 |  |  | 4·73 (3·52 to 6·36)  (3·52 to 6·36) | <0·001 | 2·20 (0·820) | 0·00 (0·00 to 89·79) | 0·00 (0·00 to 1·68) |
| *Northern Europe* | 4 |  |  | 4·18 (2·72 to 6·41)  (2·72 to 6·41) | 0·002 | 1·20 (0·753) | 0·00 (0·00 to 97·69) | 0·00 (0·00 to 8·91) |
| *SIR as effect estimate* | 4 |  |  | 3·00 (1·09 to 8·31)  (0·54 to 16·84) | 0·041 | 7·36 (0·061) | 50·82 (0·00 to 98·27) | 0·19 (0·00 to 10·50) |
| *RR/HR as effect estimate* | 3 |  |  | 4·78 (2·94 to 7·76)  (2·94 to 7·76) | 0·005 | 0·60 (0·742) | 0·00 (0·00 to 91·21) | 0·00 (0·00 to 1·35) |
| **Endometrial cancer incidence** | 5 | 168 | 181702 | 0·81 (0·62 to 1·07)  (0·56 to 1·19) | 0·105 | 4·70 (0·320) | 17·42 (0·00 to 93·93) | 0·01 (0·00 to 0·63) |
| *Studies with histological diagnosis* | 4 |  |  | 0·78 (0·55 to 1·10)  (0·49 to 1·24) | 0·106 | 3·53 (0·317) | 19·39 (0·00 to 97·44) | 0·01 (0·00 to 1·46) |
| *North Europe* | 3 |  |  | 0·80 (0·43 to 1·48)  (0·32 to 2·01) | 0·258 | 3·34 (0·188) | 41·07 (0·00 to 98·59) | 0·03 (0·00 to 2·57) |
| **Ovarian cancer incidence** | 5 | 309 | 235532 | 1·02 (0·73 to 1·43)  (0·51 to 2·05) | 0·867 | 12·92 (0·012) | 69·20 (13·34 to 96·42) | 0·05 (0·00 to 0·59) |
| *Studies with histological diagnosis* | 4 |  |  | 1·06 (0·69 to 1·64)  (0·44 to 2·54) | 0·693 | 11·61 (0·009) | 75·72 (20·85 to 98·29) | 0·06 (0·00 to 1·04) |
| *Europe* | 4 |  |  | 0·94 (0·71 to 1·25)  (0·62 to 1·42) | 0·522 | 4·49 (0·213) | 28·30 (0·00 to 94·51) | 0·01 (0·00 to 0·39) |
| *Northern Europe* | 3 |  |  | 0·86 (0·59 to 1·24)  (0·59 to 1·24) | 0·210 | 1·60 (0·448) | 0·00 (0·00 to 96·07) | 0·00 (0·00 to 0·73) |
| **Breast cancer incidence** | 5 | 1396 | 181702 | 1·00 (0·94 to 1·06)  (0·94 to 1·06) | 0·975 | 1·90 (0·754) | 0·00 (0·00 to 89·74) | 0·00 (0·00 to 0·04) |
| *Studies with histological diagnosis* | 4 |  |  | 0·98 (0·93 to 1·04)  (0·93 to 1·04) | 0·364 | 0·76 (0·860) | 0·00 (0·00 to 94·16) | 0·00 (0·00 to 0·09) |
| *Northern Europe* | 3 |  |  | 1·00 (0·88 to 1·14)  (0·88 to 1·14) | 0·930 | 1·26 (0·532) | 0·00 (0·00 to 95·95) | 0·00 (0·10 to 0·00) |
| **Lung cancer incidence** | 5 | 700 | 209710 | 1·82 (1·32 to 2·52)  (0·89 to 3·74) | 0·007 | 26·17 (<0·001) | 85·67 (55·22 to 98·54) | 0·05 (0·01 to 0·61) |
| *Studies with histological diagnosis* | 4 |  |  | 1·91 (1·31 to 2·78)  (0·86 to 4·23) | 0·012 | 23·45 (<0·001) | 87·25 (59·81 to 99·09) | 0·05 (0·01 to 0·77) |
| *Europe* | 4 |  |  | 1·98 (1·36 to 2·89)  (0·94 to 4·17) | 0·010 | 9·61 (0·022) | 80·62 (2·81 to 99·12) | 0·04 (0·00 to 1·10) |
| *Northern Europe* | 3 |  |  | 2·01 (0·94 to 4·31)  (0·50 to 8·09) | 0·059 | 4·99 (0·083) | 85·40 (0·00 to 99·74) | 0·07 (0·00 to 4·81) |
| **Colorectal cancer incidence** | 5 | 1059 | 331696 | 0·99 (0·93 to 1·07)  (0·93 to 1·07) | 0·854 | 2·74 (0·602) | 0·00 (0·00 to 92·48) | 0·00 (0·00 to 0·07) |
| *Studies with histological diagnosis* | 3 |  |  | 1·02 (0·86 to 1·21)  (0·86 to 1·21) | 0·694 | 1·64 (0·441) | 0·00 (0·00 to 96·92) | 0·00 (0·00 to 0·22) |
| *SIR as effect estimate* | 4 |  |  | 1·00 (0·90 to 1·12)  (0·90 to 1·12) | 0·988 | 2·65 (0·448) | 0·00 (0·00 to 96·05) | 0·00 (0·00 to 0·20) |
| *Northern Europe* | 4 |  |  | 0·98 (0·93 to 1·03)  (0·93 to 1·03) | 0·284 | 0·69 (0·875) | 0·00 (0·00 to 93·07) | 0·00 (0·00 to 0·08) |
| *Both colon and rectal cancer included* | 4 |  |  | 1·00 (0·90 to 1·12) | 0·988 | 2·65 (0·448) | 0·00 (0·00 to 96·05) | 0·00 (0·00 to 0·20) |
| **Any cancer incidence** | 4 | 3124 | 125586 | 1·14 (0·98 to 1·32)  (0·84 to 1·54) | 0·074 | 31·76 (<0·001) | 84·49 (56·57 to 98·88) | 0·01 (0·00 to 0·11) |
| *Studies with histological diagnosis* | 3 |  |  | 1·14 (0·85 to 1·52)  (0·67 to 1·94) | 0·195 | 31·55 (<0·001) | 89·86 (64·88 to 99·75) | 0·01 (0·00 to 0·48) |
| **Cervical and/or vaginal cancer mortality** | 3 | 376 | 176782 | 5·04 (0·69 to 36·95)  (0·13 to 197·94) | 0·073 | 26·21 (<0·001) | 89·99 (61·87 to 99·77) | 0·51 (0·09 to 24·94) |

*FE results presented, because RE yielded too wide and uninformative CIs

Abbreviations:

CI: confidence interval; FE: fixed-effect meta-analysis; N/A: not available; PI: prediction interval; RR: relative risk
